# Supplementary material for: Durvalumab plus pazopanib combination in patients with advanced soft tissue sarcomas: a phase II trial
Source: Nat Commun. 2024 Jan 23;15:685. doi: 10.1038/s41467-024-44875-2 (PMC10806253; doi:10.1038/s41467-024-44875-2)
Supplement: Supplementary file 1 — Supplementary Information [file 41467_2024_44875_MOESM1_ESM.pdf]

Supplementary Information

Supplementary Methods

Whole-exome sequencing for tumor tissue

For the generation of standard exome capture libraries, we used the Agilent SureSelect Target Enrichment protocol for Illumina paired-end sequencing library (Version C2, December 2018) together with 1ug input gDNA. In all cases, the SureSelect Human All Exon V6 probe set was used. The quantification of DNA and the DNA quality is measured by PicoGreen and agarose gel electrophoresis. We used 200ng of DNA diluted in EB Buffer and sheared to a target peak size of 150–200 bp using the Covaris LE220 focused-ultrasonicator (Covaris, Woburn, MA) according to the manufacturer's recommendations. Load the 8 microTUBE Strip into the tube holder of the ultrasonicator and shear the DNA using the following settings: mode, frequency sweeping; duty cycle, 10%; intensity, 5; cycles per burst, 200; duration, 60 sec × 6 cycles; temperature, 4°C–7°C. The fragmented DNA is repaired, an 'A' is ligated to the 3′ end, agilent adapters are then ligated to the fragments. Once ligation had been assessed, the adapter ligated product is PCR amplified. For exome capture, 250 ng of DNA library was mixed with hybridization buffers, blocking mixes, RNase block and 5 μl of SureSelect all exon capture library, according to the standard Agilent SureSelect Target Enrichment protocol. Hybridization to the capture baits was conducted at 65°C using heated thermal cycler lid option at 105°C for 24 hours on PCR machine. The captured DNA was then washing and amplified. The final purified product is then quantified using qPCR according to the qPCR Quantification Protocol Guide Guide (KAPA Library Quantificatoin kits for Illumina Sequencing platforms) and qualified using the TapeStation DNA screentape D1000 (Agilent). Then we sequenced using the HiSeq™ 2500 platform (Illumina,San Diego, USA)

WES data analysis

Fastq files generated by WES were subjected into quality control (FastQC) and then aligned on human genome (hg19) and sorted by genomic coordinates using Burrows-Wheeler Aligner (BWA) (version 0.7.12-r1039, BWA-MEM algorithm)[1] and SAMtools (v0.1.19)[2], respectively. Genome Analysis Toolkit (GATK, v3.6 and v.4.13) was used to perform duplicate marking, indel realignment, and base recalibration for coordinate-sorted hg19-aligned reads[3]. MuTect2 from GATKv4.13 was used to identify tumor-specific single nucleotide variants (SNVs) and small insertion and deletion (INDEL) by comparing BAM files of tumor and those of matched normal samples. If the called mutations were found in gnomAD[4] with higher population allele fraction than 2.5e-6, we assumed these events were possible germline and filtered them out. The resulting vcf files wer converted into maf files using vcf2maf tool (citation: Cyriac Kandoth. mskcc/vcf2maf: vcf2maf v1.6.19. (2020). doi:10.5281/zenodo.593251) with annotation by variant effect predictors (VEP)[5]. For further analysis, only mutations with altered reads ≥ 4 in tumor and altered reads < 2 in normal were kept.

The called somatic mutations by MuTect2 were used for identification of tumor neoantigen candidates. HLA typing for MHC class I was conducted by POLYSOLVER[6], and HLA alleles with 4-digit resolution were furtherly used. Then, we detected the expressed neoantigens using pVACseq pipeline (peptide length: 8-11 amino acids and expression cutoff: FPKM ≥ 1) with NetMHCpan4.0[7,8]. To explore whether LOH occurs in HLA genes, we used LOHHLA algorithm[9].

We used CNVkit[10] to obtain copy number variation (CNV), and the CNV information were summarized in segment file for each tumor sample. The CNVs were corrected by In Silico Admixture Removal (ISAR) method based on tumor purity and ploidy which were estimated by ABSOLUTE[11,12]. The corrected CNVs were applied to GISTIC2.0 to determine the

alterations in copy number per each gene[13]. Cancer cell fraction for each mutation was calculated by ABSOLUTE[12].

RNA-sequencing

Total RNA concentration was estimated by Quant-IT RiboGreen (Invitrogen). To determine the DV200 (% of RNA fragments > 200 bp) value, samples were run on the TapeStation RNA ScreenTape (Agilent). Overall, 100 ng of total RNA was subjected to sequencing library construction using a TruSeq RNA Access library prep kit (Illumina, San Diego, CA, USA) according to the manufacturer's protocol. Briefly, the total RNA was first fragmented into small pieces using divalent cations under elevated temperature. The cleaved RNA fragments were copied into first strand cDNA using SuperScript II reverse transcriptase (Invitrogen, #18064014) and random primers. This was followed by second strand cDNA synthesis using DNA polymerase I, RNase H, and dUTP. These cDNA fragments were subjected to an end-repair process, addition of a single 'A' base, and subsequently, ligation of the adapters. The products are then purified and enriched with PCR to create the cDNA library. All libraries were normalized and six were pooled into a single hybridization/capture reaction. Pooled libraries were incubated with a cocktail of biotinylated oligos, corresponding to coding regions of the genome. Targeted library molecules were captured via hybridized biotinylated oligo probes using streptavidin-conjugated beads. After two rounds of hybridization/capture reactions, the enriched library molecules were subjected to a second round of PCR amplification. The captured libraries were quantified using KAPA Library Quantification kits for Illumina Sequencing platforms according to the qPCR Quantification Protocol Guide (KAPA BIOSYSTEMS, #KK4854), and assessed using the TapeStation D1000 ScreenTape (Agilent Technologies, # 5067-5582). Indexed libraries were then submitted to an Illumina HiSeq2500 (Illumina, Inc., San Diego, CA, USA), and paired-end (2 × 101 bp) sequencing was

performed by Macrogen Incorporated.

RNA-Sequencing data analysis

Sequence reads from RNA-sequencing were aligned on hg19 by STAR_(v2.6.1d)[14], and reads counts per gene were obtained with DEGseq[15] (R package) followed by transcript per million (TPM) estimation. For further analysis based on gene expression profiling, log2-transformed TPM values were used. To estimate the Microenvironment Cell Populations-counter scores of tumor samples, MCPcounter (R package) was used, and five sarcoma immune class (SIC) was determined based on MCPcounter scores according to the publication by Petitprez et al[16,17]. Single sample GSEA (ssGSEA) algorithm in GSVA[18] (R package) was used to calculate geneset enrichment scores. Gene fusion detection was performed by STAR-Fusion (v1.5.0) with STAR-aligned bam files[19]. Cufflinks (v2.2.1) quantified the aligned reads in Fragments Per Kilobase Million (FPKM), which were used to identify expressed tumor neoantigens in pVACseq pipeline[7,20].

OPAL multiplex immunofluorescence (IF) staining and analysis

The tyramide signal amplification (TSA)-based Opal method was used in this study for immunofluorescence (IF) staining. All multiplexed staining was performed with the Opal 7 Immunology Discovery Kit (OP7DS2001KT; Akoya). The following primary antibodies were used: CD3 (1:50, UCHT1; Thermo Fisher), CD8 (1:200, 108M-96; Cell Marque), CD20 (1:200, M0755; DAKO) and PD1 (1:100, NAT105; Cell Marque). TSA visualization was performed using Opal fluorophores (Opal 520, Opal 570, Opal 620 and Opal 690). Multiplexed staining was finished with a DAPI counterstain and the slides were covered by using mounting solution (S3023; Dako). Opal multiplex IF Images of stained slides were acquired with the Vectra Polaris (Akoya Biosciences) whole-slide scanner using a standardized scanning protocol, and

processed using digital image analysis software, inform (Ver 2.5, Akoya Biosciences).

Immunohistochemistry (IHC), image acquisition and analysis.

The IHC staining for CD31 was performed using Benchmark® automatic immunostaining device (Roche Tissue Diagnostics, Tucson, USA) and an UltraViewTM Universal DAB Detection Kit (Ventana Medical Systems, Tucson, USA), according to the manufacturer's instructions. Four-micron-thick sections were immunostained with primary antibodies against CD31 (1:100, JC70A, Dako). The H&E staining and IHC staining slides were scanned using an PANNORAMIC 250 Flash III scanner (3DHistech Ltd., Sysmex Belgium NV) at x40 magnification. Manual annotation was carried out for tumor regions of interest (ROI) of all staining slides based on hematoxylin-eosin staining. The edges of the tissue were excluded from annotation because they were likely affected by staining artifacts. The CaseViewer v.2.4 and DensitoQuant application (3DHistech Ltd., Sysmex Belgium NV) were used for digital image analysis. DensitoQuant identifies the positive stain based on an automatic color separation method through which individual positive pixels are counted and classified as negative (< one-quarter of maximal intensity), weak- (one-quarter ~ one-half of maximal intensity), moderate- (one-half ~ three-quarters of maximal intensity), and strong (> three-quarters of maximal intensity) based on the intensity and threshold ranges.

Supplementary Figures

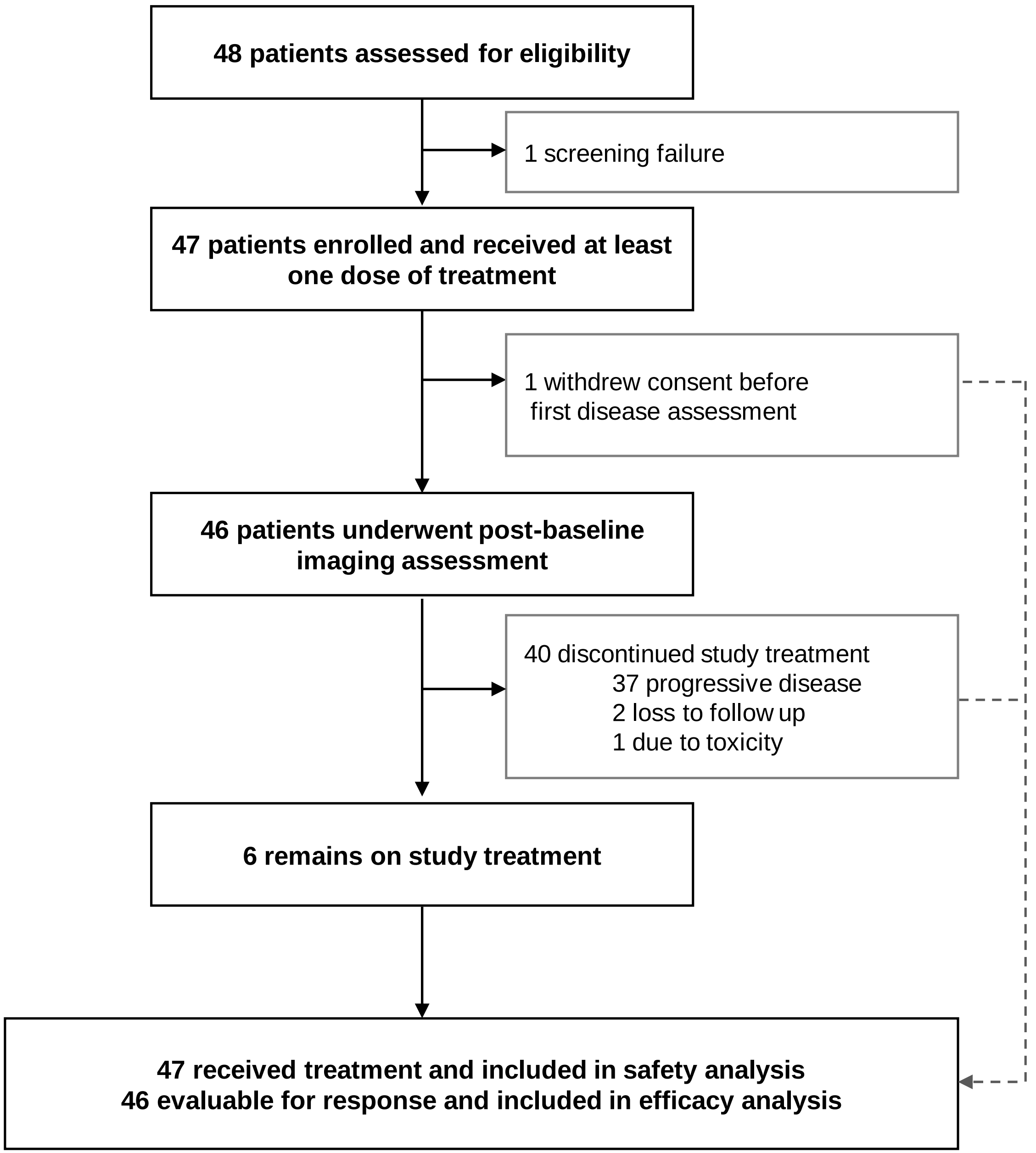

Supplementary Figure 1. Trial profile

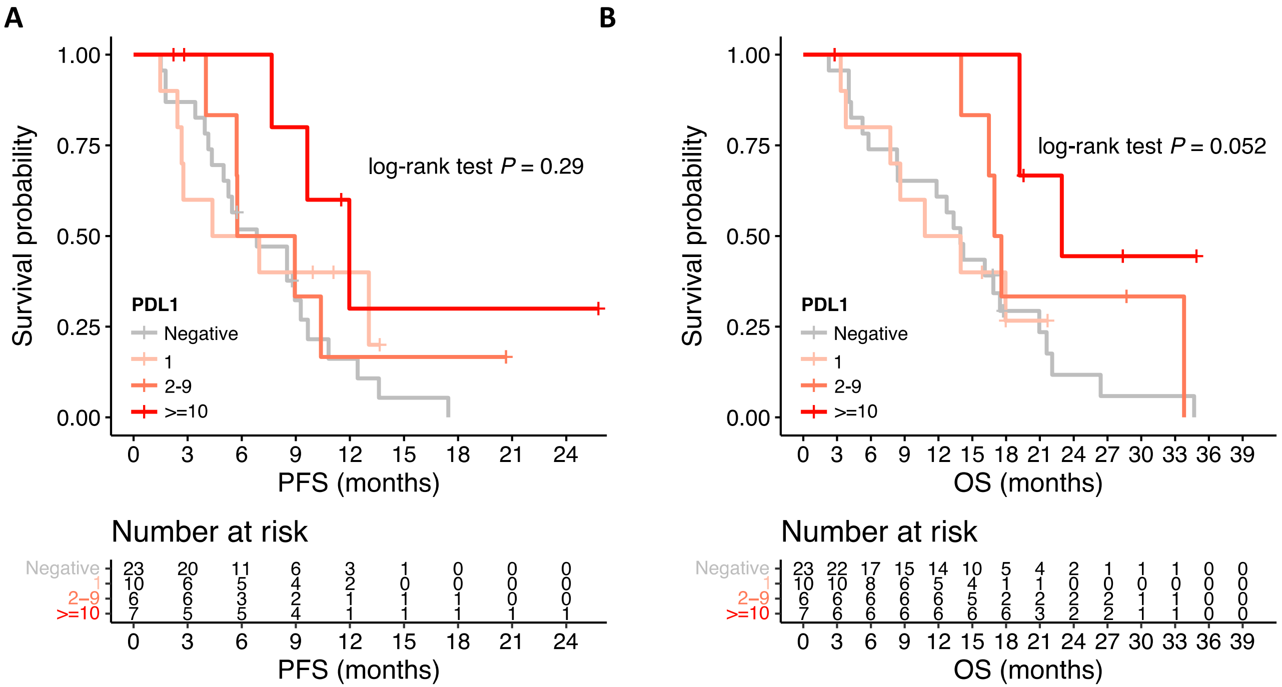

Supplementary Figure 2. (A) PFS and (B) OS according to PD-L1 status.
Source data are provided as a Source Data file.

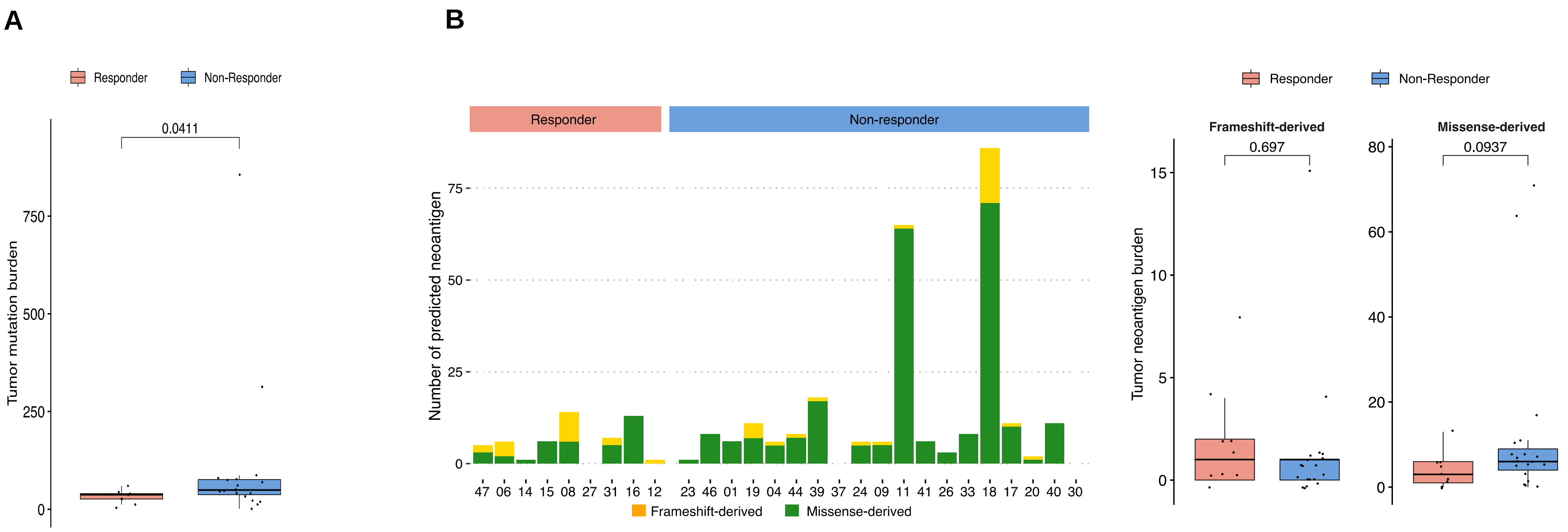

Supplementary Figure 3. Association between treatment responses and TMB (A) and neoantigen burden (B) (n=28)

The sample size, n of responders and non-responders are 9 and 19, respectively. P-values were calculated by two-sided Wilcoxon rank-sum tests. Center lines, upper and lower bounds of boxplots indicate the median, 25th, and 75th quantile, respectively. The whiskers of boxplots indicate 1.5 times of the interquartile range. Source data are provided as a Source Data file.

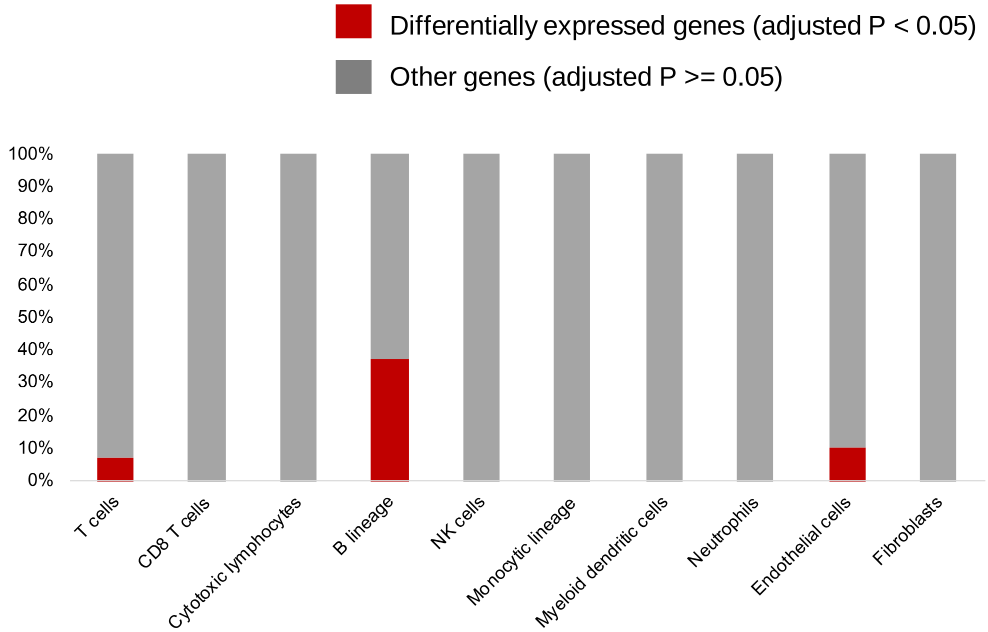

Cell type	N of significant marker genes	N of non-significant marker genes
T cells	1	13
CD8 T cells	0	1
Cytotoxic lymphocytes	0	7
B lineage	3	5
NK cells	0	8
Monocytic lineage	0	7
Myeloid dendritic cells	0	5
Neutrophils	0	14
Endothelial cells	3	27
Fibroblasts	0	8

Supplementary Figure 4. The percentage (left) and number (right) of differentially expressed marker genes used in MCP-counter per each cell type.

DESeq2 was used to identify differentially expressed marker genes between responders (n=9) and non-responders (n=19), and genes were defined as significant marker genes only if adjusted p-values from DESeq2 were less than 0.05. Source data are provided as a Source Data file.

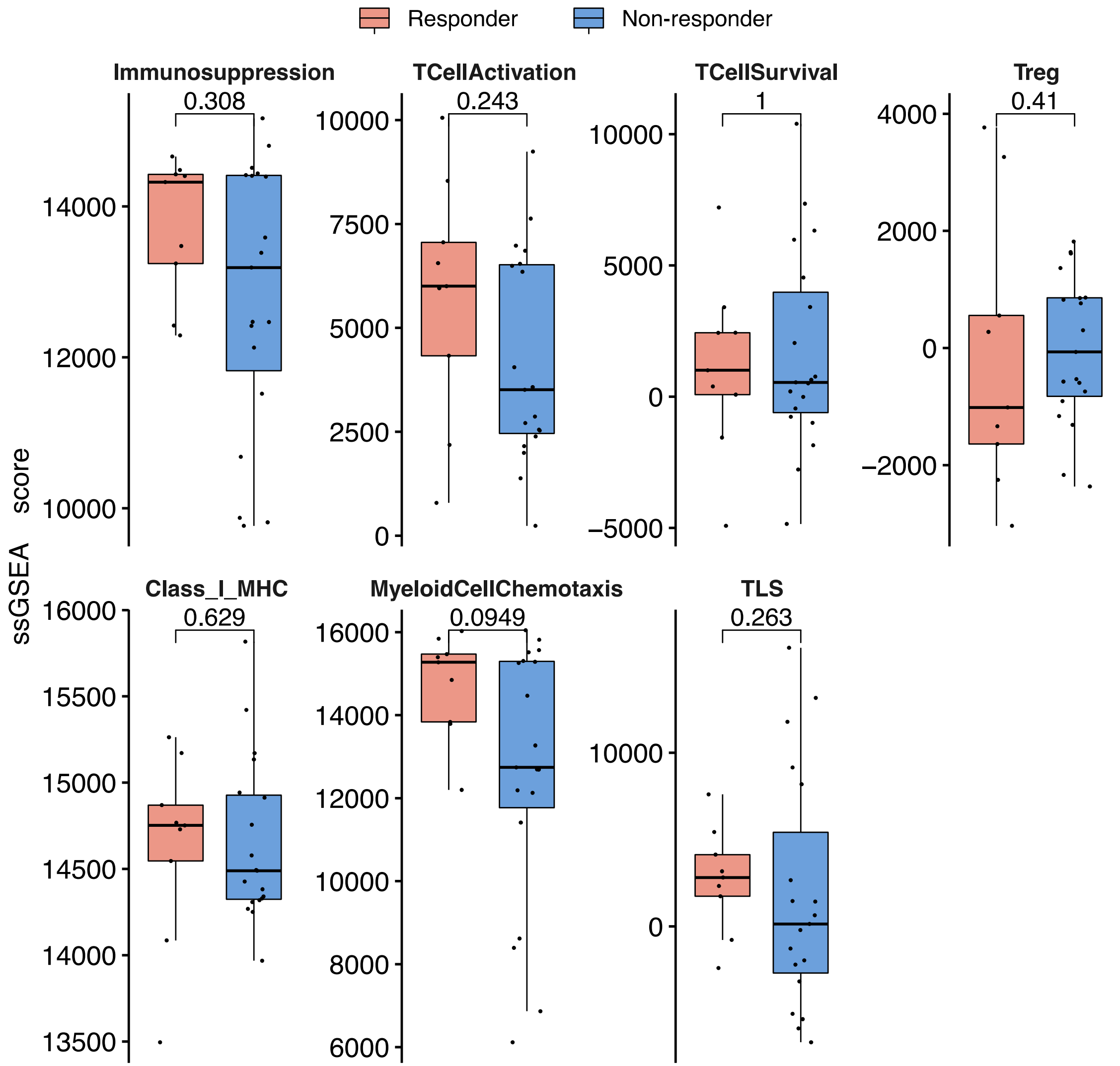

Supplementary Figure 5. ssGSEA scores of TME signatures compared between responders (n=9) and non-responders (n=19)

P-values were calculated by two-sided Wilcoxon rank-sum tests. Center lines, upper and lower bounds of boxplots indicate the median, 25th, and 75th quantile, respectively. The whiskers of boxplots indicate 1.5 times of the interquartile range. ssGSEA; single sample gene set enrichment analysis. Source data are provided as a Source Data file.

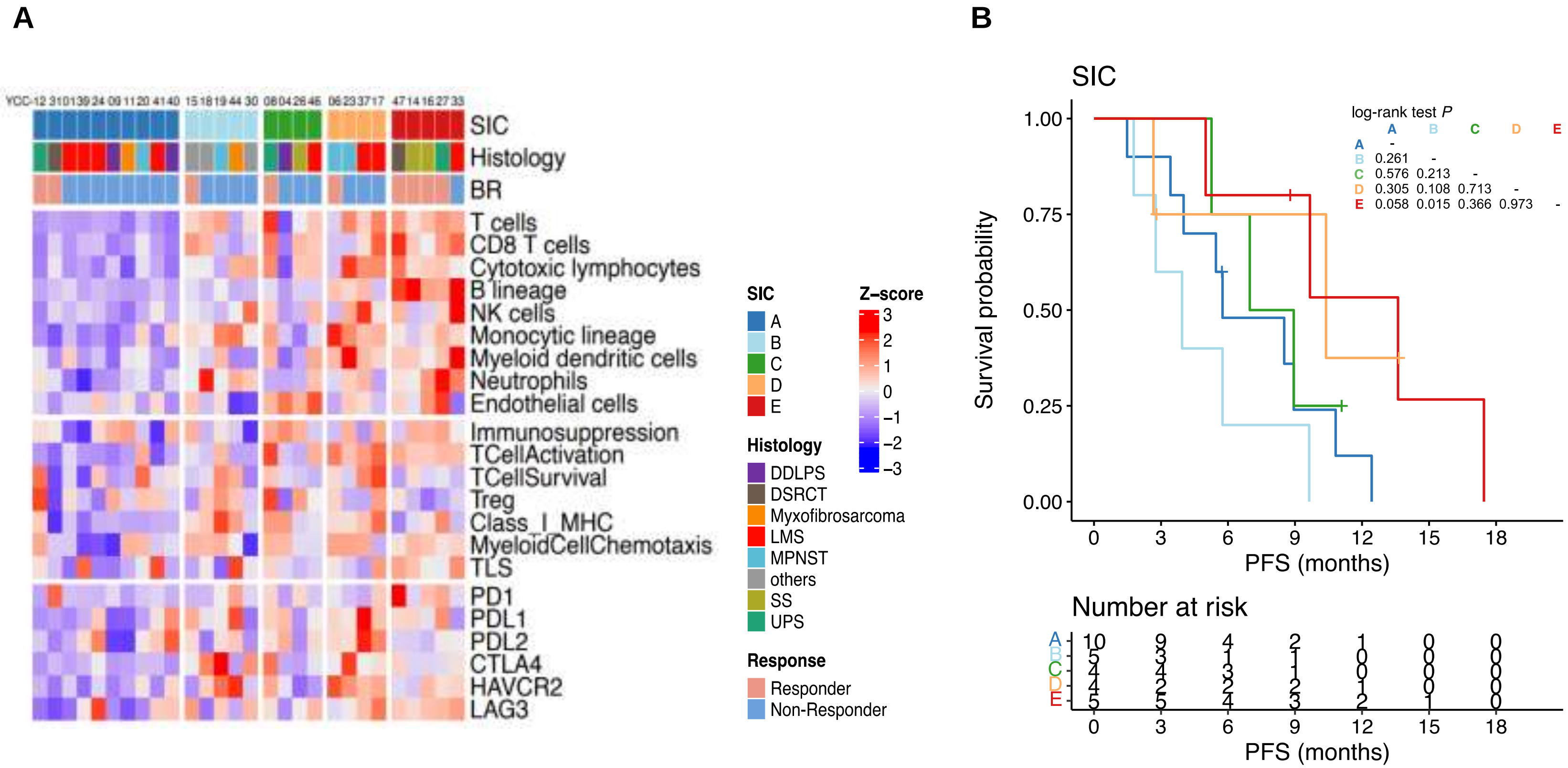

Supplementary Figure 6. Relationship between SIC, histology and treatment efficacy to durvalumab and pazopanib combination (n=28).

(A) Transcriptomic correlates of clinical response to durvalumab and pazopanib combination treatment with SIC. Heat maps describing tumour microenvironment cell infiltration. From top to bottom, heat maps indicate: MCP-counter scores of immune and stromal cells; single sample gene set enrichment analysis (ssGSEA) scores for immune-associated gene signatures; and gene expression levels for immune-checkpoint genes. The colour scale indicates Z-normalised values of each gene signature for gene expression across samples. The colour bar above the heatmap indicates responders (pink) and non-responders (blue) to the pazopanib-durvalumab combination.

(B) PFS of patients by the SIC. Source data are provided as a Source Data file.

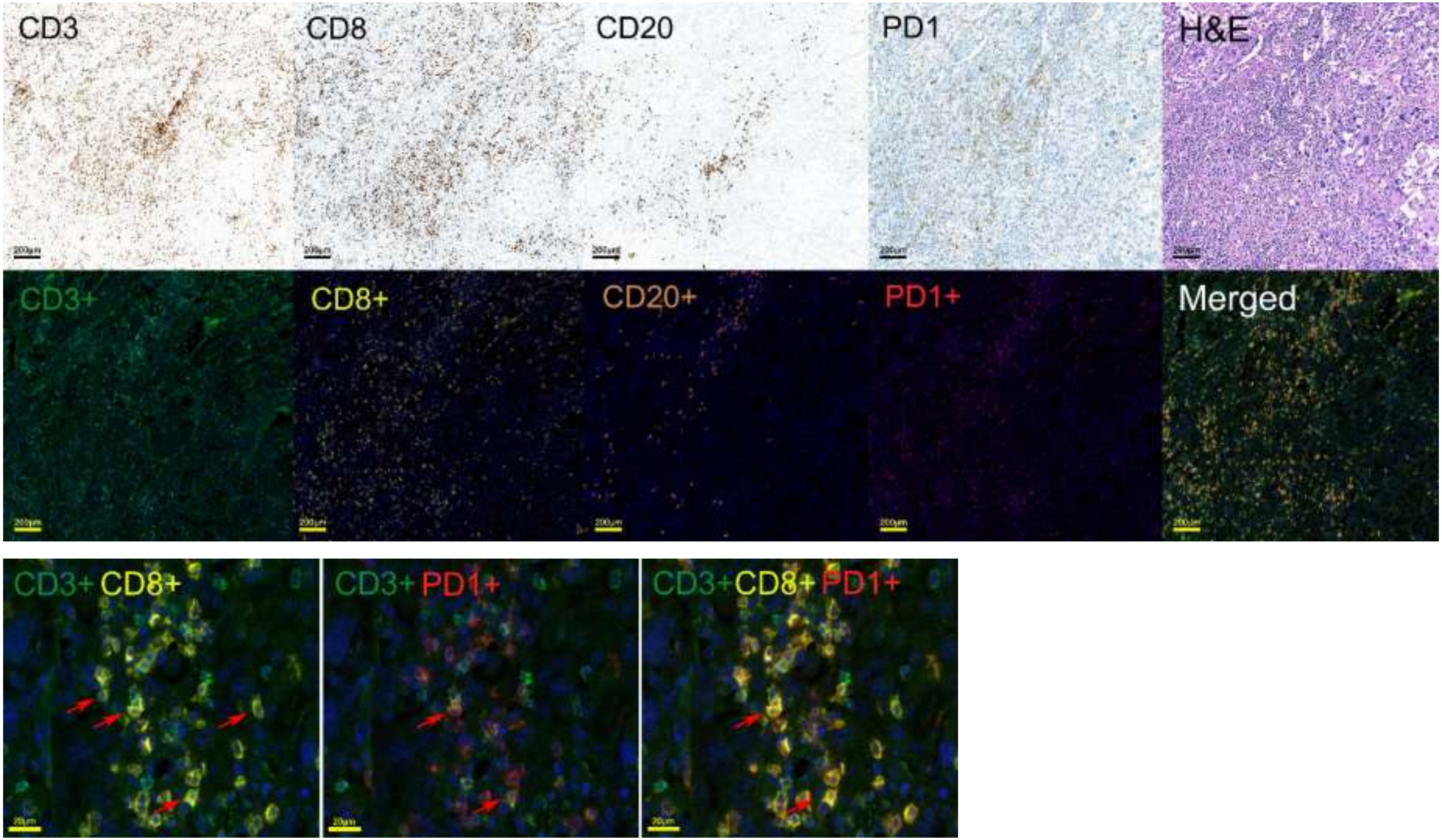

Supplementary Figure 7. Representative immunofluorescence staining (n=39).

(A) CD3 (green), CD8 (yellow), CD20 (orange), PD1 (red), and merged images.

(B) Red arrows indicates multispectral fluorescence images of double (CD3+CD8+ and CD3+PD1+) and triple positive cells (CD3+CD8+PD1+)

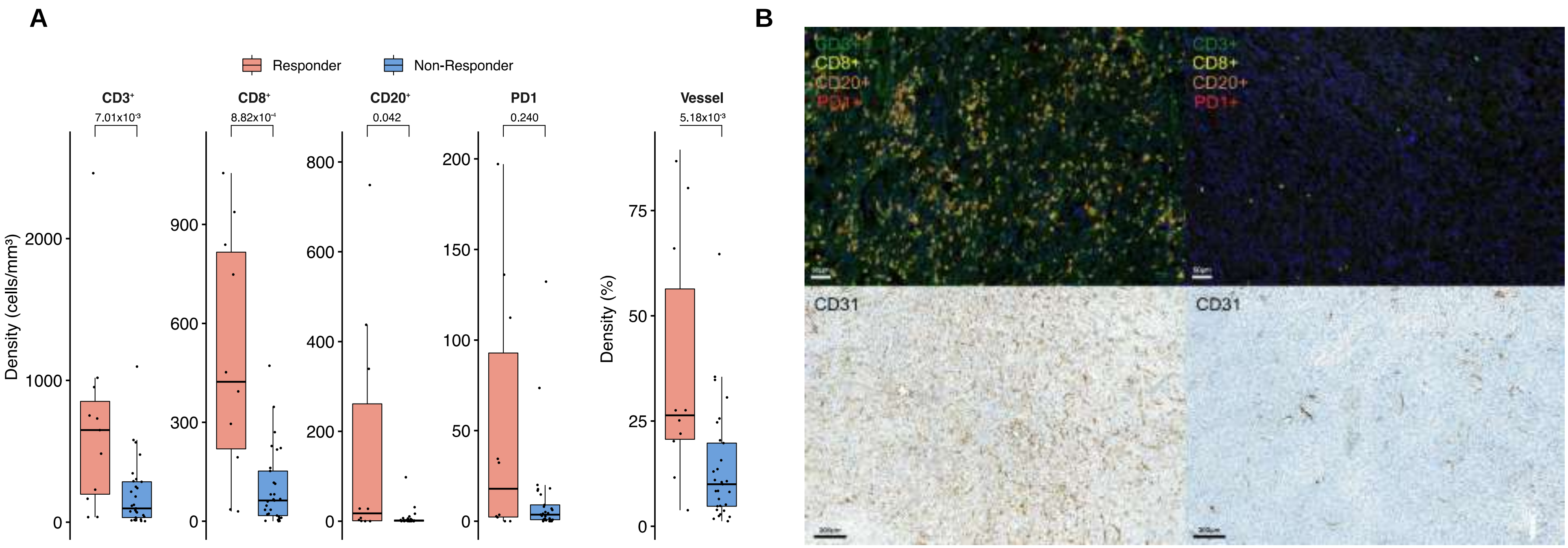

Supplementary Figure 8. Relationship between immune cell and vessel densities treatment efficacy to durvalumab and pazopanib combination (n=39).

(A) Density of CD3+ T, CD8+ T, CD20+ B, PD-1+ cells and vessel expression (CD31+) counts showing the differences between responders (n=10) and non-responders (n=29). P-values were calculated by two-sided Wilcoxon rank-sum tests. Centre lines, upper and lower bounds of boxplots indicate the median, 25th, and 75th quantile, respectively. The whiskers of boxplots indicate 1.5 times of the interquartile range. Source data are provided as a Source Data file.

(B) Representative images of indicated immunostaining (green for CD3+, yellow for CD8+, orange for CD20+ cells, red for PD-1+ and CD31+ vessel expression) and merged images for responders and non-responders

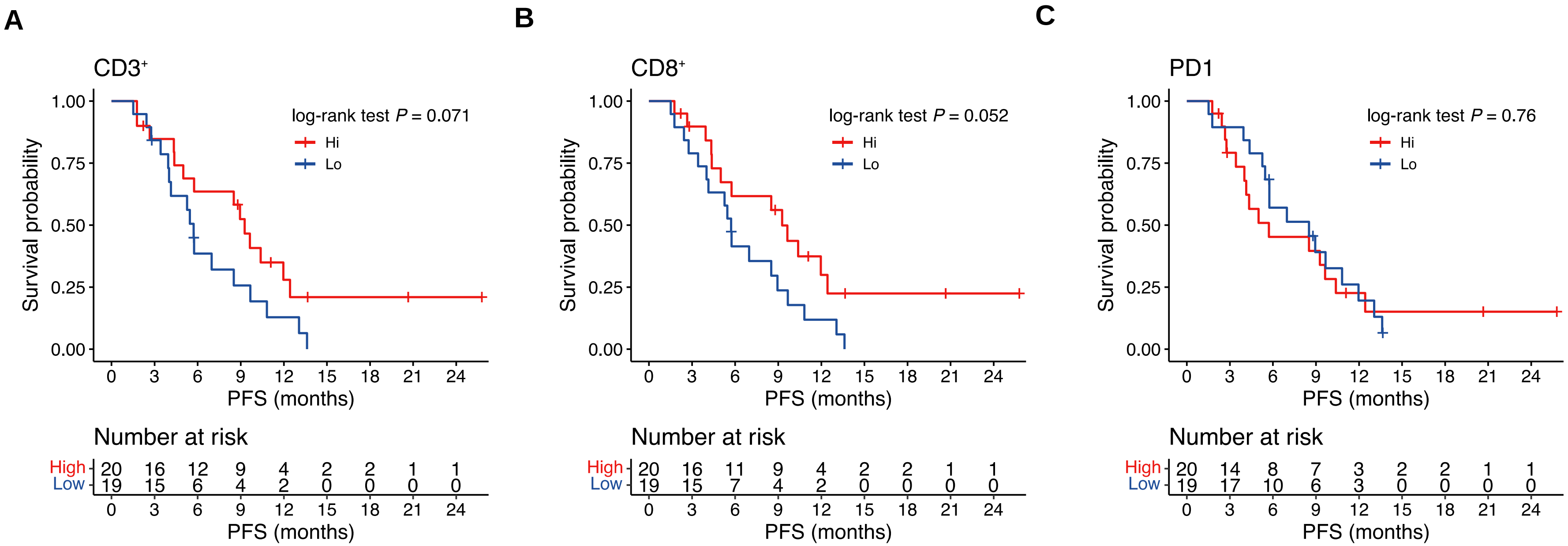

Supplementary Figure 9 . PFS of patients according to CD3+ T cell (A), CD8+ T cell (B), and PD1+ cell (C) infiltration (n=39). Hi indicates patients with density higher than or equal to the median, and Lo indicates patients with density lower than the median. Source data are provided as a Source Data file.

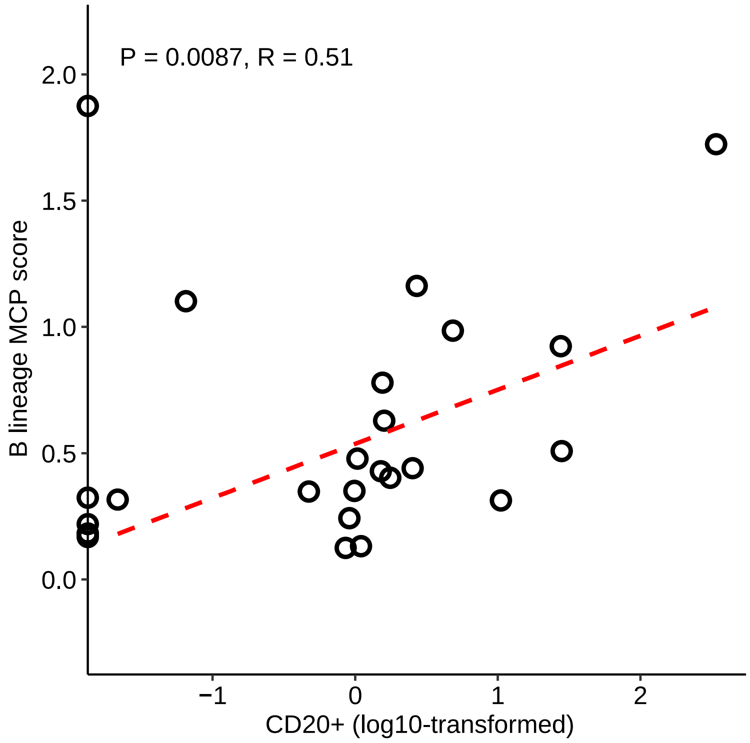

Supplementary Figure 10. Correlation plot between CD20+ density by IF and B lineage score from MCP-counter (n=25)

X-axis and y-axis indicate the CD20+ density (log10-transformed) and B lineage score from MCP-counter, respectively. Two-tailed Pearson correlation test was used to evaluate the association between raw CD20+ densities and B lineage scores. Source data are provided as a Source Data file.

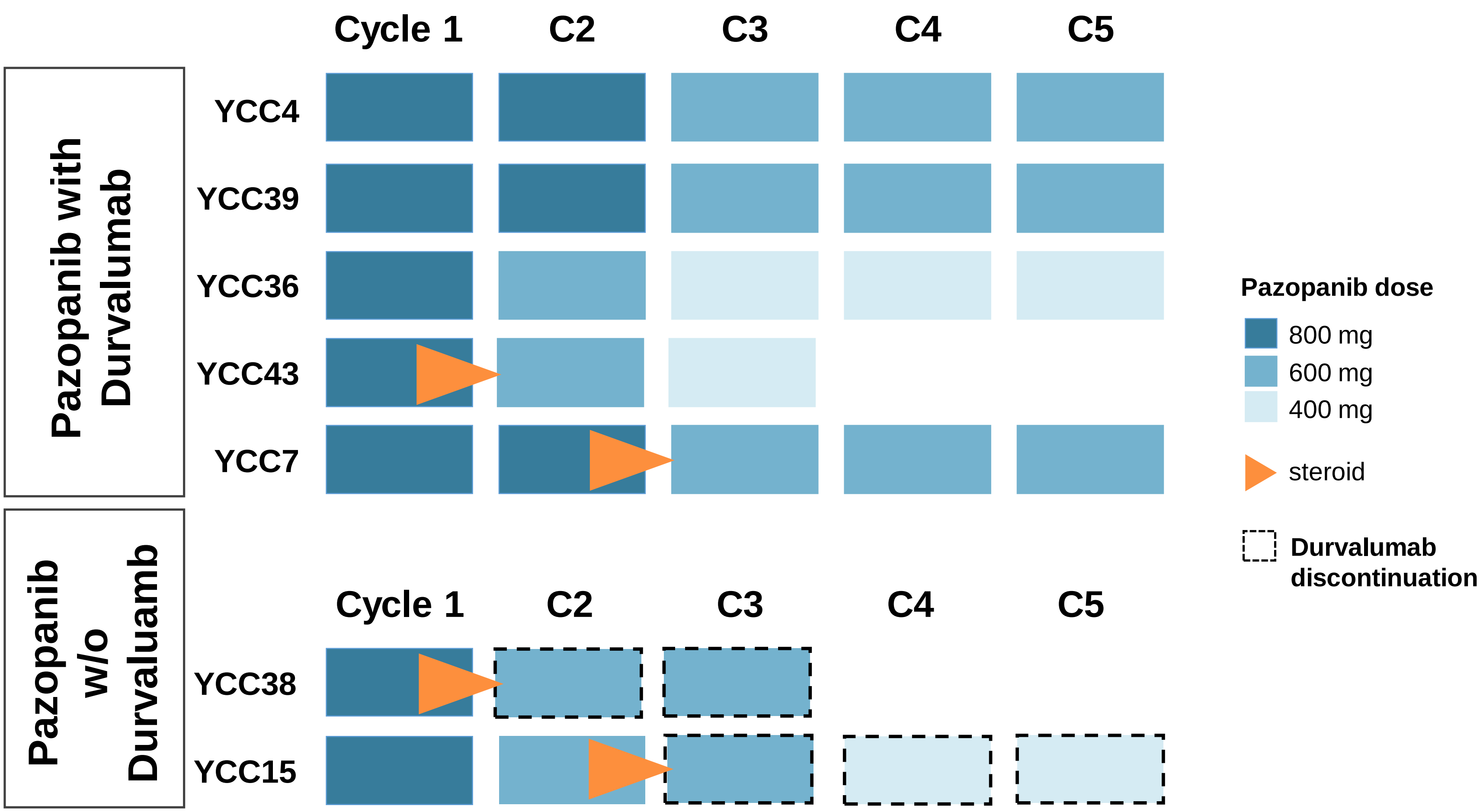

Supplementary Figure 11. Clinical course and management for patients had treatment-related grade 3-4 hepatotoxicity

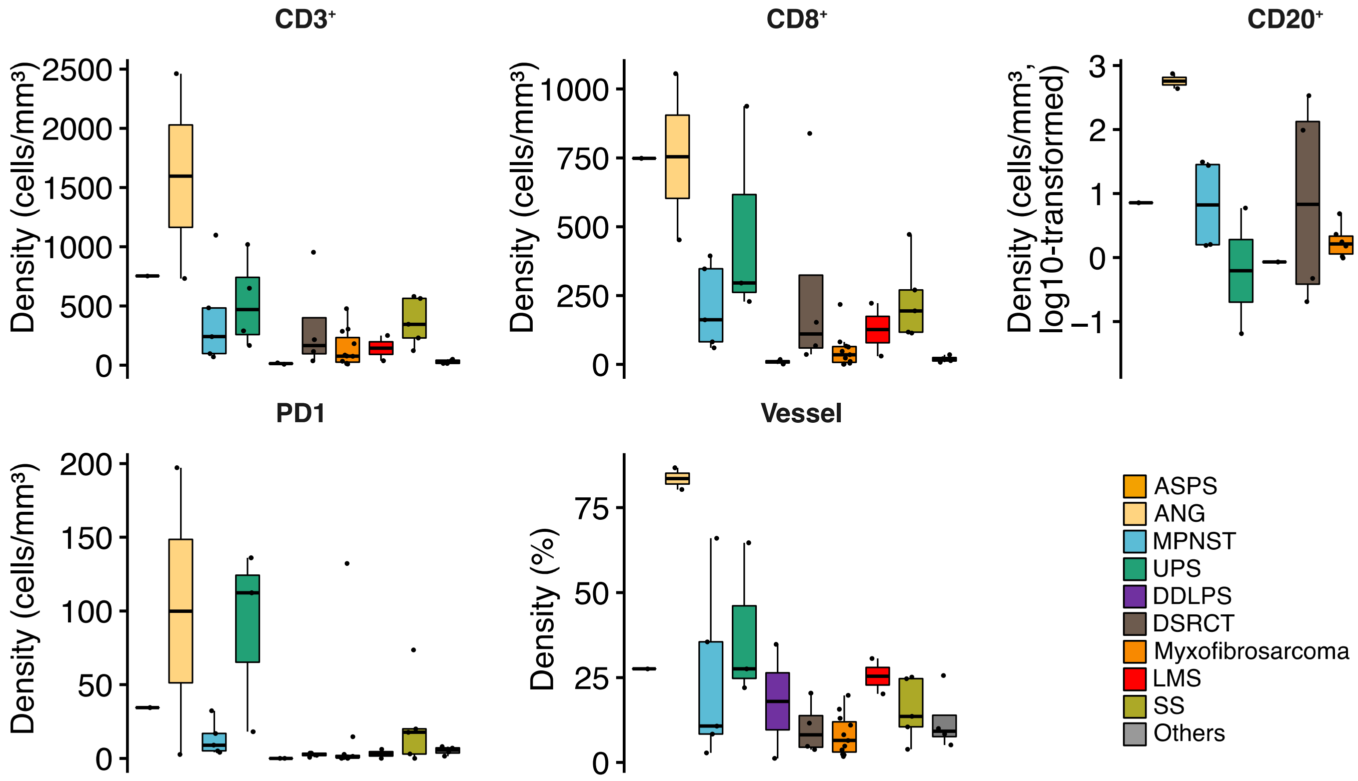

Supplementary Figure 12. Density of CD3+, CD8+, CD20+ cells and PD1+ and vessel expression according to different histologies (n=39).

Centre lines, upper and lower bounds of boxplots indicate the median, 25th, and 75th quantile, respectively. The whiskers of boxplots indicate 1.5 times of the interquartile range. ASPS; Alveolar soft part sarcoma (n=1), ANG; angiosarcoma (n=2), MPNST ; malignant peripheral nerve sheath tumour (n=5), UPS; undifferentiated pleomorphic sarcoma (n=3), DDLPS; dedifferentiated liposarcoma (n=2), DSRCT; desmoplastic small round cell tumour (n=4), Myxofibrosarcoma (n=4), LMS; leiomyosarcoma (n=11), SS; synovial sarcoma (n=2), and others (n=5). Source data are provided as a Source Data file.

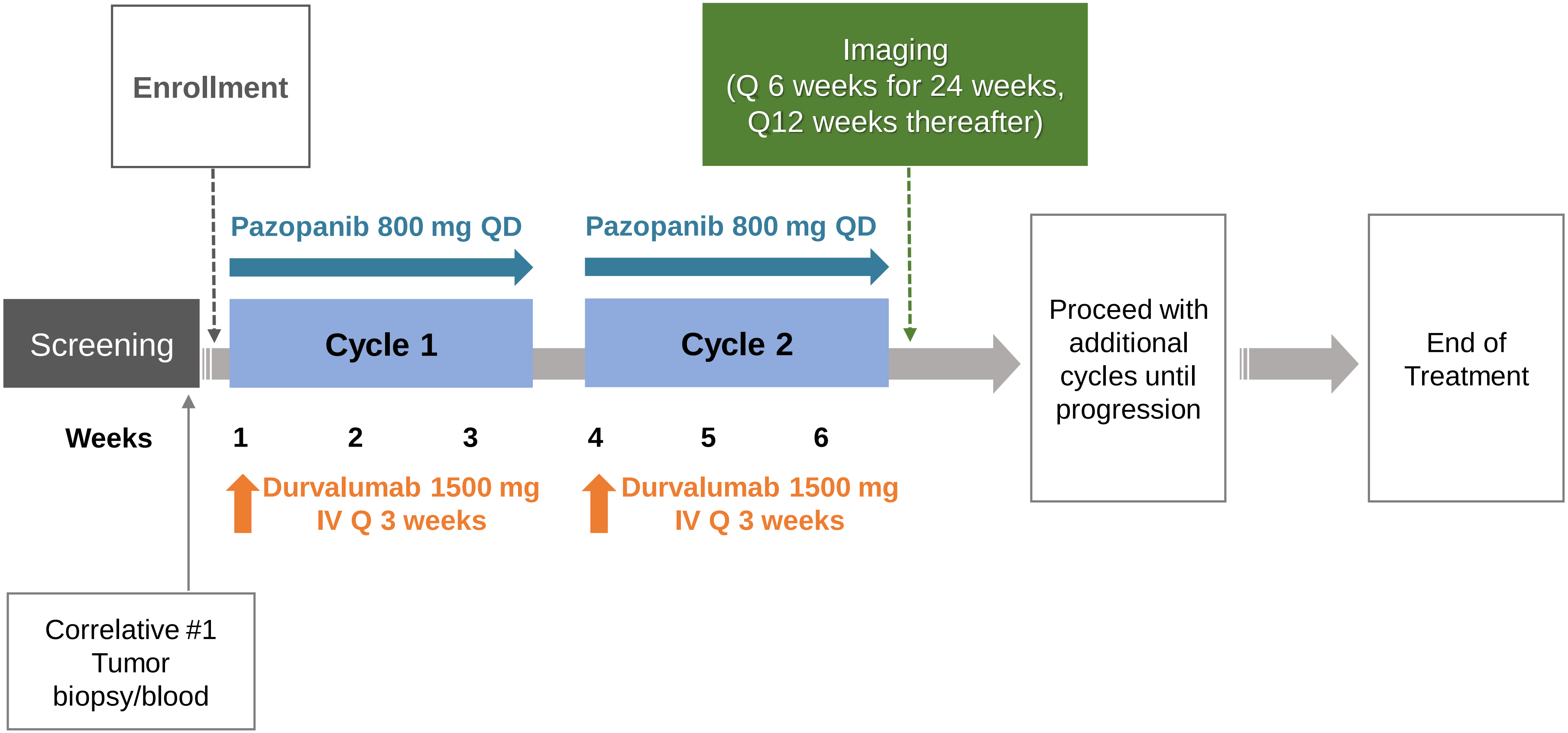

Supplementary Figure 13. Study schema.

All patients were required to meet screening eligibility criteria and sign informed consent prior to enrollment. All patients were initiated on pazopanib 800 mg orally daily continuously with 1500 mg of durvalumab intravenously every 3 weeks. Patients underwent radiographic imaging every 6 weeks. Correlative blood collection and tumor biopsy were done at the time of baseline.

Supplementary Table 1. Response rate of histologic subtypes (n=46)

	CR	PR	SD	PD	Total
**Leiomyosarcoma**	0	0	9 (75%)	3 (25%)	**12**
**Malignant peripheral nerve sheath tumor**	0	1 (20%)	4 (80%)	0	**5**
**Synovial sarcoma**	0	2 (50%)	2 (50%)	0	**4**
**Myxofibrosarcoma**	0	0	4 (100%)	0	**4**
**Desmoplastic small round cell tumor**	1 (25%)	1 (25%)	2 (50%)	0	**4**
**Undifferentiated pleomorphic sarcoma**	0	3 (75.0%)	1 (25.0%)	0	**4**
**Dedifferentiated liposarcoma**	0	0	2 (66.7%)	1 (33.3%)	**3**
**Clear cell sarcoma**	0	0	1 (50%)	1 (50%)	**2**
**Endometrial stromal sarcoma**	0	1 (50%)	1 (50%)	0	**2**
**Alveolar soft part sarcoma**	0	2 (100%)	0	0	**2**
**Angiosarcoma**	0	2 (100%)	0	0	**2**
**Others***	0	1 (50%)	1 (50%)	0	**2**

CR: complete response, PR: partial response, SD: stable disease, PD: progressive disease

*Others: hemangioendothelioma, malignant glomus tumor

Supplementary Table 2. Comparison of response assessment by immune-related Response Criteria (irRC) vs RECIST 1.1

		RECIST Best Response				
	Frequency	CR	PR	SD	PD	Total
irRC Best Response	irCR	1	0	0	0	1
	irPR	0	12	0	0	12
	irSD	0	1	27	1	29
	irPD	0	0	0	4	4
	Total	1	13	27	5	46

Supplementary Table 3. (A) Univariate and (B) Multivariate Cox regression analyses

(A)

Variable		HR	CI lower	CI upper	P
**Histology**	ASPS	0.00	0.00	Inf	1.00
	ANGIO	0.24	0.03	1.81	0.17
	DDLPS	3.55	1.03	12.24	0.05
	DSRCT	1.14	0.35	3.75	0.83
	LMS	1.55	0.74	3.25	0.25
	MPNST	1.12	0.34	3.70	0.85
	Myxofibrosarcoma	1.67	0.58	4.77	0.34
	SS	0.44	0.13	1.46	0.18
	UPS	1.15	0.40	3.28	0.79
	others	1.58	0.61	4.13	0.35
**Stage at diagnosis**	1, 2	-	-	-	-
	3, 4	1.67	0.82	3.40	0.16
**Primary site**	Trunk	-	-	-	-
	Extremity/head and neck	0.49	0.24	1.01	0.05
**Age**	< median	-	-	-	-
	>= median	1.14	0.59	2.20	0.70
**Sex**	Female	-	-	-	-
	Male	1.21	0.63	2.35	0.57
**SIC**	B cell (E)	0.37	0.11	1.30	0.12
	Desert (A)	1.61	0.67	3.84	0.29
	High (D)	0.50	0.12	2.18	0.36
	Low (B)	3.12	1.09	8.95	0.03
	Vascular (C)	0.87	0.26	2.99	0.83
**PD-L1**	Negative	-	-	-	-
	Positive	0.58	0.30	1.13	0.11
**PD1**	< median	-	-	-	-
	>= median	1.12	0.55	2.26	0.76
**CD3**	< median	-	-	-	-
	>= median	0.52	0.26	1.06	0.07
**CD8**	< median	-	-	-	-
	>= median	0.50	0.24	1.02	0.06
**CD20**	< median	-	-	-	-
	>= median	0.30	0.14	0.66	0.00
**Vessel**	< median	-	-	-	-
	>= median	0.44	0.21	0.91	0.03

(B) Multivariate Cox regression analyses

Variable		HR	CI_lower	CI_upper	P
**Histology**	DDLPS	1.07	0.24	4.81	0.93
**CD20**	< median	-	-	-	-
	>= median	0.35	0.16	0.80	0.01
**Vessel**	< median	-	-	-	-
	>= median	0.57	0.27	1.22	0.15

HR: hazard ratio, CI: confidence interval

Univariate and multivariate statistical values were calculated by the Cox regression model.

Supplementary References

1. Li, H. & Durbin, R. Fast and accurate short read alignment with Burrows-Wheeler transform. *Bioinformatics* **25**, 1754-1760 (2009).
2. Li, H. *et al.* The Sequence Alignment/Map format and SAMtools. *Bioinformatics* **25**, 2078-2079 (2009).
3. McKenna, A. *et al.* The Genome Analysis Toolkit: a MapReduce framework for analyzing next-generation DNA sequencing data. *Genome Res* **20**, 1297-1303 (2010).
4. Karczewski, K. J. *et al.* Variation across 141,456 human exomes and genomes reveals the spectrum of loss-of-function intolerance across human protein-coding genes. *BioRxiv*, 531210 (2019).
5. McLaren, W. *et al.* The Ensembl Variant Effect Predictor. *Genome Biol* **17**, 122 (2016).
6. Shukla, S. A. *et al.* Comprehensive analysis of cancer-associated somatic mutations in class I HLA genes. *Nat Biotechnol* **33**, 1152-1158 (2015).
7. Hundal, J. *et al.* pVAC-Seq: A genome-guided in silico approach to identifying tumor neoantigens. *Genome Med* **8**, 11 (2016).
8. Jurtz, V. *et al.* NetMHCpan-4.0: Improved Peptide-MHC Class I Interaction Predictions Integrating Eluted Ligand and Peptide Binding Affinity Data. *J Immunol* **199**, 3360-3368 (2017).
9. McGranahan, N. *et al.* Allele-Specific HLA Loss and Immune Escape in Lung Cancer Evolution. *Cell* **171**, 1259-1271 e1211 (2017).
10. Talevich, E., Shain, A. H., Botton, T. & Bastian, B. C. CNVkit: Genome-Wide Copy Number Detection and Visualization from Targeted DNA Sequencing. *PLoS Comput Biol* **12**, e1004873 (2016).
11. Zack, T. I. *et al.* Pan-cancer patterns of somatic copy number alteration. *Nat Genet* **45**,

1134-1140 (2013).

12. Carter, S. L. *et al.* Absolute quantification of somatic DNA alterations in human cancer. *Nat Biotechnol* **30**, 413-421 (2012).
13. Mermel, C. H. *et al.* GISTIC2.0 facilitates sensitive and confident localization of the targets of focal somatic copy-number alteration in human cancers. *Genome Biol* **12**, R41 (2011).
14. Dobin, A. *et al.* STAR: ultrafast universal RNA-seq aligner. *Bioinformatics* **29**, 15-21 (2013).
15. Wang, L., Feng, Z., Wang, X., Wang, X. & Zhang, X. DEGseq: an R package for identifying differentially expressed genes from RNA-seq data. *Bioinformatics* **26**, 136-138 (2010).
16. Becht, E. *et al.* Estimating the population abundance of tissue-infiltrating immune and stromal cell populations using gene expression. *Genome Biol* **17**, 218 (2016).
17. Petitprez, F. *et al.* B cells are associated with survival and immunotherapy response in sarcoma. *Nature* **577**, 556-560 (2020).
18. Hanzelmann, S., Castelo, R. & Guinney, J. GSVA: gene set variation analysis for microarray and RNA-seq data. *BMC Bioinformatics* **14**, 7 (2013).
19. Haas, B. J. *et al.* Accuracy assessment of fusion transcript detection via read-mapping and de novo fusion transcript assembly-based methods. *Genome Biol* **20**, 213 (2019).
20. Trapnell, C. *et al.* Differential gene and transcript expression analysis of RNA-seq experiments with TopHat and Cufflinks. *Nat Protoc* **7**, 562-578 (2012).

Supplementary Note

Investigational Drug Substance(s)	Durvalumab (MEDI4736)<br>Pazopanib
Study Number	ESR-17-13151
Version Number	2.1
Date	01.July. 2021

A Phase II trial of Pazopanib and Durvalumab (MEDI4736) for Metastatic Soft Tissue Sarcoma

PROTOCOL SYNOPSIS

**Study Title: A Phase II trial of Pazopanib and Durvalumab for Metastatic Soft Tissue Sarcoma**
**Protocol Number: ESR-17-13151**
**Clinical Phase: II**
**Study Duration: 30 months**
**Investigational Product(s) and Reference Therapy:**<br>Durvalumab (MEDI4736) will be supplied in glass vials containing 500 mg of liquid solution at a concentration of 50 mg/mL for intravenous (IV) administration.<br>Pazopanib will be supplied as 400 mg or 200 mg tablets
**Research Hypothesis**<br>The yearly incidence of soft-tissue sarcomas (STS) in the USA is roughly 11,280 cases, and metastatic STS have a median overall survival of about 12 months. After the standard 1st line chemotherapy with doxorubicin, the only a few treatment option is available. Recent pivotal phase III trial which compared the placebo versus pazopanib in refractory sarcoma patients (the PALETTE trial) has demonstrated a significantly prolonged median PFS[1]. With this trial, pazopanib become a standard treatment for 2nd line treatment in STS. However, the median PFS and the overall response rate for pazopanib still remains 4.6 months and 6%, respectively. In our retrospective study with pazopanib in 43 Asian patients, similar survival outcome (median 4.3 months PFS) was noticed with manageable toxicity. Therefore, further combination strategy to improve survival outcome is strongly warranted for STS.<br>Pazopanib is an angiogenesis inhibitor targeting VEGFR-1, -2, and -3; PDGFR-α and -β; and the receptor c-Kit, and is indicated for the treatment of subjects with advanced renal cell carcinoma (RCC) and advanced STS. Pro-angiogenic factors suppress various immune functions whereas antiangiogenic agents have potential to modulate the tumor microenvironment and improve immunotherapy. An analysis of patients with RCC treated with pazopanib demonstrated that elevated expression of PD-L1 correlates with shorter PFS. We also identified 43% of PD-L1 expression in STS and PD-L1 expression had worse overall survival (5-year survival rate: 48% in PD-L1 positive vs. 68% in PD-L1 negative, p=0.015). In detail, PD-L1 expression was reported 52.6% in synovial sarcoma, 37.6% in rhabdomyosarcoma, and 100% in epithelioid sarcoma. For this orphan tumor, STS, PD-L1 targeting may be a promising strategy and favorable toxicity may warrant further combination.
**Objectives:**

**Primary Objectives:**<br>To evaluate antitumor efficacy of durvalumab and pazopanib combination<br>: Overall Response Rate (ORR) will be based on RECIST version 1.1<br><br>**Secondary Objective(s)**:<br>1) To evaluate antitumor efficacy of durvalumab and pazopanib combination<br>• Progression-free survival (PFS)<br>• Overall survival (OS)<br>• Disease control rate (DCR)<br>• Immune-Related Response Criteria (irRC)<br>2) To evaluate safety of durvalumab and pazopanib combination<br>• Adverse events<br><br>**Exploratory Objective(s):**<br>Biomarker exploration for predictive biomarker<br>• Whole Exome Sequencing and RNA sequencing<br>• PD-L1 immunohistochemistry and OPAL multiplex analyses
**Study Design:**<br><br>This is a multi-center, and phase II study to evaluate the clinical activity of durvalumab in combination with pazopanib

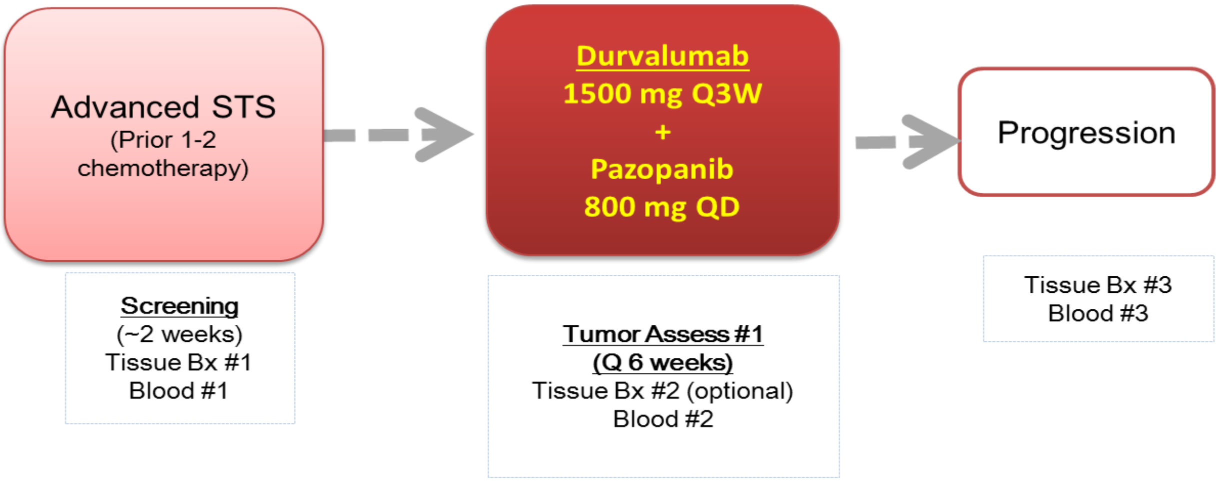

**Number of Patients: 46**

Study Population: metastatic/recurred soft tissue sarcoma

Inclusion Criteria:

1. Histologically confirmed STS progression to 1 or 2 prior chemotherapy

   : Exclude pazopanib-resistant subtype - embryonal rhabdomyosarcoma, chondrosarcoma, osteosarcoma, Ewing tumours, primitive neuroectodermal tumour, gastrointestinal stromal tumour, dermatofibrosarcoma protuberans, inflammatory myofibroblastic sarcoma, and liposarcoma

2. Age ≥ 19 years at time of study entry.

3. Eastern Cooperative Oncology Group (ECOG) performance status of 0 or 1

4. Measurable disease by Response Evaluation Criteria in Solid Tumors Version 1.1

5. Body weight >30kg

6. Adequate laboratory findings

7. Evidence of post-menopausal status or negative urinary or serum pregnancy test for female pre-menopausal patients.

8. Patient is willing and able to comply with the protocol for the duration of the study

9. Must have a life expectancy of at least 12 weeks

10. Capable of giving signed informed consent which includes compliance with the requirements and restrictions listed in the informed consent form (ICF) and in this protocol.

11. Patients with evidence of portal hypertension (including splenomegaly detected radiographically) or any prior history of variceal bleeding must have had endoscopic evaluation within the 3 months immediately prior to enrolment, and the findings do not represent a high bleeding risk.

Exclusion Criteria:

1. More than 4 prior cytotoxic regimens

2. Participation in another clinical study with an investigational product during the last 2 weeks

   :observational (non-interventional) clinical study or during the follow-up period of an interventional study is permitted

3. Receipt of the last dose of anticancer therapy 14 days prior to the first dose of study drug

4. Any previous treatment with a PD1 or PD-L1 inhibitor (including durvalumab) and/or pazopanib

5. Mean QT interval corrected for heart rate (QTc) >480 ms calculated from 3 electrocardiograms (ECGs) using Fridericia's Correction

6. Any unresolved toxicity NCI CTCAE Grade ≥2 from previous anticancer therapy with the exception of alopecia, vitiligo, and the laboratory values defined in the inclusion criteria

7. Any concurrent chemotherapy, biologic, or hormonal therapy for cancer treatment within 2 weeks prior to entering the study. Concurrent use of hormonal therapy for non-cancer-related conditions (e.g., hormone replacement therapy) is acceptable

8. Radiotherapy treatment to more than 30% of the bone marrow or with a wide field of radiation within 4 weeks of the first dose of study drug

9. Major surgical procedure (as defined by the Investigator) within 28 days prior to the first dose of IP.

10. History of allogenic organ transplantation.

11. Active or prior documented autoimmune or inflammatory disorders (including inflammatory bowel disease [e.g., colitis or Crohn's disease], diverticulitis [with the exception of diverticulosis], systemic lupus erythematosus, Sarcoidosis syndrome, or Wegener syndrome [granulomatosis with polyangiitis, Graves' disease, rheumatoid arthritis, hypophysitis, uveitis, etc]).

12. Uncontrolled intercurrent illness, including but not limited to, ongoing or active infection, symptomatic congestive heart failure, uncontrolled hypertension, unstable angina pectoris, cardiac arrhythmia, interstitial lung disease, serious chronic gastrointestinal conditions associated with diarrhea, or psychiatric illness/social situations that would limit compliance with study requirement, substantially increase risk of incurring AEs or compromise the ability of the patient to give written informed consent

13. History of active infection

14. History of another primary malignancy

15. History of leptomeningeal carcinomatosis who are neurologically unstable or have required active treatment

16. Receipt of live attenuated vaccine within 30 days prior to the first dose of IP.

17. Female patients who are pregnant or breastfeeding or male or female patients of reproductive potential who are not willing to employ effective birth control from screening to 90 days after the last dose.

18. Known allergy or hypersensitivity to any of the study drugs or any of the study drug excipients.

19. History of any of the following in the past 6 months: cardiac angioplasty or stenting, myocardial infarction, unstable angina, coronary artery bypass graft surgery, symptomatic peripheral vascular disease class III or IV congestive heart failure, as defined by the New York Heart Association), thromboembolic events

Investigational Product(s), Dose and Mode of Administration:

Drug	Dose/ Potency	Frequency	Treatment Period (Q3 weeks)	Route of Administration
**Durvalumab**	1500 mg	Q3W	Day 1	IV infusion
**Pazopanib**	800 mg	QD	Day 1-21	p.o.

Study Assessments and Criteria for Evaluation:

Safety Assessments:

All toxicities will be graded according to NCI CTCAE, Version 4.03.

Efficacy Assessments

The efficacy analysis will be conducted by programmatically deriving each efficacy endpoint based on RECIST 1.1 criteria

Statistical Methods and Data Analysis:

Descriptive statistics will be presented. Continuous variables will be summarized by the number of observations, mean, standard deviation, median, minimum, and maximum. Categorical variables will be summarized by frequency counts and percentages for each category. Unless otherwise stated, percentages will be calculated out of the population total for the corresponding treatment. All data collected will be listed. Results of all statistical analysis will be presented using a 95% confidence interval

Sample Size Determination:

On the basis of a prospective study with previously treated metastatic STS (Lancet Oncol 2018; 19: 416–26), five (13%) of these 40 evaluable patients achieving a confirmed response would be considered sufficient evidence of promising clinical activity in this setting

Therefore, similar with previous studies (Lancet Oncol 2018; 19: 416–26 and Lancet Oncol 2017) with immune checkpoint inhibitor for soft tissue sarcoma, this study designed to yielded 90% power to detect

clinical activity if at least 20% of patients had a confirmed response at 0·05 level of significance (one-sided test), and clinical inactivity if 5% or fewer patients achieved a confirmed response.

Applying optimal design, a total of 41 evaluable patients per cohort had to be accrued. After the enrollment of first stage (n=22), interim analysis will be done to decide whether to continue the study or not. If 2 or more responses are observed in the first 22 patients, accrual will be continued until a total of 41 evaluable patients reached.

If, of these 41 patients, 5 or more responses warranted additional investigation. Allowing for a follow-up loss rate of 10 %, the total sample size is expected as 46 patients.

TABLE OF CONTENTS **PAGE**

PROTOCOL SYNOPSIS 2
TABLE OF CONTENTS 8
ABBREVIATIONS AND DEFINITION OF TERMS 13
1. INTRODUCTION 17
1.1 Disease background 17
1.2 Study Rationale 17
1.2.1 Immunotherapies 17
1.2.2 Durvalumab background/non-clinical and clinical experience 18
1.3 Rationale for conducting this study 19
1.3.1 Rationale for the immunotherapies for STS 19
1.3.2 Rationale for the combination treatment 19
1.3.3 Rationale for dose 20
1.4 Benefit/risk and ethical assessment 22
1.4.1 Durvalumab 22
1.4.2 Pazopanib 23
1.4.3 Overall risk benefit 23
2. PROVIDE SUMMARY OF OVERALL BENEFIT-RISK FOR THE STUDY OBJECTIVES 24
2.1 Primary objective(s) 24
2.2 Secondary objective(s) 24
2.3 Exploratory objective(s) 24
3. STUDY DESIGN 25
3.1 Overview of study design 25
3.2 Study schema 25
4. PATIENT SELECTION 26
4.1 Inclusion criteria 26
4.2 Exclusion criteria 28
4.3 Withdrawal of patients from study treatment and/or study 30

5. INVESTIGATIONAL PRODUCT(S) ... 33
5.1 Durvalumab ... 33
5.1.1 Formulation/packaging/storage ... 33
5.1.2 Study drug preparation ... 33
5.1.3 Monitoring of dose administration ... 34
5.2 Pazopanib ... 34
5.2.1 Formulation/packaging/storage ... 34
5.2.2 Doses, treatment regimens, and administration ... 34
5.3 Accountability and disposition of IP ... 35
6. TREATMENT PLAN ... 36
6.1 Patient enrollment ... 36
6.2 Dosage and administration ... 36
6.3 Toxicity management guidelines ... 37
6.3.1 Dose selection ... 37
6.3.2 Durvalumab ... 37
6.3.3 Pazopanib ... 38
7. RESTRICTIONS DURING THE STUDY AND CONCOMITANT TREATMENT(S) ... 44
7.1 Restrictions during the study ... 44
7.2 Concomitant treatment(s) ... 45
7.2.1 Permitted concomitant medications ... 45
7.2.2 Excluded concomitant medications ... 46
STUDY PROCEDURES ... 48
8. STUDY PROCEDURES…………………………………………...………48
8.1 Schedule of study procedures ... 48
8.1.1 Screening phase ... 48
8.1.2 Treatment phase ... 49
8.1.3 End of treatment ... 49
8.2 Description of study procedures ... 49
8.2.1 Medical history and physical examination, electrocardiogram, weight, and vital signs ... 49
8.2.2 Clinical laboratory tests ... 50

8.3 Biological sampling procedures ... 52
8.3.1 Biomarker/pharmacodynamic sampling and evaluation methods ... 52
9. DISEASE EVALUATION AND METHODS ... 54
9.1. Evaluation of Target Lesions ... 54
9.2 Evaluation of Non-Target Lesions ... 55
9.3 Evaluation of New Lesions ... 55
9.4 Duration of Response ... 58
9.5 Definition of Overall Response Using irRC ... 58
10. ASSESSMENT OF SAFETY ... 60
10.1.1 Safety parameters ... 60
10.1.2 Definition of serious adverse events ... 60
10.1.3 Definition of adverse events of special interest (AESI) ... 60
10.2 Assessment of safety parameters ... 64
10.2.1 Assessment of severity ... 64
10.2.2 Assessment of relationship ... 64
10.3 Recording of adverse events and serious adverse events ... 65
10.3.1 Study recording period and follow-up for adverse events and serious adverse events ... 66
10.3.2 Causality collection ... 67
10.3.3 Adverse events based on signs and symptoms ... 67
10.3.4 Adverse events based on examinations and tests ... 67
10.3.5 Hy’s Law ... 68
10.3.6 Disease progression ... 68
10.3.7 Deaths ... 68
10.3.8 Reporting of serious adverse events ... 69
10.3.9 Reporting of deaths to AstraZeneca ... 70
10.3.10 Other events requiring reporting ... 70
10.3.11 Overdose ... 71
10.3.12 Hepatic function abnormality ... 71
10.3.13 Pregnancy or maternal exposure ... 71
10.3.14 Paternal exposure ... 72
10.4 Medication error ... 72

11. STATISTICAL METHODS AND SAMPLE SIZE DETERMINATION 74
11.1 Description of analysis sets 74
11.3 Methods of statistical analyses 75
12. ETHICAL AND REGULATORY REQUIREMENTS 75
12.1 Ethical conduct of the study 75
12.2 Ethics and regulatory review 75
12.3 Informed consent 75
12.4 Audits and inspections and protocol amendment 75
13. STUDY MANAGEMENT 76
13.1 Monitoring of the study 76
14. DATA MANAGEMENT 76

LIST OF APPENDICES

Appendix 1. Dosing Modification and Toxicity Management Guidelines for Immune-Mediated, Infusion- Related, and Non-Immune–Mediated Reactions
Appendix 2. Study Flow Chart

ABBREVIATIONS AND DEFINITION OF TERMS

The following abbreviations and special terms are used in this study Clinical Study Protocol.

Abbreviation or special term	Explanation
ADA	Anti-drug antibody
ADCC	Antibody-dependent cell-mediated cytotoxicity
AE	Adverse event
AESI	Adverse event of special interest
ALP	Alkaline phosphatase
ALT	Alanine aminotransferase
APC	Antigen-presenting cells
AST	Aspartate aminotransferase
AUC	Area under the concentration-time curve
CDC	Complement-dependent cytotoxicity
CI	Confidence interval
CL	Clearance
C_{max}	Peak concentration
$C_{max,ss}$	Peak concentration at steady state
C_{min}	Trough concentration
$C_{min,ss}$	Trough concentration at steady state
CNS	Central nervous system
CR	Complete response
CT	Computed tomography
CTLA-4	Cytotoxic T-lymphocyte-associated antigen-4
DC	Disease control
DCR	Disease control rate
DLT	Dose-limiting toxicity
DNA	Deoxyribonucleic acid

Abbreviation or special term	Explanation
DoR	Duration of response
ECG	Electrocardiogram
ECOG	Eastern Cooperative Oncology Group
EDTA	Disodium edetate dihydrate
Fc	Fragment crystallizable
FFPE	Formalin fixed paraffin embedded
FSH	Follicle-stimulating hormone
FTIH	First-time-in-human
GCP	Good Clinical Practice
GMP	Good Manufacturing Practice
GLP	Good Laboratory Practice
HCl	Hydrochloride
HIV	Human immunodeficiency virus
ICF	Informed consent form
ICH	International Conference on Harmonization
IEC	Independent Ethics Committee
IFN	Interferon
IGF	Insulin-like growth factor
IgG1	Immunoglobulin G1
IgG2	Immunoglobulin G2
IGSF	Immunoglobulin superfamily
IHC	Immunohistochemistry
IL	Interleukin
irAE	Immune-related adverse event
IRB	Institutional Review Board
IV	Intravenous(ly)
MAb	Monoclonal antibody

**Abbreviation or special term**	**Explanation**
MDSC	Myeloid-derived suppressor cells
MedDRA	Medical Dictionary for Regulatory Activities
miRNA	Micro ribonucleic acid
MRI	Magnetic resonance imaging
mRNA	Messenger ribonucleic acid
MTD	Maximum tolerated dose
NCI CTCAE	National Cancer Institute Common Terminology Criteria for Adverse Events
NK	Natural killer
NOAEL	No-observed-adverse-effect level
NSCLC	Non-small cell lung cancer
OR	Objective response
ORR	Objective response rate
OS	Overall survival
PBMC	Peripheral blood mononuclear cell
PD	Progressive disease
PD-1	Programmed cell death 1
PD-L1	Programmed cell death ligand 1
PD-L2	Programmed cell death ligand 2
PFS	Progression-free survival
PK	Pharmacokinetic(s)
PR	Partial response
PRO	Patient-reported outcome
PVC	Polyvinyl chloride
Q2W	Every 2 weeks
Q3M	Every 3 months
Q3W	Every 3 weeks

**Abbreviation or special term**	**Explanation**
Q4W	Every 4 weeks
Q12W	Every 12 weeks
QoL	Quality of life
QTc	Time between the start of the Q wave and the end of the T wave corrected for heart rate
QTcF	QT interval on ECG corrected using the Frederica's formula
RCC	Renal cell carcinoma
RECIST	Response Evaluation Criteria in Solid Tumors
RNA	Ribonucleic acid
SAE	Serious adverse event
SD	Stable disease
SID	Subject identification
sPD-L1	Soluble programmed cell death ligand 1
SOCS3	Suppressor of cytokine signaling 3
SUSAR	Suspected unexpected serious adverse reaction
$t_{½}$	Half life
TEAE	Treatment-emergent adverse event
TIL	Tumor infiltrating lymphocyte
T_{max}	Time to peak concentration
$T_{max,ss}$	Time to peak concentration at steady state
TNF-α	Tumor necrosis factor alpha
TSH	Thyroid stimulating hormone
ULN	Upper limit of normal
USA	United States of America
WFI	Water for injection
WHO	World Health Organization

1. INTRODUCTION

1.1 Disease background

Soft tissue sarcoma (STS) is a rare group of heterogeneous mesenchymal cancers originating from connective tissue. There are more than 50 histological subtypes of STS. At present all these subtypes are usually grouped under the heading of STS for the purpose of treatment, although an increasing number of new treatment options are expected to be directed more specifically at individual histological subtypes. The yearly incidence of STS in the USA is roughly 11,280 cases. The 5-year survival in Europe for adult STS (excluding visceral STS) averages 60%, and metastatic STS have a median overall survival of about 12 months[3].

After the standard 1st line chemotherapy with doxorubicin, the only a few treatment option is available. Recent pivotal phase III trial which compared the placebo versus pazopanib in refractory sarcoma patients (the PALETTE trial) has demonstrated a significantly prolonged median PFS[1]. Pazopanib is an angiogenesis inhibitor targeting VEGFR-1, -2, and -3; PDGFR-α and -β; and the receptor c-Kit, and is indicated for the treatment of subjects with advanced renal cell carcinoma (RCC) and advanced STS. With this trial, pazopanib become a standard treatment for 2nd line treatment in STS. However, the median PFS and the overall response rate for pazopanib still remains 4.6 months and 6%, respectively. In our retrospective study with pazopanib in 43 Asian patients, similar survival outcome (median 4.3 months PFS) was noticed with manageable toxicity [4]. Therefore, further combination strategy to improve survival outcome is strongly warranted for STS.

1.2 Study Rationale

1.2.1 Immunotherapies

It is increasingly understood that cancers are recognized by the immune system, and, under some circumstances, the immune system may control or even eliminate tumors. PD-L1 is part of a complex system of receptors and ligands that are involved in controlling T-cell activation. The PD-1 receptor (CD279) is expressed on the surface of activated T cells [5]. It has 2 known ligands: PD-L1 (B7-H1; CD274) and PD-L2 (B7-DC; CD273) [6]. The PD-1 and PD-L1/PD-L2 belong to the family of immune checkpoint proteins that act as co-inhibitory factors, which can halt or limit the development of T cell response. When PD-L1 binds to PD-1, an inhibitory signal is transmitted into the T cell, which reduces cytokine production and suppresses T-cell proliferation. Tumor cells exploit this immune checkpoint pathway as a mechanism to evade detection and inhibit immune response.

PD-L1 is constitutively expressed by B-cells, dendritic cells, and macrophages. Importantly, PD-L1 is commonly over-expressed on tumor cells or on non-transformed cells in the tumor microenvironment [7]. PD-L1 expressed on the tumor cells binds to PD-1 receptors on the activated T-cells leading to the inhibition of cytotoxic T cells. These deactivated T cells remain inhibited in the tumor microenvironment. The PD-1/PD-L1 pathway represents an adaptive immune resistance mechanism that is exerted by tumor cells in response to endogenous anti-tumor activity.

The inhibitory mechanism described above is co-opted by tumors that express PD-L1 as a way of evading immune detection and elimination. The binding of an anti–PD-L1 agent to the PD-L1 receptor inhibits the interaction of PD-L1 with the PD-1 and CD80 receptors expressed on immune cells. This activity overcomes PD-L1–mediated inhibition of antitumor immunity. While functional blockade of PD-L1 results in T-cell reactivation, this mechanism of action is different from direct agonism of a stimulatory receptor such as CD28.

PD-L1 is expressed in a broad range of cancers. Based on these findings, an anti–PD-L1 antibody could be used therapeutically to enhance antitumor immune responses in patients with cancer. Results of non-clinical and clinical studies of monoclonal antibodies (mAbs) targeting the PD-L1/PD-1 pathway have shown evidence of clinical activity and a manageable safety profile, supporting the hypothesis that an anti–PD-L1 antibody could be used to therapeutically enhance antitumor immune response in cancer patients [8] with responses that tend to be more pronounced in patients with tumors that express PD-L1[9]. In addition, high mutational burden (e.g., in bladder carcinoma) may contribute to the responses seen with immune therapy[10].

Pre-clinical data have now been added to with a wealth of clinical data showing that blockade of negative regulatory signals to T-cells such as programmed death ligand 1 (PD-L1) has promising clinical activity. Nivolumab and pembrolizumab, two anti–PD-1 agents, and atezolizumab, an anti–PD-L1, agent have been granted approvals by agencies such as the US Food and Drug Administration (FDA) and the European Medicines Agency approval for the treatment of a number of malignancies including metastatic melanoma, squamous and non-squamous cell non-small-cell lung cancer and urothelial carcinoma. In addition, there are data from agents in the anti–PD-1/PD-L1class showing clinical activity in a wide range of tumor types.

1.2.2 Durvalumab background/non-clinical and clinical experience

The non-clinical and clinical experience is fully described in the most current version of the durvalumab Investigator's Brochure.

Durvalumab is a human monoclonal antibody (mAb) of the immunoglobulin G (IgG) 1 kappa subclass that inhibits binding of PD-L1 and is being developed by AstraZeneca/MedImmune for use in the treatment of cancer (MedImmune is a wholly owned subsidiary of AstraZeneca; AstraZeneca/MedImmune will be referred to as AstraZeneca throughout this document). The proposed mechanism of action (MOA) for durvalumab is interference in the interaction of PD-L1 with PD-1 and CD80 (B7.1). Blockade of PD-L1/PD-1 and PD-L1/CD80 interactions releases the inhibition of immune responses, including those that may result in tumor elimination. *In vitro* studies demonstrate that durvalumab antagonizes the inhibitory effect of PD-L1 on primary human T cells resulting in the restored proliferation of IFN-γ [11]. *In vivo* studies have shown that durvalumab inhibits tumor growth in xenograft models via a T-cell-dependent mechanism. Based on these data, durvalumab is expected to stimulate the patient's antitumor immune response by binding to PD-L1 and shifting the balance toward an antitumor response. Durvalumab has been engineered to reduce antibody-dependent cellular cytotoxicity and complement-dependent cytotoxicity.

To date durvalumab has been given to more than 6000 patients as part of ongoing studies either as monotherapy or in combination with other anti-cancer agents. Details on the safety profile of durvalumab monotherapy are summarized in Section 1.5.2.1 and Section 6.5. Refer to the current durvalumab Investigator's Brochure for a complete summary of non-clinical and clinical information including safety, efficacy and pharmacokinetics.

1.3 Rationale for conducting this study

1.3.1 Rationale for the immunotherapies for STS

Though the conventional chemotherapy has shown to benefit patients with advanced STS, only a few treatment regimens are available. Therefore, research into new therapeutic modalities for STS is needed. When considering the important role of the expression of PD1 and PD-L1 in the progression of human malignant tumors, especially for the carcinomas and malignant melanomas, there is reason to believe that the expression of PD1 and PD-L1 could also be involved in STS pathogenesis.

For STS, we identified 43% of PD-L1 expression in STS and PD-L1 expression had worse overall survival (5-year survival rate: 48% in PD-L1 positive vs. 68% in PD-L1 negative, p=0.015)[12]. In detail, PD-L1 expression was reported 52.6% in synovial sarcoma, 37.6% in rhabdomyosarcoma, and 100% in epithelioid sarcoma (figure 1). For this orphan tumor, STS, PD-L1 targeting may be a promising strategy and favorable toxicity may warrant further combination.

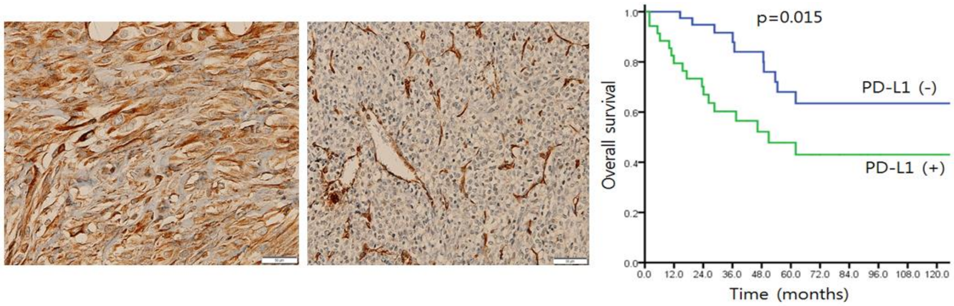

Figure 1. PD-L1 expression (left) and survival outcome (right)

In recent phase II study of pembrolizumab, PD-1 inhibitor in advanced STS [13], among the 40 STS cases, 7 cases (17.5%) had responses (1 complete response and 6 partial response). Based on the study, pembrolizumab monotherapy was approved for previously treated STS in Korea.

1.3.2 Rationale for the combination treatment

Pazopanib is an angiogenesis inhibitor targeting VEGFR-1, -2, and -3; PDGFR-α and -β; and the receptor c-Kit, and is indicated for the treatment of subjects with advanced renal cell carcinoma (RCC) and advanced STS. Pro-angiogenic factors suppress various immune functions whereas antiangiogenic agents have potential to modulate the tumor microenvironment and improve immunotherapy. Simultaneously blocking VEGF Receptor 2 and PD-1 demonstrated a synergistic antitumor effect in preclinical murine colon cancer model, with no overt toxicities [14]. The

simultaneous blockade of PD-1 and VEGF Receptor 2 inhibited tumor growth significantly compared to single-agent mAb treatment. Treatment with anti-PD-1 mAb and anti-VEGF Receptor 2 mAb induced a significant increase in the expression of several potent pro-inflammatory cytokines and mediators. Combination treatment also showed a trend toward an increase in CD4+ and CD8+ T-cell infiltration in tumor tissues

Durvalumab is a human mAb that inhibits binding of PD-L1. Simultaneously blocking VEGF Receptor 2 and PD-1 demonstrated a synergistic antitumor effect in preclinical murine colon cancer model, with no overt toxicities[14] Treatment with anti-PD-1 mAb and anti-VEGF Receptor 2 mAb induced a significant increase in the expression of several potent pro-inflammatory cytokines and mediators. Combination treatment also showed a trend toward an increase in CD4+ and CD8+ T-cell infiltration in tumor tissues[15]. Therefore, combination studies with immune checkpoint inhibitor and angiogenesis inhibitors (**ClinicalTrials.gov Identifier:** NCT02572687 durvalumab and ramucirumab; NCT02014636 pembrolizumab and pazopanib) are actively ongoing for various tumors.

1.3.3 Rationale for dose

1.3.3.1 Durvalumab dose rationale

A durvalumab dose of 20 mg/kg Q4W is supported by in-vitro data, non-clinical activity, clinical PK/pharmacodynamics, biomarkers, and activity data from Study 1108 in patients with advanced solid tumors and from a Phase I trial performed in Japanese patients with advanced solid tumor (D4190C00002).

PK/Pharmacodynamic data

Based on available PK/pharmacodynamic data from ongoing Study 1108 with doses ranging from 0.1 to 10 mg/kg Q2W or 15 mg/kg Q3W, durvalumab exhibited non-linear (dose-dependent) PK consistent with target-mediated drug disposition. The PK approached linearity at ≥3 mg/kg Q2W, suggesting near complete target saturation (membrane-bound and sPD-L1), and further shows that the durvalumab dosing frequency can be adapted to a particular regimen given the linearity seen at doses higher than 3 mg/kg. The expected half-life with doses ≥3 mg/kg Q2W is approximately 21 days. A dose-dependent suppression in peripheral sPD-L1 was observed over the dose range studied, consistent with engagement of durvalumab with PD-L1. A low level of immunogenicity has been observed. No patients have experienced immune-complex disease following exposure to durvalumab (For further information on immunogenicity, please see the current IB).

A population PK model was developed using the data from Study 1108 (doses=0.1 to 10 mg/kg Q2W or 15 mg/kg Q3W. Multiple simulations indicate that a similar overall exposure is expected following both 10 mg/kg Q2W and 20 mg/kg Q4W regimens, as represented by AUC_{ss} (4 weeks). Median $C_{max,ss}$ is expected to be higher with 20 mg/kg Q4W (~1.5 fold) and median $C_{trough,ss}$ is expected to be higher with 10 mg/kg Q2W (~1.25 fold). Clinical activity with the 20 mg/kg Q4W dosing regimen is anticipated to be consistent with 10 mg/kg Q2W with the proposed similar dose of 20 mg/kg Q4W expected to (a) achieve complete target saturation in majority of patients; (b) account for anticipated variability in PK, pharmacodynamics, and clinical activity in diverse cancer populations; (c) maintain

sufficient PK exposure in case of ADA impact; and (d) achieve PK exposure that yielded maximal antitumor activity in animal models.

Given the similar area under the plasma drug concentration-time curve (AUC) and modest differences in median peak and trough levels at steady state, the observation that both regimens maintain complete sPD-L1 suppression at trough, and the available clinical data, the 20 mg/kg Q4W and 10 mg/kg Q2W regimens are expected to have similar efficacy and safety profiles, supporting further development with a dose of 20 mg/kg Q4W.

Clinical data

Refer to the current durvalumab Investigator's Brochure for a complete summary of clinical information including safety, efficacy and pharmacokinetics at the 20mg/kg Q4W regimen.

Rationale for fixed dosing

A population PK model was developed for durvalumab using monotherapy data from a Phase I study (study 1108; N=292; doses= 0.1 to 10 mg/kg Q2W or 15 mg/kg Q3W; solid tumors). Population PK analysis indicated only minor impact of body weight (WT) on the PK of durvalumab (coefficient of ≤0.5). The impact of body WT-based (10 mg/kg Q2W) and fixed dosing (750 mg Q2W) of durvalumab was evaluated by comparing predicted steady state PK concentrations (5th, median and 95th percentiles) using the population PK model. A fixed dose of 750 mg was selected to approximate 10 mg/kg (based on median body WT of ~75 kg). A total of 1000 patients were simulated using body WT distribution of 40–120 kg. Simulation results demonstrate that body WT-based and fixed dosing regimens yield similar median steady state PK concentrations with slightly less overall between-patient variability with fixed dosing regimen.

Similar findings have been reported by others (Ng et al 2006, Wang et al 2009, Zhang et al 2012, Narwal et al 2013). Wang and colleagues investigated 12 monoclonal antibodies and found that fixed and body size-based dosing perform similarly, with fixed dosing being better for 7 of 12 antibodies ((**Error! Reference source not found.**). In addition, they investigated 18 therapeutic proteins and peptides and showed that fixed dosing performed better for 12 of 18 in terms of reducing the between-patient variability in pharmacokinetic/pharmacodynamics parameters (**Error! Reference source not found.**).

A fixed dosing approach is preferred by the prescribing community due to ease of use and reduced dosing errors. Given expectation of similar pharmacokinetic exposure and variability, we considered it feasible to switch to fixed dosing regimens. Based on average body WT of 75 kg, a fixed dose of 1500 mg Q4W or Q3W durvalumab (equivalent to 20 mg/kg Q4W) are included in the recent trials. PK modeling has been carried out to predict the effect of switching from a Q4W regimen to a Q3W regimen for both durvalumab (1500 mg; 4 doses) and tremelimumab (75 mg; 4 doses) exposures. Results suggest that a Q3W regimen would yield similar exposures to Q4W; both durvalumab and tremelimumab are expected to yield a slightly higher Cmax and Cmin on a 3 week schedule, but a lower AUC. For durvalumab, Cmax values were 660 vs. 596 (µg/mL), Cmin, were 144 vs. 94 (µg/mL), and AUC was 5879 vs.6061 (µg/mL) for Q3W and Q4W schedule, respectively. For tremelimumab, Cmax

is 26.1 vs. 25.1 (μg/mL), Cmin is 7.2 vs. 5.7 (μg/mL), and AUC is 267 vs. 289 (μg/mL), respectively for the Q3W vs. Q4W regimen. Therefore, PK modeling suggests that a Q3W schedule does not impose a significant increased safety risk based on expected durvalumab and tremelimumab exposures. Taken together, the totality of data provides sufficient safety data to support the combination of 1500mg durvalumab plus chemotherapy. Based on the study current study use fixed dose of 1500 mg Q3W durvalumab in combination with pazopanib.

1.3.3.2 Rationale for combination dose

The approved pazopanib dose regimens for the sarcoma and RCC indications will be used. Pazopanib 800 mg (daily) is approved for use as single agent for the treatment of sarcoma and RCC. Since then, current clinical trials addressed standard dose of pazopanib (800 mg) as backbone of combination treatment (ClinicalTrials.gov Identifier: pazopanib and paclitaxel combination for urothelial cancer; NCT01108055, pazopanib and paclitaxel/carboplatin combination for gynecologic tumor; NCT00561795). Therefore, we used standard dose of pazopanib and durvalumab combination in this trial

1.4 Benefit/risk and ethical assessment

1.4.1 Durvalumab

Monoclonal antibodies directed against immune checkpoint proteins, such as programmed cell death ligand 1 (PD-L1) as well as those directed against programmed cell death-1 (PD-1) or cytotoxic T-lymphocyte antigen-4 (CTLA-4), aim to boost endogenous immune responses directed against tumor cells. By stimulating the immune system however, there is the potential for adverse effects on other tissues.

Most adverse drug reactions seen with the immune checkpoint inhibitor class of agents are thought to be due to the effects of inflammatory cells on specific tissues. These risks are generally events with a potential inflammatory or immune mediated mechanism and which may require more frequent monitoring and/or unique interventions such as immunosuppressant and/or endocrine therapy. These immune mediated effects can occur in nearly any organ system, and are most commonly seen as gastrointestinal AEs such as colitis and diarrhoea, pneumonitis/interstitial lung disease (ILD), , hepatic AEs such as hepatitis and liver enzyme elevations, skin events such as rash and dermatitis and endocrinopathies including hypo- and hyper-thyroidism

Risks with durvalumab include, but are not limited to, diarrhea/colitis and intestinal perforation, pneumonitis/ILD, endocrinopathies (hypo- and hyper-thyroidism, type I diabetes mellitus, hypophysitis and adrenal insufficiency) hepatitis/increases in transaminases, nephritis/increases in creatinine, pancreatitis/increases in amylase and lipase, rash/pruritus/dermatitis, myocarditis, myositis/polymyositis, other rare or less frequent inflammatory events including neurotoxicities, infusion-related reactions, hypersensitivity reactions and infections/serious infections.

For information on all identified and potential risks with durvalumab please always refer to the current version of the durvalumab IB. Further information on these risks can be found in the current version of the durvalumab IB.

In monotherapy clinical studies AEs (all grades) reported very commonly (≥ 10% of patients) are fatigue, nausea, decreased appetite, dyspnea, cough, constipation, diarrhea, vomiting, back pain, pyrexia, asthenia, anemia, arthralgia, peripheral edema, headache, rash, and pruritus. Approximately 9% of patients experienced an AE that resulted in permanent discontinuation of durvalumab and approximately 6% of patients experienced an SAE that was considered to be related to durvalumab by the study investigator.

The majority of treatment-related AEs were manageable with dose delays, symptomatic treatment, and in the case of events suspected to have an immune basis, the use of established treatment guidelines for immune-mediated (Appendix 1)

A detailed summary of durvalumab monotherapy AE data can be found in the current version of the durvalumab IB.

1.4.2 Pazopanib

Pazopanib 800 mg (daily) is approved for use as single agent for the treatment of sarcoma and RCC. In addition, overall toxicity is mild and tolerable [grade 3-4 toxicities: fatigue (13%), diarrhea (5%), anorexia (6%)]. It is not overlapped with the toxicities of PD-1/PD-L1 inhibitors. The majority of treatment-related AEs were manageable with dose delays, symptomatic treatment.

1.4.3 Overall risk benefit

Given the unique mechanisms of action for pazopanib and durvalumab as well as the existing safety data for each, overlapping toxicities are not anticipated for the combination. However, close safety monitoring will be conducted during this study. More information about the known and expected benefits, risks, and reasonably anticipated AEs for the 2 study drugs may be found in the IBs for pazopanib and durvalumab.

2. PROVIDE SUMMARY OF OVERALL BENEFIT-RISK FOR THE STUDY OBJECTIVES

2.1 Primary objective(s)

To evaluate antitumor efficacy of durvalumab and pazopanib combination

- Overall Response Rate (ORR) will be based on RECIST version 1.1

2.2 Secondary objective(s)

1) To evaluate antitumor efficacy of durvalumab and pazopanib combination with subgroup analysis

- Progression-free survival (PFS)
- Overall survival (OS)
- Disease control rate (DCR)
- Immune-Related Response Criteria (irRC)

2) To evaluate safety of durvalumab and pazopanib combination

- Adverse events

2.3 Exploratory objective(s)

To evaluate known predictive biomarker in tumor samples

- Biomarker exploration for predictive biomarker

  ① Whole Exome Sequencing and RNA sequencing

  - Comparison between responder and non-responder

  ② PD-L1 immunohistochemistry : 22C3 (Agilent Technologies, CA, USA/Dako Products, CA, USA) and OPAL multiplex analyses

  ③ Serum cytokine evaluation

3. STUDY DESIGN

3.1 Overview of study design

This is a multi-center, phase II trial for metastatic/recurred STS after 1-2 previous chemotherapy to evaluate the clinical activity and toxicity of durvalumab in combination with pazopanib

3.2 Study schema

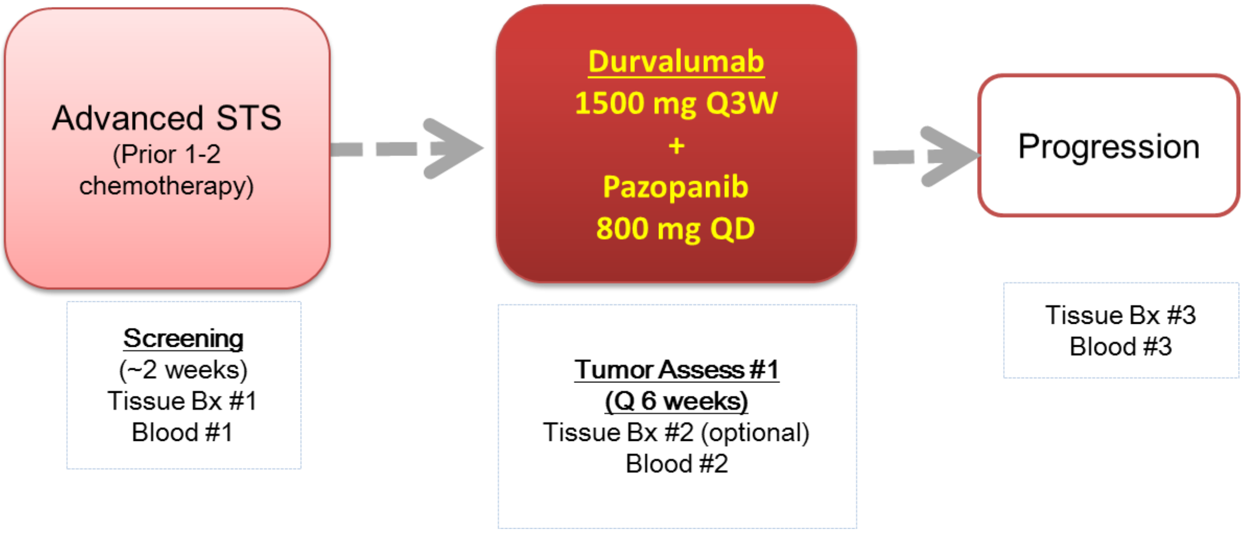

Figure 2. Study Flow Char

4. PATIENT SELECTION

4.1 Inclusion criteria

For inclusion in the study patients must fulfill all of the following criteria:

1. Histologically confirmed STS progression to 1 or 2 prior chemotherapy

   : Exclude pazopanib-resistant subtype - embryonal rhabdomyosarcoma, chondrosarcoma, osteosarcoma, Ewing tumours, primitive neuroectodermal tumour, gastrointestinal stromal tumour, dermatofibrosarcoma protuberans, inflammatory myofibroblastic sarcoma, and liposarcoma

2. Age ≥ 19 years at time of study entry.

3. Eastern Cooperative Oncology Group (ECOG) performance status of 0 or 1

4. Measurable disease by Response Evaluation Criteria in Solid Tumors Version 1.1

5. Body weight >30kg

6. Adequate normal organ and marrow function as defined below:

   – Haemoglobin ≥9.0 g/dL

   – Absolute neutrophil count (ANC) ≥ 1500 per mm^3

   – Platelet count ≥ 75,000 per mm^3

   – Serum bilirubin ≤1.5 x institutional upper limit of normal (ULN).

   – AST (SGOT)/ALT (SGPT) ≤2.5 x institutional upper limit of normal unless liver metastases are present, in which case it must be ≤5x ULN

   – Measured creatinine clearance (CL) >40 mL/min or Calculated creatinine clearance (CL)>40 mL/min by the Cockcroft-Gault formula (Cockcroft and Gault 1976)

Males:

$$\text{Creatinine CL (mL/min)} = \frac{\text{Weight (kg) x (140 – Age)}}{\text{72 x serum creatinine (mg/dL)}}$$

Females:

$$\text{Creatinine CL (mL/min)} = \frac{\text{Weight (kg) x (140 – Age)}}{\text{72 x serum creatinine (mg/dL)}} \text{ x 0.85}$$

– TSH within the normal limits OR free T3 and free T4 are within the normal limits.

7. Evidence of post-menopausal status or negative urinary or serum pregnancy test for female pre-menopausal patients. Women will be considered post-menopausal if they have been amenorrheic for 12 months without an alternative medical cause. The following age-specific requirements apply

   – Women <50 years of age would be considered post-menopausal if they have been amenorrheic for 12 months or more following cessation of exogenous hormonal treatments and if they have luteinizing hormone and follicle-stimulating hormone levels in the post-menopausal range for the institution or underwent surgical sterilization (bilateral oophorectomy or hysterectomy).

   – Women ≥50 years of age would be considered post-menopausal if they have been amenorrheic for 12 months or more following cessation of all exogenous hormonal treatments, had radiation-induced menopause with last menses >1 year ago, had chemotherapy-induced menopause with last menses >1 year ago, or underwent surgical sterilization (bilateral oophorectomy, bilateral salpingectomy or hysterectomy).

8. Patient is willing and able to comply with the protocol for the duration of the study including undergoing treatment and scheduled visits and examinations including follow up.

9. Must have a life expectancy of at least 12 weeks

10. Capable of giving signed informed consent which includes compliance with the requirements and restrictions listed in the informed consent form (ICF) and in this protocol. Written informed consent and any locally required authorization (eg, Health Insurance Portability and Accountability Act in the US, European Union [EU] Data Privacy Directive in the EU) obtained from the patient/legal representative prior to performing any protocol-related procedures, including screening evaluations

11. Patients with evidence of portal hypertension (including splenomegaly detected radiographically) or any prior history of variceal bleeding must have had endoscopic evaluation within the 3 months immediately prior to enrollment, and the findings do not represent a high bleeding risk.

4.2 Exclusion criteria

Patients should not enter the study if any of the following exclusion criteria are fulfilled:

1. More than 4 prior cytotoxic regimens
2. Participation in another clinical study with an investigational product during the last 2 weeks

   :observational (non-interventional) clinical study or during the follow-up period of an interventional study is permitted.

3. Receipt of the last dose of anticancer therapy (chemotherapy, immunotherapy, endocrine therapy, targeted therapy, biologic therapy, tumor embolization, monoclonal antibodies, other investigational agent) 14 days prior to the first dose of study drug
4. Any previous treatment with a PD1 or PD-L1 inhibitor (including durvalumab) and/or pazopanib
5. Mean QT interval corrected for heart rate (QTc) > 480 ms calculated from 3 electrocardiograms (ECGs) using Fridericia's Correction
6. Any unresolved toxicity NCI CTCAE Grade ≥2 from previous anticancer therapy with the exception of alopecia, vitiligo, and the laboratory values defined in the inclusion criteria
   - Patients with Grade ≥2 neuropathy will be evaluated on a case-by-case basis after consultation with the Study Physician.
   - Patients with irreversible toxicity not reasonably expected to be exacerbated by treatment with durvalumab may be included only after consultation with the study physician.
7. Any concurrent chemotherapy, biologic, or hormonal therapy for cancer treatment within 14 days prior to entering the study. Concurrent use of hormonal therapy for non-cancer-related conditions (e.g., hormone replacement therapy) is acceptable
8. Radiotherapy treatment to more than 30% of the bone marrow or with a wide field of radiation within 14 days of the first dose of study drug
9. Major surgical procedure (as defined by the Investigator) within 28 days prior to the first dose of IP.
10. History of allogenic organ transplantation.
11. Active or prior documented autoimmune or inflammatory disorders (including inflammatory bowel disease [e.g., colitis or Crohn's disease], diverticulitis [with the exception of diverticulosis], systemic lupus erythematosus, Sarcoidosis syndrome, or Wegener syndrome [granulomatosis with polyangiitis,

Graves' disease, rheumatoid arthritis, hypophysitis, uveitis, etc]). The following are exceptions to this criterion:

- Patients with vitiligo or alopecia
- Patients with hypothyroidism (e.g., following Hashimoto syndrome) stable on hormone replacement
- Any chronic skin condition that does not require systemic therapy
- Patients without active disease in the last 5 years may be included but only after consultation with the study physician
- Patients with celiac disease controlled by diet alone

12. Uncontrolled intercurrent illness, including but not limited to, ongoing or active infection, symptomatic congestive heart failure, uncontrolled hypertension, unstable angina pectoris (within 6 months), cardiac arrhythmia, interstitial lung disease, serious chronic gastrointestinal conditions associated with diarrhea, or psychiatric illness/social situations that would limit compliance with study requirement, substantially increase risk of incurring AEs or compromise the ability of the patient to give written informed consent

13. Known active infection

    - Active infection including tuberculosis
    - Active hepatitis B (HBsAg reactive and HBV DNA is detected)
    - Active hepatitis C (anti-HCV reactive and HCV RNA [qualitative] is detected)
    - Human immunodeficiency virus infection

14. History of another primary malignancy except for

    - Malignancy treated with curative intent and with no known active disease ≥3 years before the first dose of IP and of low potential risk for recurrence
    - Adequately treated non-melanoma skin cancer or lentigo maligna without evidence of disease
    - Adequately treated carcinoma in situ without evidence of disease

15. History of leptomeningeal carcinomatosis who are neurologically unstable or have required active treatment (any neurologic symptoms that developed either as a result of the brain metastases or their treatment must have resolved or be stable either, without the use of steroids, or are stable on a steroid dose of ≤10mg/day of prednisone or its equivalent are eligible)

16. Receipt of live attenuated vaccine within 30 days prior to the first dose of IP. Note: Patients, if enrolled, should not receive live vaccine whilst receiving IP and up to 30 days after the last dose of IP.

17. Female patients who are pregnant or breastfeeding or male or female patients of reproductive potential who are not willing to employ effective birth control from screening to 90 days after the last dose.

18. Known allergy or hypersensitivity to any of the study drugs or any of the study drug excipients.

19. History of any of the following in the past 6 months: cardiac angioplasty or stenting, myocardial infarction, unstable angina, coronary artery bypass graft surgery, symptomatic peripheral vascular disease class III or IV congestive heart failure, as defined by the New York Heart Association), thromboembolic events (patients who were stable, taking anticoagulation drugs for ≥ 6 weeks are eligible)

4.3 Withdrawal of patients from study treatment and/or study

Permanent discontinuation of study treatment

An individual patient will not receive any further investigational product if any of the following occur in the patient in question:

1. An individual patient will not receive any further durvalumab monotherapy if their weight falls to 30kg or less

2. Withdrawal of consent or lost to follow-up

3. Adverse event that, in the opinion of the investigator or the sponsor, contraindicates further dosing

4. Patient is determined to have met one or more of the exclusion criteria for study participation at study entry and continuing investigational therapy might constitute a safety risk

5. Pregnancy or intent to become pregnant

6. Any AE that meets criteria for discontinuation as defined in Section 6.3

7. Grade ≥3 infusion reaction

8. Patient noncompliance that, in the opinion of the investigator or sponsor, warrants withdrawal; e.g., refusal to adhere to scheduled visits

9. Initiation of alternative anticancer therapy including another investigational agent

10. Confirmation of PD and investigator determination that the patient is no longer benefiting from treatment with durvalumab

11. Completed 24 months (approximately 35 cycles) of durvalumab treatment.

12. Patients who are permanently discontinued from receiving investigational product will be followed for safety per Section 10.3.1, including the collection of any protocol-specified blood specimens, unless consent is withdrawn or the patient is lost to follow-up or enrolled in another clinical study. All patients will be followed for survival. Patients who decline to return to the site for evaluations will be offered follow-up by phone every 3 months as an alternative

Withdrawal of consent

Patients are free to withdraw from the study at any time (IP and assessments) without prejudice to further treatment.

Patients who withdraw consent for further participation in the study will not receive any further IP or further study observation, with the exception of follow-up for survival, which will continue until the end of the study unless the patient has expressly withdrawn their consent to survival follow-up. Note that the patient may be offered additional tests or tapering of treatment to withdraw safely.

A patient who withdraws consent will always be asked about the reason(s) for withdrawal and the presence of any AE. The Investigator will follow up AEs outside of the clinical study.

An individual patient will not receive any further IP if any of the following occur in the patient in question:

- An AE that, in the opinion of the Investigator, contraindicates further dosing
- Pregnancy or intent to become pregnant
- Non-compliance with the study protocol that, in the opinion of the Investigator, warrants withdrawal from treatment with IP (eg, refusal to adhere to scheduled visits)
- Initiation of alternative anticancer therapy including another investigational agent
- Clinical progression, i.e. Investigator determination that the patient is no longer benefiting from treatment with IP, with or without radiological progression by RECIST 1.1.

Any AE that meets criteria for discontinuation as defined in the Dosing Modification and Toxicity Management Guidelines (Appendix 1)

If a patient withdraws consent, they will be specifically asked if they are withdrawing consent to:

- all further participation in the study including any further follow up (e.g., survival contact telephone calls)
- withdrawal of consent to the use of their study generated data
- withdrawal to the use of any samples

5. INVESTIGATIONAL PRODUCT(S)

5.1 Durvalumab

The Investigational Products Supply section of AstraZeneca/MedImmune will supply durvalumab to the investigator as a 500-mg vial solution for infusion after dilution.

5.1.1 Formulation/packaging/storage

Durvalumab will be supplied by AstraZeneca as a 500-mg vial solution for infusion after dilution. The solution contains 50 mg/mL durvalumab, 26 mM histidine/histidine-hydrochloride, 275 mM trehalose dihydrate, and 0.02% weight/volume (w/v) polysorbate 80; it has a pH of 6.0. The nominal fill volume is 10.0 mL. Investigational product vials are stored at 2°C to 8°C (36°F to 46°F) and must not be frozen. Drug product should be kept in secondary packaging until use to prevent excessive light exposure.

5.1.2 Study drug preparation

Patients will receive 1500mg durvalumab via IV infusion Q3W until confirmed disease progression unless there is unacceptable toxicity, withdrawal of consent, or another discontinuation criterion is met. (If a patient's weight falls to 30kg or below the patient should not receive weight-based dosing until the weight improves to >30 kg, at which point the patient should start receiving the fixed dosing of durvalumab 1500mg Q3W).

The dose of durvalumab (MEDI4736) for administration must be prepared by the Investigator's or site's designated IP manager using aseptic technique. Total time from needle puncture of the durvalumab (MEDI4736) vial to the start of administration should not exceed:

- 24 hours at 2°C to 8°C (36°F to 46°F)
- 4 hours at room temperature

Infusion solution must be allowed to equilibrate to room temperature prior to commencement of administration. A dose of 1500mg (for patients >30kg in weight) will be administered using an IV bag containing 0.9% (w/v) saline or 5% (w/v) dextrose, with a final durvalumab concentration ranging from 1 to 15 mg/mL, and delivered through an IV administration set with a 0.2- or 0.22-μm in-line filter. Add 30.0 mL of durvalumab (ie, 1500mg of durvalumab [MEDI4736]) to the IV bag. The IV bag size should be selected such that the final concentration is within 1 to 15 mg/mL. Mix the bag by gently inverting to ensure homogeneity of the dose in the bag.

Standard infusion time 1 hour. In the event that there are interruptions during infusion, the total allowed infusion time should not exceed 8 hours at room temperature. Do not co-administer other drugs through the same infusion line.

The IV line will be flushed with a volume of IV diluent equal to the priming volume of the infusion set used after the contents of the IV bag are fully administered, or complete the infusion according to institutional policy to ensure the full dose is administered and document if the line was not flushed. If either

preparation time or infusion time exceeds the time limits a new dose must be prepared from new vials. Durvalumab does not contain preservatives, and any unused portion must be discarded.

5.1.3 Monitoring of dose administration

Patients will be monitored before, during and after the infusion with assessment of vital signs at the times specified in the Schedule of Assessment. Patients are monitored (pulse rate, blood pressure) every 30 minutes during the infusion period (including times where infusion rate is slowed or temporarily stopped).

In the event of a ≤Grade 2 infusion-related reaction, the infusion rate of study drug may be decreased by 50% or interrupted until resolution of the event (up to 4 hours) and re-initiated at 50% of the initial rate until completion of the infusion. For patients with a ≤Grade 2 infusion-related reaction, subsequent infusions may be administered at 50% of the initial rate. Acetaminophen and/or an antihistamine (e.g., diphenhydramine) or equivalent medications per institutional standard may be administered at the discretion of the investigator. If the infusion-related reaction is Grade 3 or higher in severity, study drug will be discontinued. The standard infusion time is one hour, however if there are interruptions during infusion, the total allowed time from infusion start to completion of infusion should not exceed 4 hours at room temperature, with maximum total time at room temperature not exceeding 4 hours (otherwise requires new infusion preparation). For management of patients who experience an infusion reaction, please refer to the toxicity and management guidelines in.

As with any antibody, allergic reactions to dose administration are possible. Appropriate drugs and medical equipment to treat acute anaphylactic reactions must be immediately available, and study personnel must be trained to recognize and treat anaphylaxis. The study site must have immediate access to emergency resuscitation teams and equipment in addition to the ability to admit patients to an intensive care unit if necessary.

5.2 Pazopanib

5.2.1 Formulation/packaging/storage

The contents of the label will be in accordance with all applicable regulatory requirements and supplied as standard chemotherapy.

Study medications must be dispensed or administered according to procedures described herein. Only subjects enrolled in the study may receive study medication, in accordance with all applicable regulatory requirements. Only authorized site staff may supply or administer study medication. All study medications must be stored in a secure area with access limited to the investigator and authorized site staff and under physical conditions that are consistent with the specific requirements for the study medications. Pazopanib should be stored at room temperature up to 25°C. Study medications in unopened bottles are stable until the date indicated on the package when stored at the above condition.

5.2.2 Doses, treatment regimens, and administration

Starting on Day 1 of the Treatment Period, each subject will receive 800mg (2 X 400mg tablets) of pazopanib to be administered once daily by mouth. The 200mg tablets of pazopanib will be provided to

subjects who need dose adjustments during the study. Pazopanib should be taken at least one hour before or at least two hours after a meal. The time of day for administration of study medication should be relatively constant. If a subject misses a dose, the subject should take the dose as soon as possible, but not less than 12 hours before the next dose is due. If the next dose is due in less than 12 hours, the subject should skip the missed dose and take the next dose as scheduled. In the event of vomiting at any time after taking a dose of study medication, subjects should wait until the time of the next scheduled dose to take study medication.

If dose reduction is necessary, two dose reductions are permitted in a stepwise fashion (initially to 600mg and subsequently to 400mg if necessary).

5.3 Accountability and disposition of IP

The investigator is responsible for keeping accurate records of the clinical supplies received from designee, the amount dispensed to and returned by the subjects and the amount remaining at the conclusion of the trial. Upon completion or termination of the study, all unused and/or partially used investigational product will be destroyed at the site per institutional policy. It is the Investigator’s responsibility to arrange for disposal of all empty containers, provided that procedures for proper disposal have been established according to applicable federal, state, local and institutional guidelines and procedures, and provided that appropriate records of disposal are kept.

6. TREATMENT PLAN

6.1 Patient enrollment

Patients will receive durvalumab and pazopanib combination. Treatment will be given in 3-week cycles as outlined below. The treatment to be used in this trial is outlined below in Table 1.

6.2 Dosage and administration

Drug	Dose/ Potency	Frequency	Treatment Period (Q3 weeks)	Route of Administration
**Durvalumab**	1500 mg	Q3W	Day 1	IV infusion
**Pazopanib**	800 mg	QD	Day 1-21	p.o.

Table 1. dose and schedule of IPs

All trial treatments will be administered in the order presented below; durvalumab followed by pazopanib. Both drugs should be administered on Day 1 of each cycle after all procedures/assessments have been completed as detailed on the Trial Flow Chart. Durvalumab/pazopanib may be administered up to 3 days before or after the scheduled Day 1 of each cycle due to administrative reasons.

Note: Dosing of each drugs may be withheld in the case of medical / surgical events or logistical reasons (i.e. elective surgery, unrelated medical events, subject vacation, holidays) not related to study therapy. Subjects should be placed back on study therapy within 3 weeks of the scheduled interruption to remain aligned with the Q 3W dosing interval. The reason for withholding dosing of either drugs should be documented in the subject's study record.

- Durvalumab will be administered over a 60 (±5)-minute IV infusion. The first 2 infusions will require a 1-hr observation period after the infusion. For all infusions thereafter, no observation period between infusions will be required unless clinically indicated.

- Pazopanib 800mg will be administered orally once daily without food (at least 1 hour before or 2 hours after a meal). The time of day for administration of study medication should be relatively constant. If a subject misses a dose, the subject should take the dose as soon as possible, but not less than 12 hours before the next dose is due. If the next dose is due in less than 12 hours, the subject should skip the missed dose and take the next dose as scheduled. In the event of vomiting at any time after taking a dose of study medication, subjects should wait until the time of the next scheduled dose to take study medication.

6.3 Toxicity management guidelines

6.3.1 Dose selection

Reduction or holding of one agent and not the other agents is appropriate if, in the opinion of the Investigator, the toxicity is clearly related to one of the study drugs. If, in the opinion of the Investigator, the toxicity is related to the combination of two agents, both drugs should be reduced or held according to recommended dose modifications. If one or more study agent(s) are held for toxicity, the schedule for restarting the agent(s) should correspond with the next treatment cycle once the toxicity has resolved according to the recommended guidelines. If any of the individual study drugs must be delayed for a day or more, all agents should be delayed for the same timeframe. If a patient requires a dose delay of durvalumab or pazopanib for > 6 weeks, due to toxicity, the treatment regimen will be permanently discontinued for unacceptable toxicity. If, in the judgement of investigator, toxicity is clearly related to the one specific drug, the other may continue.

Table 2. Dose modification for IP drugs

	**Level 0**	**Level -1**	**Level -2**
**Durvalumab**	1500 mg fixed dose	Dose reductions are not permitted	Dose reductions are not permitted
**Pazopanib**	800 mg	600 mg	400 mg

6.3.2 Durvalumab

Guidelines for the management of immune-mediated reactions, infusion-related reactions, and non-immune-mediated reactions for durvalumab are provided in the durvalumab Toxicity Management Guidelines. The most current version of the TMGs (toxicity management guidelines) is also available through the following link: https://tmg.azirae.com.

Patients should be thoroughly evaluated and appropriate efforts should be made to rule out neoplastic, infectious, metabolic, toxin, or other etiologic causes of the imAE. Serologic, immunologic, and histologic (biopsy) data, as appropriate, should be used to support an imAE diagnosis. In the absence of a clear alternative etiology, events should be considered potentially immune related.

In addition, there are certain circumstances in which durvalumab should be permanently discontinued (see section 4.3. of this protocol and the Dosing Modification and Toxicity Management Guidelines in APPENDIX 1). Following the first dose of IP, subsequent administration of durvalumab can be modified based on toxicities observed as described in the Dosing Modification and Toxicity Management Guidelines in **Error! Reference source not found.** These guidelines have been prepared to assist the Investigator in the exercise of his/her clinical judgment in treating these types of toxicities.

Patients should be thoroughly evaluated and appropriate efforts should be made to rule out neoplastic, infectious, metabolic, toxin, or other etiologic causes of the imAE. Serologic, immunologic, and histologic (biopsy) data, as appropriate, should be used to support an imAE diagnosis. In the absence of a clear alternative etiology, events should be considered potentially immune related.

All toxicities will be graded according to NCI CTCAE, Version 4.03.

6.3.3 Pazopanib

Table 3. Dose modification s for hematologic toxicity

**Event**	**Action**
**Neutropenia/thrombocytopenia**	
**Grade 1 or 2**	Continue study treatment at same dose; monitor as clinically indicated
**Grade 3 lasting ≥5 days**	Step 1. Interrupt study treatment until toxicity reduced to ≤ Grade 2.<br><br>Step 2. Restart study treatment same dose.
**Grade 4**	Step 1. Interrupt study treatment until toxicity reduced to ≤ Grade 2.<br><br>Step 2. Restart study treatment lower dose.
**Recurrent Grade 3/4 event after initial dose reduction**	Step 1. Interrupt study treatment until toxicity reduced to ≤ Grade 2.<br><br>Step 2. Restart study treatment lowest dose (400 mg)

If AE is considered independently related to a specific drug, reduction or holding one agent and not the other agents is permitted based on the investigator's opinion.

Table 4. Dose modification s for non-hematologic toxicity

**Event**	**Action**

**Hypertension**	
(A). Asymptomatic and persistent SBP of ≥150 and <170 mmHg, or DBP ≥90 and <110 mmHg, or a clinically significant increase in DBP of ≥20 mmHg.	Step 1. Continue study treatment at same dose.<br><br>Step 2. Adjust current dose of or initiate new antihypertensive medication(s).<br><br>Step 3. Titrate antihypertensive medication(s) during next 2 weeks as indicated to achieve well-controlled1 blood pressure (BP). If BP is not well-controlled within 2 weeks, follow Step 1 in scenario (B).
(B). Symptomatic, or SBP ≥170 mmHg, or DBP ≥110 mmHg, or failure to achieve well-controlled BP within 2 weeks in scenario (A).	Step 1. Interrupt study treatment.<br><br>Step 2. Adjust current or initiate new antihypertensive medication(s).<br><br>Step 3. Titrate antihypertensive medication(s) during next 2 weeks as indicated to achieve well-controlled BP.<br><br>Step 4 Restart study treatment at same dose or lower dose at discretion of investigator2 once BP is well-controlled1.
(C). Two or more symptomatic episodes of hypertension despite modification of antihypertensive medication(s) and reduction of study medication dose.	Discontinuation of study treatment and follow-up per protocol.
**Cardiac toxicity**	
Grade 1	Continue at same dose level

Grade 2	Continue at same dose level.<br><br>If asymptomatic decrease of LVEF by absolute value of 20% and to<LLN or nonurgent ventricular paroxysmal dysrhythmia requiring intervention:<br><br>Step 1. Interrupt study treatment until toxicity reduced ≤ Grade 1.<br><br>Step 2. Restart treatment with lower dose and monitor as clinically indicated
Grade 3	Step 1. Interrupt study treatment until toxicity reduced to ≤Grade 1.<br><br>Step 2. Restart treatment with lower dose; monitor as clinically indicated
Grade 4	Discontinuation of study treatment and follow-up per protocol
**Proteinuria**	
UPC <3	Continue study treatment at same dose. Monitor as clinically indicated.
UPC ≥3	Step 1: Obtain a 24-hr urine protein.<br><br>Step 2: If 24-hour urine protein is <3g, subject may continue treatment at same dose.<br><br>OR<br><br>If 24-hour urine protein is ≥3g, interrupt treatment until UPC returns to <3. Restart therapy at lower dose. Monitor UPC for the remainder of the overall treatment period. If UPC ≥3 obtain a 24- hour urine protein.

	Step 3: If 24-hour urine protein is ≥3g following repeat dose reductions, discontinue treatment and follow-up per protocol
**Hemorrhage/Bleeding/Coagulopathy**	
Grade 1	Continue study treatment at same dose; monitor as clinically indicated
Grade 2	Step 1. Interrupt study treatment until the AE resolves to ≤Grade 1.<br><br>Step 2. Restart treatment with lower dose; monitor as clinically indicated.
Grade 3 or 4, or Recurrent ≥Grade 2 event after dose interruption/reduction.	Discontinuation of study treatment and follow-up per protocol. Note: If abnormality is not clearly associated with clinical consequences by physician's judgement, subject may restart treatment at lower dose
**Venous Thrombosis**	
Grade 2	Continue study treatment at same dose; monitor as clinically indicated
Grade 3 or asymptomatic 4	Step 1. Interrupt study treatment.<br>Step 2. Start to treat the subject with an anticoagulant.<br>Step 3. Resume study treatment at same dose during the period of full-dose anticoagulation if all of the following criteria are met:<br>The subject must have been treated with anticoagulant for at least one week. No Grade 3 or 4 hemorrhagic events have occurred while on anticoagulation treatment<br><br>Subject should be monitored as clinically indicated during anticoagulation treatment and after resuming study treatment.

	In treating with warfarin, international normalized ratio (INR) should be monitored within three to five days after initiating, escalating/deescalating or discontinuing pazopanib therapy, and then at least weekly until the INR is stable.<br>The dose of warfarin (or its derivatives) may need to be adjusted to maintain the desired level of anticoagulation.
Symptomatic grade 4	Discontinuation of study treatment and follow-up per protocol
**Arterial thrombosis**	
Any Grade	Discontinuation of study treatment and follow-up per protocol.
**Hand-foot syndrome**	
Grade 1 and 2	Continue study treatment at same dose; monitor as clinically indicated
Grade 3	Step 1. Interrupt study treatment until toxicity reduced to ≤Grade 1.<br><br>Step 2. Restart study treatment at same dose or lower dose at discretion of investigator
Grade 4	Step 1. Interrupt study treatment until toxicity reduced to ≤Grade 2.<br><br>Step 2. Restart study treatment at lower dose or discontinue at discretion of investigator.
**Other clinically significant adverse events**	
Grade 1 or 2	Continue study treatment at same dose; monitor as clinically indicated

Grade 3	Step 1. Interrupt study treatment until the AE resolves to ≤Grade 1.<br><br>Step 2. Restart treatment with same dose or lower dose at discretion of investigator
Recurrent grade 3	Step 1. Interrupt study treatment until the AE resolves to ≤Grade 1.<br><br>Step 2. Restart treatment with lower dose
Grade 4	Step 1. Interrupt study treatment until the AE resolves to ≤Grade 2.<br><br>Step 2. Restart treatment with same dose or discontinue at discretion of investigator

7. RESTRICTIONS DURING THE STUDY AND CONCOMITANT TREATMENT(S)

7.1 Restrictions during the study

The following restrictions apply while the patient is receiving study treatment and for the specified times before and after:

Female patient of child-bearing potential

– Female patients of childbearing potential who are not abstinent and intend to be sexually active with a non-sterilized male partner must use at least 1 highly effective method of contraception from the time of screening throughout the total duration of the drug treatment and the drug washout period (90 days after the last dose of IP). Non-sterilised male partners of a female patient of childbearing potential must use male condom plus spermicide throughout this period. Cessation of birth control after this point should be discussed with a responsible physician. Periodic abstinence, the rhythm method, and the withdrawal method are not acceptable methods of birth control. Female patients should also refrain from breastfeeding throughout this period.

Male patients with a female partner of childbearing potential

– Non-sterilized male patients who are not abstinent and intend to be sexually active with a female partner of childbearing potential must use a male condom plus spermicide from the time of screening throughout the total duration of the drug treatment and the drug washout period (90 days after the last dose of IP). However, periodic abstinence, the rhythm method, and the withdrawal method are not acceptable methods of contraception. Male patients should refrain from sperm donation throughout this period.

– Female partners (of childbearing potential) of male patients must also use a highly effective method of contraception throughout this period .

Females of childbearing potential are defined as those who are not surgically sterile (ie, bilateral salpingectomy, bilateral oophorectomy, or complete hysterectomy) or post-menopausal.

Women will be considered post-menopausal if they have been amenorrheic for 12 months without an alternative medical cause. The following age-specific requirements apply:

- Women <50 years of age would be considered post-menopausal if they have been amenorrheic for 12 months or more following cessation of exogenous hormonal treatments and if they have luteinizing hormone and follicle-stimulating hormone levels in the post-menopausal range for the institution.

- Women ≥50 years of age would be considered post-menopausal if they have been amenorrheic for 12 months or more following cessation of all exogenous hormonal treatments, had radiation-

induced menopause with last menses >1 year ago, had chemotherapy-induced menopause with last menses >1 year ago.

Highly effective methods of contraception, defined as one that results in a low failure rate (ie, less than 1% per year) when used consistently and correctly are described in Table . Note that some contraception methods are not considered highly effective (e.g. male or female condom with or without spermicide; female cap, diaphragm, or sponge with or without spermicide; non-copper containing intrauterine device; progestogen-only oral hormonal contraceptive pills where inhibition of ovulation is not the primary mode of action [excluding Cerazette/desogestrel which is considered highly effective]; and triphasic combined oral contraceptive pills).

Table 5. Highly Effective Methods of Contraception (<1% Failure Rate)

• Barrier/Intrauterine methods	• Hormonal Methods
• Copper T intrauterine device<br>• Levonorgestrel-releasing intrauterine system (e.g., Mirena®)[a]	• Implants: Etonogestrel-releasing implants: e.g. Implanon® or Norplant®<br>• Intravaginal: Ethinylestradiol/etonogestrel-releasing intravaginal devices: e.g. NuvaRing®<br>• Injection: Medroxyprogesterone injection: e.g. Depo-Provera®<br>• Combined Pill: Normal and low dose combined oral contraceptive pill<br>• Patch: Norelgestromin/ethinylestradiol-releasing transdermal system: e.g. Ortho Evra®<br>• Minipillc: Progesterone based oral contraceptive pill using desogestrel: Cerazette® is currently the only highly effective progesterone-based

[a] This is also considered a hormonal method

Blood donation

Patients should not donate blood while participating in this study (at least 90 days following the last dose of IP).

7.2 Concomitant treatment(s)

7.2.1 Permitted concomitant medications

Table 6. Supportive Medications

Supportive medication/class of drug:	Usage:

Concomitant medications or treatments (e.g., acetaminophen or diphenhydramine) deemed necessary to provide adequate prophylactic or supportive care, except for those medications identified as "prohibited," as listed above	To be administered as prescribed by the Investigator
Best supportive care (including antibiotics, nutritional support, correction of metabolic disorders, optimal symptom control, and pain management [including palliative radiotherapy to non-target lesions, etc])	Should be used, when necessary, for all patients
Inactivated viruses, such as those in the influenza vaccine	Permitted

7.2.2 Excluded concomitant medications

Table 7. Prohibited Concomitant Medications for durvalumab

Prohibited medication/class of drug:	Usage:
Any investigational anticancer therapy other than those under investigation in this study	Should not be given concomitantly whilst the patient is on study treatment
mAbs against CTLA-4, PD-1, or PD-L1 other than those under investigation in this study	Should not be given concomitantly whilst the patient is on study treatment
Any concurrent chemotherapy, radiotherapy, immunotherapy, or biologic or hormonal therapy for cancer treatment other than those under investigation in this study	Should not be given concomitantly whilst the patient is on study treatment. (Concurrent use of hormones for non-cancer-related conditions [e.g., insulin for diabetes and hormone replacement therapy] is acceptable. Local treatment of isolated lesions, excluding target lesions, for palliative intent is acceptable [e.g., by local surgery or radiotherapy])

Prohibited medication/class of drug:	Usage:
Immunosuppressive medications including, but not limited to, systemic corticosteroids at doses exceeding 10 mg/day of prednisone or equivalent, methotrexate, azathioprine, and tumor necrosis factor-α blockers	Should not be given concomitantly, or used for premedication prior to the I-O infusions. The following are allowed exceptions:<br>• Use of immunosuppressive medications for the management of IP-related AEs,<br>• Short-term premedication for patients receiving combination agent pazopanib where the prescribing information for the agent requires the use of steroids for documented hypersensitivity reactions<br>• Use in patients with contrast allergies.<br>• In addition, use of inhaled, topical, and intranasal corticosteroids is permitted.<br><br>A temporary period of steroids will be allowed if clinically indicated and considered to be essential for the management of non-immunotherapy related events experienced by the patient (e.g., chronic obstructive pulmonary disease, radiation, nausea, etc).
Live attenuated vaccines	Should not be given through 30 days after the last dose of IP
Herbal and natural remedies which may have immune-modulating effects	Should not be given concomitantly unless agreed by the sponsor

Table 8. Prohibited Concomitant Medications

Substrates for the CYP450 enzyme : from 14 days prior to the first dose of pazopanib through discontinuation
Oral hypoglycemics: tolbutamide, chlorpropamide<br>• Ergot derivatives: dihydroergotamine, ergonovine, ergotamine, methylergonovine<br>• Neuroleptics: pimozide<br>• Antiarrhythmics: amiodarone, bepridil, flecainide, lidocaine, mexilitine, quinidine, propafenone<br>• Immune modulators: cyclosporine, tacrolimus, sirolimus.<br>• Miscellaneous: theophylline, quetiapine, risperidone, tacrine, clozapine, atomoxetine.

8. STUDY PROCEDURES

8.1 Schedule of study procedures

Before study entry, throughout the study, and following study drug discontinuation, various clinical and diagnostic laboratory evaluations are outlined. The purpose of obtaining these detailed measurements is to ensure adequate safety and tolerability assessments. Clinical evaluations and laboratory studies may be repeated more frequently if clinically indicated. The Schedules of Assessments during the screening and treatment period is provided following the Protocol Synopsis

For all treatment arms

- Tumor efficacy (RECIST) assessment dates are not affected by dose delays and remain as originally scheduled, as they are based on the date of first day of cycle 1.
- All other scheduled assessments must be performed relative to the start of the dosing cycle such that all laboratory procedures, etc required for dosing should be performed within 3 days prior to dosing.
- Patients may delay dosing under certain circumstances.
  - Dosing may be delayed per Toxicity Management Guidelines, due to either an immune or a non-immune-related AE.
  - If dosing must be delayed for reasons other than treatment-related toxicity, dosing will resume as soon as feasible
  - Dosing intervals of subsequent cycles may be shortened as clinically feasible in order to gradually align treatment cycles with the schedule of tumor efficacy (RECIST) assessments. Subsequent time between 2 consecutive doses cannot be less than 22 days, based on the half-lives of durvalumab
- Patients may delay and subsequently resume dosing per local standard clinical practice.

If dosing must be delayed for reasons other than treatment-related toxicity, dosing will occur as soon as feasible.

8.1.1 Screening phase

Screening procedures will be performed up to 28 days before Day 1, unless otherwise specified. All patients must first read, understand, and sign the IRB/REB/IEC-approved ICF before any study-specific screening procedures are performed. After signing the ICF, completing all screening procedures, and being deemed eligible for entry, patients will be enrolled in the study. Procedures that are performed prior to the signing of the ICF and are considered standard of care may be used as screening assessments if they fall within the 28-day screening window.

The following procedures will be performed during the Screening Visit:

- Informed Consent
- Review of eligibility criteria
- Medical history and demographics
- Complete physical exam
- ECOG Performance Status
- Vitals signs, weight and height
- 12-lead ECG (in triplicate [2-5 minutes apart])
- Tumor biopsy
- Review of prior/concomitant medications
- Imaging by CT/MRI
- Clinical laboratory tests for:
  - Hematology
  - Clinical chemistry
  - Thyroid Function Test: TSH, free T4, free T3
  - Coagulation (PT, PTT, INR)
  - Creatinine Clearance
  - Serum pregnancy test (for women of childbearing potential only)
  - Hepatitis/HIV serologies
  - Urinalysis

8.1.2 Treatment phase

Procedures to be conducted during the treatment phase of the study are presented in the Schedule of Assessments. Screening procedures performed within 72 hours of Cycle 1 Day 1 (C1D1) do not need to be repeated on C1D1.

8.1.3 End of treatment

End of treatment is defined as the last planned dosing visit within the 30 days dosing period. All required procedures may be completed within ±14 days of the end of treatment visit. Repeat disease assessment is not required if performed within ± 28 days prior to the end of treatment visit (discontinuation due to disease progression, toxicity, or patients' refusal).

8.2 Description of study procedures

8.2.1 Medical history and physical examination, electrocardiogram, weight, and vital signs

Findings from medical history (obtained at screening) and physical examination shall be given a baseline grade according to the procedure for AEs. Increases in severity of pre-existing conditions during the study will be considered AEs, with resolution occurring when the grade returns to the pre-study grade or below.

A complete physical examination will be performed and will include an assessment of the following (as clinically indicated): general appearance, respiratory, cardiovascular, abdomen, skin, head and neck (including ears, eyes, nose and throat), lymph nodes, thyroid, musculoskeletal (including spine and extremities), genital/rectal, and neurological systems and at screening only, height.

Resting 12-lead ECGs will be recorded at screening and as clinically indicated throughout the study. ECGs should be obtained after the patient has been in a supine position for 5 minutes and recorded while the patient remains in that position. At Screening, a single ECG will be obtained on which QTcF must be ≤480 ms. In case of clinically significant ECG abnormalities, including a QTcF value >480 ms, 2 additional 12-lead ECGs should be obtained over a brief period (e.g., 30 minutes) to confirm the finding.

Vital signs (blood pressure [BP], pulse, temperature, and respiration rate) will be evaluated according to the assessment schedules Body weight is also recorded at each visit along with vital signs.

First infusion

On the first infusion day, patients will be monitored and vital signs collected/recorded in CRF prior to, during and after infusion of IP as presented in the bulleted list below. BP and pulse will be collected from patients before, during, and after each infusion at the following times (based on a 60-minute infusion):

- Prior to the beginning of the infusion (measured once from approximately 30 minutes before up to 0 minutes [i.e., the beginning of the infusion])
- Approximately 30 minutes during the infusion (**halfway** through infusion)
- At the end of the infusion (approximately 60 minutes ±5 minutes)

If the infusion takes longer than 60 minutes, then BP and pulse measurements should follow the principles as described above or be taken more frequently if clinically indicated. A 1-hour observation period is recommended after the first infusion of durvalumab and tremelimumab.

Subsequent infusions

BP, pulse and other vital signs should be measured, collected/recorded in CRF prior to the start of the infusion. Patients should be carefully monitored and BP and other vital signs should be measured during and post infusion as per institution standard and as clinically indicated. Any clinically

8.2.2 Clinical laboratory tests

The following clinical laboratory tests will be performed

Table 9. Hematology Laboratory Tests

Basophils	Mean corpuscular volume
Eosinophils	Monocytes
Hematocrit	Neutrophils
Hemoglobin	Platelet count
Lymphocytes	Red blood cell count
Mean corpuscular hemoglobin	Total white cell count
Mean corpuscular hemoglobin concentration	

Note: Can be recorded as absolute counts or as percentages. Absolute counts will be calculated by data managerif entered as percentage. Total white cell count therefore has to be provided.

Table 10. Clinical Chemistry (Serum or Plasma) Laboratory Tests

Albumin	Glucose
Alkaline phosphatase	Lactate dehydrogenase
Alanine aminotransferase	Lipase
Amylase	Magnesium
Aspartate aminotransferase	Potassium
Bicarbonate	Sodium
Calcium	Total bilirubin[a]
Chloride	Total protein
Creatinine	BUN
	Uric acid

Tests for ALT, AST, alkaline phosphatase, and total bilirubin must be conducted and assessed concurrently. If total bilirubin is ≥2 × upper limit of normal (and no evidence of Gilbert’s syndrome) then fractionate into direct and indirect bilirubin.

Creatinine Clearance will be calculated by data management using Cockcroft-Gault (using actual body weight).

Table 11. Urinalysis Tests[a]

Bilirubin	pH
Blood	Protein
Glucose	Specific gravity
Ketones	Colour and appearance

[a] Microscopy should be used as appropriate to investigate white blood cells and use the high-power field for red blood cells

If a patient shows an AST or ALT ≥3xULN together with total bilirubin ≥2xULN, refer to **Error! Reference source not found.** for further instructions on cases of increases in liver biochemistry and evaluation of Hy's Law. These cases should be reported as SAEs if, after evaluation, they meet the criteria for a Hy's law case or if any of the individual liver test parameters fulfill any of the SAE criteria.

All patients should have further chemistry profiles performed within 30 day after permanent discontinuation of IP. All patients with Grade 3 or 4 laboratory values at the time of completion or discontinuation from IP must have further tests performed until the laboratory values have returned to Grade 1 or 2, unless these values are not likely to improve because of the underlying disease.

Any clinically significant abnormal laboratory values should be repeated as clinically indicated and recorded on the CRF.

8.3 Biological sampling procedures

8.3.1 Biomarker/pharmacodynamic sampling and evaluation methods

<Blood sampling for genomic DNA>

Blood (5 mL) will be drawn into labeled 1 EDTA tube (screening and cycle 3) and prepared in Yonsei Cancer Center.

- Plasma and PBMC: 1 sample will be centrifuged within 30 minutes of collection at 0 to 5°C at 3000 rpm and the plasma withdrawn. The plasma and PBMC will be transferred to BICELL and stored at -80°C

<Blood cytokine sampling>

- Serum 5ml (1 EDTA tube) for cytokines evaluation (screening and cycle 2)

<Tumor tissue>

Tumor tissue should be submitted as a newly-acquired excisional or core needle (minimum 18 gauge) biopsy in formalin or a formalin-fixed paraffin-embedded block/slides (10 unstained slides) or fresh tumor. Due diligence should be used to make sure that the tumor specimen (not a normal adjacent or a tumor margin sample) is provided. Baseline tumor biopsy, on-treatment (between cycle 3 and 9) and post-treatment tissue collection will be done for the available cases.

Pathology notes accompanying archival tissue may also be requested. The report must be coded with the patient number. Personal identifiers, including the patient's name and initials, must be removed from the institutional pathology report prior to submission. Blocks will be sectioned and returned to the site. Slides will not be returned. If a patient withdraws consent to the use of donated samples, the samples will be disposed of/destroyed, and the action documented

-Next generation sequencing

Whole exome and RNA sequencing (tissue) on pre-treatment/after progression (if available) to detect genetic changes, tumor mutation burden

-PD-L1 testing

To ensure comparability of data across all studies of durvalumab and to gain real world experience on the performance of this assay, it is strongly encouraged that all studies that include PD-L1 testing utilize the clone 22C3 (Agilent Technologies, CA, USA/Dako Products, CA, USA).

Sample collection for PD-L1 testing

- The preferred tumor sample for the determination of a patient's PD-L1 status is the one taken following the completion of the most recent prior line of therapy. Samples taken at this time reflect the current PD-L1 status of the tumor and considered clinically most relevant.

- The preferred sample for PD-L1 testing was less than or equal to 3 months old. In cases where a sample a less than 3 months old was not available, patients were asked to undergo a new biopsy if considered clinically appropriate by their treating physician.

- Samples should be collected via a core needle of 18 gauge or larger or be collected by an incisional or excisional tumor biopsy. Where institutional practice uses a smaller gauge needle, samples should be evaluated for tumor cell quantity (i.e., >100 tumor cells) to allow for adequate PD-L1 immunohistochemistry analyses.

- When the collection of a new sample is not clinically appropriate, archival samples may be utilized provided the specimen it is not older than 3 years of age. When archival samples are used to assess PD-L1 status, the age of the sample / date of collection should be captured.

- Samples submitted for PD-L1 testing should be formalin fixed and embedded in paraffin. Samples from fine needle aspirates (FNA) or decalcified bone are not appropriate for PD-L1 analysis.

The following fields of data should be collected from PD-L1 testing laboratory

- Are the negative and positive controls stained correctly
- Is the H&E material acceptable
- Is morphology acceptable
- Combined positive score (CPS) and tumor proportion score (TPS)

9. DISEASE EVALUATION AND METHODS

Patients enrolled in this study must have either measureable or evaluable disease in order to objectively evaluate the benefits of durvalumab given in combination with pazopanib. Patients should have repeat imaging of their disease every 6 weeks or at a time interval consistent with local institutional practice by CT scan or MRI (if MRI is used, CT of chest is mandatory) imaging of the chest/abdomen/pelvis (plus other regions as specifically required for specific tumor types) and other established assessments of tumor burden if CT / MRI imaging is insufficient for the individual subject. All the scans performed at baseline and other imaging performed as clinically required (other supportive imaging) need to be repeated at subsequent visits. In general, lesions detected at baseline need to be followed using the same imaging methodology and preferably the same imaging equipment at subsequent tumor evaluation visits.

RECIST 1.1 criteria will be used to assess patient response to treatment. For each subject, the investigator will designate 1 or more of the following measures of tumor status to follow for determining response: CT or MRI images of primary and/or metastatic tumor masses, physical examination findings, and the results of other assessments. All available images collected during the trial period will be considered. The most appropriate measures to evaluate the tumor status of a subject should be used. The measure(s) to be chosen for sequential evaluation during the trial have to correspond to the measures used to document the progressive tumor status that qualifies the subject for enrollment. The tumor response assessment will be assessed and listed according to the schedule of assessments.

9.1 Evaluation of Target Lesions

-Complete Response (CR): Disappearance of all target lesions Any pathological lymph nodes (whether target or non-target) must have reduction in short axis to < 10 mm.

-Partial Response (PR): At least a 30% decrease in the sum of the diameters of target lesions, taking as reference the baseline sum diameters.
- Progressive Disease (PD): At least a 20% increase in the sum of the diameters of target lesions, taking as reference the smallest sum on study (this includes the baseline sum if that is the smallest on study). In addition to the relative increase of 20%, the sum must also demonstrate an absolute increase of at least 5 mm. (Note: the appearance of one or more new lesions is also considered progressions).

-Stable Disease (SD): Neither sufficient shrinkage to qualify for PR nor sufficient increase to qualify for PD, taking as reference the smallest sum diameters while on study.

9.2 Evaluation of Non-Target Lesions

- Complete Response (CR): Disappearance of all non-target lesions. All lymph nodes must be non-pathological in size (<10 mm short axis).

- Non-CR/Non-PD: Persistence of one or more non-target lesion(s) and/or maintenance of tumor marker level above the normal limits.

- Progressive Disease (PD): Unequivocal progression (see comments below of existing non-target lesions. (Note: the appearance of one or more new lesions is also considered progression).

Note: When the patient also has measurable disease. In this setting, to achieve 'unequivocal progression' on the basis of the non-target disease, there must be an overall level of substantial worsening in non-target disease such that, even in presence of SD or PR in target disease, the overall tumor burden has increased sufficiently to merit discontinuation of therapy. A modest 'increase' in the size of one or more non-target lesions is usually not sufficient to quality for unequivocal progression status. The designation of overall progression solely on the basis of change in non-target disease in the face of SD or PR of target disease will therefore be extremely rare.

9.3 Evaluation of New Lesions

The appearance of new lesions constitutes Progressive Disease (PD). A growing lymph node that did not meet the criteria for reporting as a measurable or non-measurable lymph node at baseline should only be reported as a new lesion (and therefore progressive disease) if it:

a) increases in size to ≥15 mm in the short axis or;

b) there is new pathological confirmation that it is disease (regardless of size).

Evaluation of Tumor response as follows ;

Target Lesions	Non-target	New Lesions	Best Overall Response	Remarks
CR	CR	No	CR	
CR	Non-CR/Non-PD*	No	PR	
CR	Not evaluated	No	PR	

PR	Non-PD*/not evaluated	No	PR	
SD	Non-PD*/not evaluated	No	SD	Documented at least once ≥ 12 wks. from study entry
PD	Any	Yes or No	PD	No prior SD, PR or CR
Any	PD**	Yes or No	PD***	
Any	Any	Yes	PD	

* PD in non-target lesions should not normally trump target lesion status. It must be representative ofoverall disease status change, not a single lesion increase. Please refer to the Evaluation of NonTarget Lesions – Progressive Disease section for further explanation.

** In exceptional circumstances, unequivocal progression in non-target lesions may be accepted as disease progression.

NOTE: Patients with a global deterioration of health status requiring discontinuation of treatment without objective evidence of disease progression at that time should be reported as "symptomatic deterioration." Every effort should be made to document the objective progression even after discontinuation of treatment

The best overall response is the best response recorded from the start of the study treatment until disease progression/recurrence (taking as reference for progressive disease the smallest measurements recorded since the treatment started). The patient's best overall response assignment will depend on the achievement of both measurement and confirmation criteria as follows;

Overall response First time point	Overall Response Subsequent time point	Best Overall Response
CR	CR	CR
CR	PR	SD, PD or PR[a]
CR	SD	SD provided minimum criteria for SD duration met, otherwise PD
CR	PD	SD provided minimum criteria for SD duration met, otherwise PD

CR	NE	SD provided minimum criteria for SD duration met, otherwise PD
PR	CR	PR
PR	PR	PR
PR	SD	SD
PR	PD	SD provided minimum criteria for SD duration met, otherwise PD
PR	NE	SD provided minimum criteria for SD duration met, otherwise PD
NE	NE	NE

CR = Complete Response, PR = Partial Response, SD = Stable Disease, PD = Progressive Disease, and NE = Inevaluable

[a] If a CR is truly met at first time point, then any disease seen at a subsequent time point, even disease meeting PR criteria relative to baseline, makes the disease PD at that point (since disease must have reappeared after CR). Best response would depend on whether minimum duration for SD was met. However, sometimes 'CR' may be claimed when subsequent scans suggest small lesions were likely still present and in fact the patient had PR, not CR at the first time point. Under these circumstances, the original CR should be changed to PR and the best response is PR.

Confirmation of progression guidelines are set for the following reasons:

- For patient management and treatment decisions
- In the absence of significant clinical deterioration, to promote the collection of additional scans after the first radiologic RECIST 1.1 assessment of progressive disease (PD) in order to distinguish pseudoprogression from true radiologic progression, also known as RECIST 1.1 modified for confirmation of progression
- When scans are evaluated by Investigator, to reduce informative censoring by Investigator assessments

Confirmed objective disease progression refers to either of the following scenarios: 1. clinical progression/deterioration followed by a radiologic verification scan (PD by RECIST 1.1); or 2. in the absence of significant clinical deterioration, radiologic PD by RECIST 1.1 followed by a second radiologic confirmation scan with PD assessed according to the specific confirmation of progression criteria listed below. The confirmatory scan should occur preferably at the next scheduled imaging visit and no earlier than 4 weeks following the date of the immediate prior assessment of RECIST 1.1 PD. If progressive

disease was confirmed at the subsequent 4 weeks assessment, the date of the initial progressive disease was used for analyses and the patient stopped study treatment.

Following confirmed progression, patients should continue to be followed up for survival every 3 months (12 weeks±14 days) as outlined in the follow-up schedules of assessments

9.4 Duration of Response

Duration of overall response is measured from the time measurement criteria are met for CR or PR (whichever is first recorded) until the first date that recurrent or progressive disease is objectively documented (taking as reference for progressive disease the smallest measurements recorded since the treatment started). The duration of overall CR is measured from the time measurement criteria are first met for CR until the first date that progressive disease is objectively documented. Duration of stable disease: Stable disease is measured from the start of the treatment until the criteria for progression are met, taking as reference the smallest measurements recorded since the treatment started, including the baseline measurements.

9.5 Definition of Overall Response Using irRC

Overall response using irRC will be based on these criteria:

-Immune-Related Complete Response (irCR): Complete disappearance of all tumor lesions (index and nonindex together with no new measurable/unmeasurable lesions) for at least 4 weeks from the date of documentation of complete response.

-Immune-Related Partial Response (irPR): The sum of the products of the two largest perpendicular diameters of all index lesions is measured and captured as the SPD baseline. At each subsequent tumor assessment, the sum of the products of the two largest perpendicular diameters of all index lesions and of new measurable lesions are added together to provide the Immune Response Sum of Product Diameters (irSPD). A decrease, relative to baseline of the irSPD compared to the previous SPD baseline, of 50% or greater is considered an immune Partial Response (irPR).

-Immune-Related Stable Disease (irSD): irSD is defined as the failure to meet criteria for immune complete response or immune partial response, in the absence of progressive disease.

-Immune-Related Progressive Disease (irPD): It is recommended in difficult cases to confirm PD by serial imaging. Any of the following will constitute progressive disease:

-At least 25% increase in the sum of the products of all index lesions over baseline SPD calculated for the index lesions.

-At least a 25% increase in the sum of the products of all index lesions and new measurable lesions (irSPD) over the baseline SPD calculated for the index lesions

Index Lesion Definition	Index Lesion Definition	New Measurable Lesions	New Unmeasurable Lesions	Percent change in tumor burden (including measurable new lesions when present)	Overall irRC Response
**Complete response**	Complete response	No	No	-100%	irCR
**Partial response**	Any	Any	Any	≥-50%	irPR
**Stable disease**	Any	Any	Any	<-50% to<br><+ 25%	irSD
**Progressive disease**	Any	Any	Any	≥+25%	irPD

10. ASSESSMENT OF SAFETY

The Principal Investigator is responsible for ensuring that all staff involved in the study is familiar with the content of this section.

10.1.1 Safety parameters

10.1.1.1 Definition of adverse events

The International Conference on Harmonization (ICH) Guideline for Good Clinical Practice (GCP) E6(R1) defines an AE as:

Any untoward medical occurrence in a patient or clinical investigation patient administered a pharmaceutical product and which does not necessarily have a causal relationship with this treatment. An AE can therefore be any unfavorable and unintended sign (including an abnormal laboratory finding), symptom, or disease temporally associated with the use of a medicinal product, whether or not considered related to the medicinal product.

An AE includes but is not limited to any clinically significant worsening of a patient's pre-existing condition. An abnormal laboratory finding (including ECG finding) that requires an action or intervention by the investigator, or a finding judged by the investigator to represent a change beyond the range of normal physiologic fluctuation, should be reported as an AE.

Adverse events may be treatment emergent (i.e., occurring after initial receipt of investigational product) or nontreatment emergent. A nontreatment-emergent AE is any new sign or symptom, disease, or other untoward medical event that begins after written informed consent has been obtained but before the patient has received investigational product.

Elective treatment or surgery or preplanned treatment or surgery (that was scheduled prior to the patient being enrolled into the study) for a documented pre-existing condition, that did not worsen from baseline, is not considered an AE (serious or nonserious). An untoward medical event occurring during the prescheduled elective procedure or routinely scheduled treatment should be recorded as an AE or SAE.

The term AE is used to include both serious and non-serious AEs.

10.1.2 Definition of serious adverse events

A serious adverse event is an AE occurring during any study phase (i.e., screening, run-in, treatment, wash-out, follow-up), at any dose of the study drugs that fulfills one or more of the following criteria:

- Results in death
- Is immediately life-threatening

- Requires in-patient hospitalization or prolongation of existing hospitalization
- Results in persistent or significant disability or incapacity
- Is a congenital abnormality or birth defect in offspring of the patient
- Is an important medical event that may jeopardize the patient or may require medical intervention to prevent one of the outcomes listed above.
- Medical or scientific judgment should be exercised in deciding whether expedited reporting is appropriate in this situation. Examples of medically important events are intensive treatment in an emergency room or at home for allergic bronchospasm, blood dyscrasias, or convulsions that do not result in hospitalizations; or development of drug dependency or drug abuse.

The causality of SAEs (their relationship to all study treatment/procedures) will be assessed by the investigator(s) and communicated to AstraZeneca.

10.1.3 Definition of adverse events of special interest (AESI)

An adverse event of special interest (AESI) is one of scientific and medical interest specific to understanding of the Investigational Product and may require close monitoring. An AESI may be serious or non-serious.

If the Investigator has any questions in regards to an event being an imAE, the Investigator should promptly contact the Study Physician.

AESIs observed with durvalumab include:

- Diarrhea / Colitis and intestinal perforation
- Pneumonitis / ILD
- hepatitis / transaminase increases
- Endocrinopathies (i.e. events of hypophysitis/hypopituitarism, adrenal insufficiency, hyper- and hypothyroidism and type I diabetes mellitus)
- Rash / Dermatitis
- Nephritis / Blood creatinine increases
- Pancreatitis / serum lipase and amylase increases
- Myocarditis
- Myositis / Polymyositis

- Neuropathy / neuromuscular toxicity (e.g. Guillain-Barré, and myasthenia gravis)
- Other inflammatory responses that are rare / less frequent with a potential immune-mediated aetiology include, but are not limited to, pericarditis, sarcoidosis, uveitis and other events involving the eye, skin, haematological and rheumatological events.

In addition, infusion-related reactions and hypersensitivity/anaphylactic reactions with a different underlying pharmacological aetiology are also considered AESIs.

Further information on these risks (e.g. presenting symptoms) can be found in the current version of the durvalumab Investigator's Brochures. More specific guidelines for their evaluation and treatment are described in detail in the Dosing Modification and Toxicity Management Guidelines. These guidelines have been prepared by the Sponsor to assist the Investigator in the exercise of his/her clinical judgment in treating these types of toxicities. These guidelines apply to AEs considered causally related to the study drug/study regimen by the reporting investigator.

If new or worsening pulmonary symptoms (e.g. dyspnea) or radiological abnormality suggestive of pneumonitis/interstitial lung disease is observed, toxicity management as described in detail in the Dosing Modification and Toxicity Management Guidelines will be applied. The results of the full diagnostic workup (including high-resolution computed tomography (HRCT), blood and sputum culture, hematological parameters etc) will be captured in the CRF. It is strongly recommended to perform a full diagnostic workup, to exclude alternative causes such as lymphangitic carcinomatosis, infection, allergy, cardiogenic edema, or pulmonary hemorrhage. In the presence of confirmatory HRCT scans where other causes of respiratory symptoms have been excluded, a diagnosis of pneumonitis (ILD) should be considered and the Dosing Modification and Toxicity Management Guidelines should be followed.

Pneumonitis (ILD) investigation

The following assessments, and additional assessments if required, will be performed to enhance the investigation and diagnosis of potential cases of pneumonitis. The results of the assessment will be collected.

- Physical examination
  - Signs and symptoms (cough, shortness of breath and pyrexia, etc.) including auscultation for lung field will be assessed.
- SpO2
  - Saturation of peripheral oxygen (SpO2)
- Other items
  - When pneumonitis (ILD) is suspected during study treatment, the following markers should be measured where possible:

(i) ILD Markers (KL-6, SP-D) and β-D-glucan

(ii) Tumour markers: Particular tumour markers which are related to disease progression.

Additional Clinical chemistry: CRP, LDH

10.2 Assessment of safety parameters

10.2.1 Assessment of severity

Assessment of severity is one of the responsibilities of the investigator in the evaluation of AEs and SAEs. Severity will be graded according to the NCI CTCAE v4.03.The determination of severity for all other events not listed in the CTCAE should be made by the investigator based upon medical judgment and the severity categories of Grade 1 to 5 as defined below.

Grade 1 (mild)	An event that is usually transient and may require only minimal treatment or therapeutic intervention. The event does not generally interfere with usual activities of daily living.
Grade 2 (moderate)	An event that is usually alleviated with additional specific therapeutic intervention. The event interferes with usual activities of daily living, causing discomfort but poses no significant or permanent risk of harm to the patient.
Grade 3 (severe)	An event that requires intensive therapeutic intervention. The event interrupts usual activities of daily living, or significantly affects the clinical status of the patient.
Grade 4 (life-threatening)	An event, and/or its immediate sequelae, that is associated with an imminent risk of death or with physical or mental disabilities that affect or limit the ability of the patient to perform activities of daily living (eating, ambulation, toileting, etc).
Grade 5 (fatal)	Death (loss of life) as a result of an event.

It is important to distinguish between serious criteria and severity of an AE. Severity is a measure of intensity whereas seriousness is defined by the criteria in Section 10.1.2. A Grade 3 AE need not necessarily be considered an SAE. For example, a Grade 3 headache that persists for several hours may not meet the regulatory definition of an SAE and would be considered a nonserious event, whereas a Grade 2 seizure resulting in a hospital admission would be considered an SAE.

10.2.2 Assessment of relationship

Investigators must also systematically assess the causal relationship of AEs to IP using the following definitions. Decisive factors for the assessment of causal relationship of an AE to durvalumab and/or pazopanib include, but may not be limited to, temporal relationship between the AE medical history, concomitant medication, course of the underlying disease, trial procedures.

Not related: Not suspected to be reasonably related to the IP. AE could not medically (pharmacologically/clinically) be attributed to the IP under study in this clinical trial protocol. A reasonable alternative explanation must be available.

Related: Suspected to be reasonably related to the IP. AE could medically (pharmacologically/clinically) be attributed to the IMP under study in this clinical trial protocol.

10.3 Recording of adverse events and serious adverse events

AEs and SAEs will be collected from the time of the patient signing the informed consent form until the follow-up period is completed (30 days after the last dose of durvalumab or pazopanib). If an event that starts post the defined safety follow up period noted above is considered to be due to a late onset toxicity to study drug then it should be reported as an AE or SAE as applicable.

During the course of the study, all AEs and SAEs should be proactively followed up for each patient for as long as the event is ongoing. Every effort should be made to obtain a resolution for all events, even if the events continue after the patient has discontinued study drug or the study has completed.

Any AEs that are unresolved at the patient's last visit in the study are followed up by the Investigator for as long as medically indicated, but without further recording in the CRF. AstraZeneca retains the right to request additional information for any patient with ongoing AE(s)/SAE(s) at the end of the study, if judged necessary.

The following variables will be collected for each AE:

In addition, the following variables will be collected for SAEs as applicable:

- AE (verbatim)
- The date when the AE started and stopped
- The maximum CTCAE grade reported
- Changes in CTCAE grade
- Whether the AE is serious or not
- Investigator causality rating against the IPs (yes or no)
- Action taken with regard to IPs
- Administration of treatment for the AE
- Outcome

In addition, the following variables will be collected for SAEs:

- Date the AE met criteria for SAE
- Date the Investigator became aware of the SAE
- Seriousness criteria fulfilled
- Date of hospitalization
- Date of discharge
- Probable cause of death
- Date of death
- Whether an autopsy was performed
- Causality assessment in relation to study procedure(s)
- Causality assessment in relation to other medication, as explained in Section 10.3.2
- Description of the SAE

The grading scales found in the revised NCI CTCAE version 4.03 will be utilized for all events with an assigned CTCAE grading. For those events without assigned CTCAE grades, the recommendation in the CTCAE criteria that converts mild, moderate, and severe events into CTCAE grades should be used. A copy of the CTCAE version 4.03 can be downloaded from the Cancer Therapy Evaluation Program website (http://ctep.cancer.gov).

- Events, which are unequivocally due to disease progression, should not be reported as an AE during the study.

10.3.1 Study recording period and follow-up for adverse events and serious adverse events

Adverse events and serious adverse events will be recorded from time of signature of informed consent, throughout the treatment period and including the follow-up period (30 days after the last dose of durvalumab or pazopanib).

During the course of the study all AEs and SAEs should be proactively followed up for each patient. Every effort should be made to obtain a resolution for all events, even if the events continue after discontinuation/study completion.

If a patient discontinues from treatment for reasons other than disease progression, and therefore continues to have tumor assessments, drug or procedure-related SAEs must be captured until the patient is considered to have confirmed PD and will have no further tumor assessments.

The investigator is responsible for following all SAEs until resolution, until the patient returns to baseline status, or until the condition has stabilized with the expectation that it will remain chronic, even if this extends beyond study participation.

10.3.2 Causality collection

The Investigator will assess causal relationship between the IPs and each AE and answer "yes" or "no" to the question "Do you consider that there is a reasonable possibility that the event may have been caused by the investigational product?" For SAEs causal relationship will also be assessed for other medication and study procedures. Note that for SAEs that could be associated with any study procedure, the causal relationship is implied as "yes."

10.3.3 Adverse events based on signs and symptoms

All AEs spontaneously reported by the patient or reported in response to the open question from the study personnel: "Have you had any health problems since the previous visit/you were last asked?" or revealed by observation will be collected and recorded in the CRF. When collecting AEs, the recording of diagnoses is preferred, when possible, to recording a list of signs and symptoms. However, if a diagnosis is known and there are other signs or symptoms that are not generally part of the diagnosis, the diagnosis and each sign or symptom will be recorded separately.

10.3.4 Adverse events based on examinations and tests

The results from protocol-mandated laboratory tests and vital signs measurements will be summarized in the CSR. Deterioration as compared to baseline in protocol-mandated laboratory values and vital signs should therefore only be reported as AEs if they fulfill any of the SAE criteria or are the reason for discontinuation of treatment with the IPs.

If deterioration in a laboratory value or vital sign is associated with clinical signs and symptoms, the sign or symptom will be reported as an AE and the associated laboratory result or vital sign will be considered as additional information. Whenever possible, the reporting Investigator should use the clinical rather than the laboratory term (e.g., anemia versus low hemoglobin value). In the absence of clinical signs or symptoms, clinically relevant deteriorations in non-mandated parameters should be reported as AEs. Deterioration of a laboratory value that is unequivocally due to disease progression should not be reported as an AE/SAE. Any new or aggravated clinically relevant abnormal medical finding at a physical examination as compared with the baseline assessment will be reported as an AE.

10.3.5 Hy's Law

Cases where a patient shows elevations in liver biochemistry may require further evaluation and occurrences of AST or ALT ≥3 × ULN together with total bilirubin ≥2 × ULN may need to be reported as SAEs. Please refer to **Error! Reference source not found.** for further instruction on cases of increases in liver biochemistry and evaluation of Hy's law.

10.3.6 Disease progression

Disease progression can be considered as a worsening of a patient's condition attributable to the disease for which the IP is being studied. It may be an increase in the severity of the disease under study and/or increases in the symptoms of the disease. The development of new or progression of existing metastasis to the primary cancer under study should be considered as disease progression and not an AE. Events that are unequivocally due to disease progression should not be reported as an AE during the study.

10.3.7 Deaths

All deaths that occur during the study treatment period, or within the protocol-defined follow-up period after the administration of the last dose of study drug, must be reported as follows:

- Death clearly resulting from disease progression should be reported to the Study Monitor/Physician at the next monitoring visit and should be documented in the CRF in the Statement of Death page. It should not be reported as an SAE.
- Where death is not due (or not clearly due) to progression of the disease under study, the AE causing the death must be reported to the Study Monitor/Physician as an SAE within 24 hours. It should also be documented in the Statement of Death page in the CRF. The report should contain a comment regarding the co involvement of PD, if appropriate, and should assign main and contributory causes of death.
- Deaths with an unknown cause should always be reported as an SAE. It should also be documented in the Statement of Death page in the CRF. A post mortem may be helpful in the assessment of the cause of death, and if performed, a copy of the post-mortem results should be forwarded to AstraZeneca Patient Safety or its representative within the usual timeframes.

Deaths occurring after the protocol defined safety follow up period after the administration of the last dose of study drug should be documented in the Statement of Death page. If the death occurred as a result of an event that started after the defined safety follow up period and the event is considered to be due to a late onset toxicity to study drug, then it should also be reported as an SAE.

AstraZeneca/MedImmune retains the right to request additional information for any patient with ongoing AE(s)/SAE(s) at the end of the study, if judged necessary.

Follow-up of unresolved adverse events

Any AEs that are unresolved at the patient's last visit in the study are followed up by the investigator for as long as medically indicated, but without further recording in the CRF. After 30 days, only patients with ongoing investigational product-related SAEs will continue to be followed for safety.

AstraZeneca/MedImmune retains the right to request additional information for any patient with ongoing AE(s)/SAE(s) at the end of the study, if judged necessary.

Post-study events

After the patient has been permanently withdrawn from the study, there is no obligation for the investigator to actively report information on new AE or SAEs occurring in former study patients after the 30-day safety follow-up period for patients treated with durvalumab or pazopanib. However, if an investigator learns of any SAEs, including death, at any time after the patient has been permanently withdrawn from study, and he/she considers there is a reasonable possibility that the event is related to study treatment, the investigator should notify the study sponsor and AstraZeneca/MedImmune Drug Safety.

10.3.8 Reporting of serious adverse events

All SAEs will be reported, whether or not considered causally related to the investigational product, or to the study procedure(s). The reporting period for SAEs is the period immediately following the time that written informed consent is obtained through 90 days after the last dose of durvalumab (and/or pazopanib) or until the initiation of alternative anticancer therapy. The investigator and/or Sponsor are responsible for informing the Ethics Committee and/or the Regulatory Authority of the SAE as per local requirements.

The investigator and/or sponsor must inform the KFDA, of any serious or unexpected adverse events that occur in accordance with the reporting obligations, and will concurrently forward all such reports to AstraZeneca. A copy of the SAE or unexpected adverse events report must be emailed to AstraZeneca at the time the event is reported to the KFDA. It is the responsibility of the sponsor to compile all necessary information and ensure that the KFDA receives a report according to the KFDA reporting requirement timelines and to ensure that these reports are also submitted to AstraZeneca at the same time.

Reporting form indicating the following:

- "Notification from an Investigator Sponsored Study"
- The investigator IND number assigned by the KFDA
- The investigator's name and address
- The trial name/title and AstraZeneca ISS reference number (ESR-17-13151)

***** Sponsor must also indicate, either in the SAE report or the cover page, the ***causality*** of events ***in relation to all study medications*** and if the SAE is ***related to disease progression***, as determined by the principal investigator.

**** Send SAE report and accompanying cover page by way of email to AstraZeneca's designated mailbox:*** AEMailboxClinicalTrialTCS@astrazeneca.com
If a non-serious AE becomes serious, this and other relevant follow-up information must also be provided to AstraZeneca and the KFDA.

Serious adverse events that do not require expedited reporting to the FDA still need to be reported to AstraZeneca preferably using the MedDRA coding language for serious adverse events. This information should be reported on a monthly basis and under no circumstance less frequently than quarterly.

10.3.9 Reporting of deaths to AstraZeneca

All deaths that occur during the study, or within the protocol-defined 30-day post-last dose of durvalumab (and/or pazopanib) safety follow-up period must be reported to AstraZeneca as follows:

- Death that is clearly the result of disease progression should be documented but should not be reported as an SAE.
- Where death is not due (or not clearly due) to progression of the disease under study, the AE causing the death must be reported to AstraZeneca as a SAE within **24 hours** The report should contain a comment regarding the co-involvement of progression of disease, if appropriate, and should assign main and contributory causes of death.
- Deaths with an unknown cause should always be reported as a SAE.

Deaths that occur following the protocol-defined 90-day post-last-dose of safety follow-up period will be documented as events for survival analysis, but will not be reported as an SAE. However, if an investigator learns of any SAEs, including death, at any time after the patient has been permanently withdrawn from study, and he/she considers there is a reasonable possibility that the event is related to study treatment, the investigator should notify the study sponsor and AstraZeneca Drug Safety.

10.3.10 Other events requiring reporting

10.3.11 Overdose

An overdose is defined as a patient receiving a dose of durvalumab and/or pazopanib in excess of that specified in the Investigator's Brochure, unless otherwise specified in this protocol. Any overdose of a study patient with durvalumab, with or without associated AEs/SAEs, is required to be reported within 24 hours of knowledge of the event to the sponsor and AstraZeneca Patient Safety or designee using the designated Safety e-mailbox (see Section 10.3.2 for contact information). If the overdose results in an AE, the AE must also be recorded as an AE (see Section 10.3). Overdose does not automatically make an AE serious, but if the consequences of the overdose are serious, for example death or hospitalization, the event

is serious and must be recorded and reported as an SAE. There is currently no specific treatment in the event of an overdose of durvalumab or pazopanib.

The investigator will use clinical judgment to treat any overdose.

10.3.12 Hepatic function abnormality

Hepatic function abnormality that fulfills the biochemical criteria of a potential Hy's Law case in a study patient, with or without associated clinical manifestations, is required to be reported as "hepatic function abnormal" ***within 24 hours of knowledge of the event*** to the sponsor and AstraZeneca Patient Safety using the designated Safety e-mailbox, unless a definitive underlying diagnosis for the abnormality (e.g., cholelithiasis or bile duct obstruction) that is unrelated to investigational product has been confirmed. The criteria for a potential Hy's Law case is Aspartate Aminotransferase (AST) or Alanine Aminotransferase (ALT) ≥3x Upper Limit of Normal (ULN) together with Total Bilirubin (TBL) ≥2xULN at any point during the study following the start of study medication irrespective of an increase in Alkaline Phosphatase (ALP).

- If the definitive underlying diagnosis for the abnormality has been established and is unrelated to investigational product, the decision to continue dosing of the study patient will be based on the clinical judgment of the investigator.

- If no definitive underlying diagnosis for the abnormality is established, dosing of the study patient must be interrupted immediately. Follow-up investigations and inquiries must be initiated by the investigational site without delay.

Each reported event of hepatic function abnormality will be evaluated and followed by the investigator and AstraZeneca/MedImmune.

10.3.13 Pregnancy or maternal exposure

If a patient becomes pregnant during the course of the study or 90 days after the last dose of IPs, the IPs should be discontinued immediately. Pregnancy itself is not regarded as an AE unless there is a suspicion that the IP under study may have interfered with the effectiveness of a contraceptive medication. Congenital abnormalities or birth defects and spontaneous miscarriages should be reported and handled as SAEs. Elective abortions without complications should not be handled as AEs. The outcome of all pregnancies (spontaneous miscarriage, elective termination, ectopic pregnancy, normal birth, or congenital abnormality) should be followed up and documented even if the patient was discontinued from the study.

If any pregnancy occurs in the course of the study, then the Investigator or other site personnel should inform the appropriate AstraZeneca representatives within 1 day, i.e., immediately, but **no later than 24 hours** of when he or she becomes aware of it.

The designated AstraZeneca representative will work with the Investigator to ensure that all relevant information is provided to the AstraZeneca Patient Safety data entry site within 1 to 5 calendar days for SAEs and within 30 days for all other pregnancies.

The same timelines apply when outcome information is available.

10.3.14 Paternal exposure

Male patients should refrain from fathering a child or donating sperm during the study and for 90 days after the last dose of IPs.

Pregnancy of the patient’s partner is not considered to be an AE. However, the outcome of all pregnancies (spontaneous miscarriage, elective termination, ectopic pregnancy, normal birth, or congenital abnormality) occurring from the date of the first dose until 90 days after the last dose of IPs.

Where a report of pregnancy is received, prior to obtaining information about the pregnancy, the Investigator must obtain the consent of the patient’s partner. Therefore, the local study team should adopt the generic ICF template in line with local procedures and submit it to the relevant Ethics Committees (ECs)/Institutional Review Boards (IRBs) prior to use.

10.4 Medication error

For the purposes of this clinical study a medication error is an unintended failure or mistake in the treatment process for an AstraZeneca study drug that either causes harm to the patient or has the potential to cause harm to the patient.

A medication error is not lack of efficacy of the drug, but rather a human or process related failure while the drug is in control of the study site staff or patient.

Medication error includes situations where an error

- Occurred
- Was identified and intercepted before the patient received the drug
- Did not occur, but circumstances were recognized that could have led to an error

Examples of events to be reported in clinical studies as medication errors:

- Drug name confusion
- Dispensing error e.g. medication prepared incorrectly, even if it was not actually given to the patient
- Drug not administered as indicated, for example, wrong route or wrong site of administration
- Drug not taken as indicated e.g. tablet dissolved in water when it should be taken as a solid tablet
- Drug not stored as instructed e.g. kept in the fridge when it should be at room temperature
- Wrong patient received the medication (excluding IVRS/IWRS errors)
- Wrong drug administered to patient (excluding IVRS/IWRS errors)

Examples of events that **do not** require reporting as medication errors in clinical studies:

- Errors related to or resulting from IVRS/IWRS - including those that lead to one of the above listed events that would otherwise have been a medication error
- Patient accidentally missed drug dose(s) e.g. forgot to take medication
- Accidental overdose (will be captured as an overdose)
- Patient failed to return unused medication or empty packaging
- Errors related to background and rescue medication, or standard of care medication in open label studies, even if an AZ product

Medication errors are not regarded as AEs but AEs may occur as a consequence of the medication error.

If a medication error occurs in the course of the study, then the Investigator or other site personnel informs the appropriate AstraZeneca representatives within 1 day i.e., immediately but **no later than 24 hours** of when he or she becomes aware of it.

The designated AstraZeneca representative works with the Investigator to ensure that all relevant information is completed within 1 or 5 calendar days if there is an SAE associated with the medication error and within 30 days for all other medication errors.

11. STATISTICAL METHODS AND SAMPLE SIZE DETERMINATION

11.1 Sample Size Determination

On the basis of a prospective study with previously treated metastatic STS (Lancet Oncol 2018; 19: 416–26), five (13%) of these 40 evaluable patients achieving a confirmed response would be considered sufficient evidence of promising clinical activity in this setting

Therefore, similar with previous studies (Lancet Oncol 2018; 19: 416–26 and Lancet Oncol 2017) with immune checkpoint inhibitor for soft tissue sarcoma, this study designed to yielded 90% power to detect clinical activity if at least 20% of patients had a confirmed response at 0·05 level of significance (one-sided test), and clinical inactivity if 5% or fewer patients achieved a confirmed response.

Applying optimal design, a total of 41 evaluable patients per cohort had to be accrued. After the enrollment of first stage (n=22), interim analysis will be done to decide whether to continue the study or not. If 2 or more responses are observed in the first 22 patients, accrual will be continued until a total of 41 evaluable patients reached.

If, of these 41 patients, 5 or more responses warranted additional investigation. Allowing for a follow-up loss rate of 10 %, the total sample size is expected as 46 patients.

11.2 Description of analysis sets

11.2.1 Safety analysis set

The Safety analysis set (SAF) consists of all subjects who received at least one dose of study treatment.

11.2.2 Efficacy analysis set

Intention to treat analysis set consists of all subjects who have received at least 1 dose of trial treatment. The efficacy analysis set is radiological evaluable population includes all patients of the intention-to treat population who underwent at least one radiological assessment.

11.3 Methods of statistical analyses

The efficacy variables in this study are progression free survival (PFS), time to progression (TTP), overall response rate (ORR, percentage of patients with complete response and partial response), duration of response (DoR), clinical benefit rate (CBR, percentage of patients with complete response, partial response and stable disease), and overall survival (OS). All efficacy and exploratory analyses will be performed on the efficacy analysis set (EAS). The Kaplan-Meier approach will be used to estimate median PFS and OS with 95% CI. PFS is defined as the time between the day of first cycle and the date of first documentation of confirmed PD or date of death, whichever occurs first. TTP is defined as the time between the day of first cycle and the date of first documentation of confirmed PD. OS is defined as the time between

the day of first cycle and to death from any cause. Documentation of disease progression will be defined as per RECIST v1.1 criteria based on investigator assessment. Patients without documented confirmed PD or death will be censored at the date of last tumor assessment. The Kaplan-Meier approach will be used to estimate the median PFS and TTP duration with 95% CI. The ORR, based on investigator assessments, will be summarized and 95% confidence limits will be calculated using the Clopper-Pearson method. Safety data will be summarized and analyzed on the safety set population. Results of all efficacy analysis will be presented using a 95% confidence interval and 2-sided p-value.

12. ETHICAL AND REGULATORY REQUIREMENTS

12.1 Ethical conduct of the study

The study will be performed in accordance with ethical principles that have their origin in the Declaration of Helsinki and are consistent with ICH/Good Clinical Practice, and applicable regulatory requirements Patient data protection.

12.2 Ethics and regulatory review

The investigator is responsible for the conduct of the trial at his/her site. He/she will ensure that the trial is performed in accordance with the clinical trial protocol and with the ethical principles that have their origin in the Declaration of Helsinki, as well as with the ICH Note for Guidance on Good Clinical Practice (ICH Topic E6, 1996) and applicable regulatory requirements. In particular, the investigator must ensure that only subjects who have given their informed consent are included into the trial.

12.3 Informed consent

The investigator is responsible for ensuring that the patient understands the potential risks and benefits of participating in the study, including answering any questions the patient may have throughout the study and sharing in a timely manner any new information that may be relevant to the patient's willingness to continue his or her participation in the trial. The ICF will be used to explain the potential risks and benefits of study participation to the patient in simple terms before the patient is entered into the study, and to document that the patient is satisfied with his or her understanding of the risks and benefits of participating in the study and desires to participate in the study. This includes obtaining the appropriate signatures and dates on the ICF prior to the performance of any protocol procedures and prior to the administration of investigational product.

12.4 Audits and inspections and protocol amendment

Prior to commencement of the trial at a given site, the clinical trial protocol will be submitted together with its associated documents (such as the ICF) to the responsible IEC / IRB for its favorable opinion/approval. The written favorable opinion/approval of the IEC / IRB will be filed in the Investigator Site File. The trial must not start at a site before the Investigator has obtained written confirmation of favorable opinion/approval from the concerned IEC / IRB. The IEC / IRB will

be asked to provide documentation of the date of the meeting at which the favorable opinion/approval was given, and of the members and voting members present at the meeting. Written evidence of favorable opinion/approval that clearly identifies the trial, the clinical trial protocol version and the Subject Information and ICF version reviewed should be provided. Where possible, copies of the meeting minutes should be obtained.

Amendments to the clinical trial will also be submitted to the concerned IEC / IRB, before implementation in case of substantial changes. Relevant safety information will be submitted to the IEC/IRB during the course of the trial in accordance with national regulations and requirements.

13. STUDY MANAGEMENT

13.1 Monitoring of the study

The investigators agree to be responsible for implementing and maintaining a quality management system with written development procedures and functional area standard operating procedures (SOPs) to ensure that trials are conducted and data are generated, documented, and reported in compliance with the protocol, accepted standards of Good Clinical Practice, and all applicable federal, state, and local laws, rules and regulations relating to the conduct of the clinical trial.

According to the IDMC (Independent Drug and Safety Monitoring Committee) guideline of KFDA, we will establish IDMC organization to assist and help performing this study.

14. DATA MANAGEMENT

The investigator or qualified designee is responsible for recording and verifying the accuracy of subject data. The investigator will be responsible for entering trial data in the paper CRF provided by the Investigator. It is the investigator’s responsibility to ensure the accuracy of the data entered in the CRFs.

LIST OF REFERENCES

1. van der Graaf WTA, Blay J-Y, Chawla SP, Kim D-W, Bui-Nguyen B, Casali PG, et al. Pazopanib for metastatic soft-tissue sarcoma (PALETTE): a randomised, double-blind, placebo-controlled phase 3 trial. The Lancet. 2012;379:1879-86.
2. Van Glabbeke M, Verweij J, Judson I, Nielsen OS, Tissue ES, Bone Sarcoma G. Progression-free rate as the principal end-point for phase II trials in soft-tissue sarcomas. Eur J Cancer. 2002;38:543-9.
3. Storm HH. Survival of adult patients with cancer of soft tissues or bone in Europe. EUROCARE Working Group. Eur J Cancer. 1998;34:2212-7.
4. Yoo KH, Kim HS, Lee SJ, Park SH, Kim SJ, Kim SH, et al. Efficacy of pazopanib monotherapy in patients who had been heavily pretreated for metastatic soft tissue sarcoma: a retrospective case series. BMC Cancer. 2015;15:154.
5. Keir ME, Butte MJ, Freeman GJ, Sharpe AH. PD-1 and its ligands in tolerance and immunity. Annu Rev Immunol. 2008;26:677-704.
6. Okudaira K, Hokari R, Tsuzuki Y, Okada Y, Komoto S, Watanabe C, et al. Blockade of B7-H1 or B7-DC induces an anti-tumor effect in a mouse pancreatic cancer model. Int J Oncol. 2009;35:741-9.
7. Pardoll DM. The blockade of immune checkpoints in cancer immunotherapy. Nat Rev Cancer. 2012;12:252-64.
8. Brahmer JR, Tykodi SS, Chow LQ, Hwu WJ, Topalian SL, Hwu P, et al. Safety and activity of anti-PD-L1 antibody in patients with advanced cancer. N Engl J Med. 2012;366:2455-65.
9. Powles T, Eder JP, Fine GD, Braiteh FS, Loriot Y, Cruz C, et al. MPDL3280A (anti-PD-L1) treatment leads to clinical activity in metastatic bladder cancer. Nature. 2014;515:558-62.
10. Alexandrov LB, Nik-Zainal S, Wedge DC, Aparicio SA, Behjati S, Biankin AV, et al. Signatures of mutational processes in human cancer. Nature. 2013;500:415-21.
11. Stewart R, Morrow M, Hammond SA, Mulgrew K, Marcus D, Poon E, et al. Identification and Characterization of MEDI4736, an Antagonistic Anti-PD-L1 Monoclonal Antibody. Cancer Immunol Res. 2015;3:1052-62.
12. Kim C, Kim EK, Jung H, Chon HJ, Han JW, Shin KH, et al. Prognostic implications of PD-L1 expression in patients with soft tissue sarcoma. BMC Cancer. 2016;16:434.
13. Melissa Amber Burgess VB, Brian Andrew Van Tine, Scott Schuetze, James Hu, Sandra P. D'Angelo. Multicenter phase II study of pembrolizumab (P) in advanced soft tissue (STS) and bone sarcomas (BS): Final results of SARC028 and biomarker analyses. In: ASCO; 2017; Chicago.

14. Yasuda S, Sho M, Yamato I, Yoshiji H, Wakatsuki K, Nishiwada S, et al. Simultaneous blockade of programmed death 1 and vascular endothelial growth factor receptor 2 (VEGFR2) induces synergistic anti-tumour effect in vivo. Clin Exp Immunol. 2013;172:500-6.
15. Terme M, Colussi O, Marcheteau E, Tanchot C, Tartour E, Taieb J. Modulation of immunity by antiangiogenic molecules in cancer. Clin Dev Immunol. 2012;2012:492920.

Appendix 1. Dosing Modification and Toxicity Management Guidelines for Immune-Mediated, Infusion-Related, and Non-Immune–Mediated Reactions (MEDI4736 Monotherapy or Combination Therapy With Tremelimumab or Tremelimumab Monotherapy) 1 November 2017 Version

General Considerations	
**Dose Modifications**	**Toxicity Management**
Drug administration modifications of study drug/study regimen will be made to manage potential immune-related AEs based on severity of treatment-emergent toxicities graded per NCI CTCAE v4.03.<br>In addition to the criteria for permanent discontinuation of study drug/study regimen based on CTC grade/severity (table below), permanently discontinue study drug/study regimen for the following conditions:<br>• Inability to reduce corticosteroid to a dose of ≤10 mg of prednisone per day (or equivalent) **within 12 weeks** after last dose of study drug/study regimen<br>• Recurrence of a previously experienced Grade 3 treatment-related AE following resumption of dosing<br>**Grade 1** No dose modification<br>**Grade 2** Hold study drug/study regimen dose until Grade 2 resolution to Grade ≤1.<br>If toxicity worsens, then treat as Grade 3 or Grade 4.<br>Study drug/study regimen can be resumed once event stabilizes to Grade ≤1 after completion of steroid taper.<br>Patients with endocrinopathies who may require prolonged or continued steroid replacement can be retreated with study drug/study regimen on the following conditions:<br>1. The event stabilizes and is controlled.<br>2. The patient is clinically stable as per Investigator or treating physician's clinical judgement.<br>3. Doses of prednisone are at ≤10 mg/day or equivalent.	It is recommended that management of immune-mediated adverse events (imAEs) follows the guidelines presented in this table:<br>– It is possible that events with an inflammatory or immune mediated mechanism could occur in nearly all organs, some of them not noted specifically in these guidelines.<br>– Whether specific immune-mediated events (and/or laboratory indicators of such events) are noted in these guidelines or not, patients should be thoroughly evaluated to rule out any alternative etiology (e.g., disease progression, concomitant medications, and infections) to a possible immune-mediated event. In the absence of a clear alternative etiology, all such events should be managed as if they were immune related. General recommendations follow.<br>– Symptomatic and topical therapy should be considered for low-grade (Grade 1 or 2, unless otherwise specified) events.<br>– For persistent (>3 to 5 days) low-grade (Grade 2) or severe (Grade ≥3) events, promptly start prednisone 1 to 2 mg/kg/day PO or IV equivalent.<br>– Some events with high likelihood for morbidity and/or mortality – e.g., myo-carditis, or other similar events even if they are not currently noted in the guidelines – should progress rapidly to high dose IV corticosteroids (methylprednisolone at 2 to 4 mg/kg/day) even if the event is Grade 2, and if clinical suspicion is high and/or there has been clinical confirmation. Consider, as necessary, discussing with the study physician, and promptly pursue specialist consultation.<br>– If symptoms recur or worsen during corticosteroid tapering (28 days of taper), increase the corticosteroid dose (prednisone dose [e.g., up to 2 to 4 mg/kg/day PO or IV equivalent]) until stabilization or improvement of symptoms, then resume corticosteroid tapering at a slower rate (>28 days of taper).<br>– More potent immunosuppressives such as TNF inhibitors (e.g., infliximab) (also refer to the individual sections of the imAEs for specific type of

Appendix 1. Dosing Modification and Toxicity Management Guidelines for Immune-Mediated, Infusion-Related, and Non-Immune–Mediated Reactions (MEDI4736 Monotherapy or Combination Therapy With Tremelimumab or Tremelimumab Monotherapy) 1 November 2017 Version

General Considerations

Dose Modifications	Toxicity Management
**Grade 3** Depending on the individual toxicity, study drug/study regimen may be permanently discontinued. Please refer to guidelines below.<br>**Grade 4** Permanently discontinue study drug/study regimen.<br>Note: For Grade ≥3 asymptomatic amylase or lipase levels, hold study drug/study regimen, and if complete work up shows no evidence of pancreatitis, study drug/study regimen may be continued or resumed.<br>Note: Study drug/study regimen should be permanently discontinued in Grade 3 events with high likelihood for morbidity and/or mortality – e.g., myocarditis, or other similar events even if they are not currently noted in the guidelines. Similarly, consider whether study drug/study regimen should be permanently discontinued in Grade 2 events with high likelihood for morbidity and/or mortality – e.g., myocarditis, or other similar events even if they are not currently noted in the guidelines – when they do not rapidly improve to Grade <1 upon treatment with systemic steroids and following full taper<br>Note: There are some exceptions to permanent discontinuation of study drug for Grade 4 events (i.e., hyperthyroidism, hypothyroidism, Type 1 diabetes mellitus).	immunosuppressive) should be considered for events not responding to systemic steroids. Progression to use of more potent immunosuppressives should proceed more rapidly in events with high likelihood for morbidity and/or mortality – e.g., myocarditis, or other similar events even if they are not currently noted in the guidelines – when these events are not responding to systemic steroids.<br>– With long-term steroid and other immunosuppressive use, consider need for *Pneumocystis jirovecii* pneumonia (PJP, formerly known as *Pneumocystis carinii* pneumonia) prophylaxis, gastrointestinal protection, and glucose monitoring.<br>– Discontinuation of study drug/study regimen is not mandated for Grade 3/Grade 4 inflammatory reactions attributed to local tumor response (e.g., inflammatory reaction at sites of metastatic disease and lymph nodes). Continuation of study drug/study regimen in this situation should be based upon a benefit-risk analysis for that patient.

Specific Immune-Mediated Reactions

Severity Grade of the Event (NCI CTCAE version 4.03)	Dose Modifications	Toxicity Management
**Any Grade**	**General Guidance**	**For Any Grade:**<br>– Monitor patients for signs and symptoms of pneumonitis or ILD (new onset or worsening shortness of breath or cough). Patients should be evaluated with imaging and pulmonary function tests, including other diagnostic procedures as described below.<br>– Initial work-up may include clinical evaluation, monitoring of oxygenation via pulse oximetry (resting and exertion), laboratory work-up, and high- resolution CT scan.
**Grade 1**<br>(asymptomatic, clinical or diagnostic observations only; intervention not indicated)	No dose modifications required. However, consider holding study drug/study regimen dose as clinically appropriate and during diagnostic work-up for other etiologies.	**For Grade 1 (radiographic changes only):**<br>– Monitor and closely follow up in 2 to 4 days for clinical symptoms, pulse oximetry (resting and exertion), and laboratory work-up and then as clinically indicated.<br>– Consider Pulmonary and Infectious disease consult.
**Grade 2**<br>(symptomatic; medical intervention indicated; limiting instrumental ADL)	Hold study drug/study regimen dose until Grade 2 resolution to Grade ≤1.<br>• If toxicity worsens, then treat as Grade 3 or Grade 4.<br>• If toxicity improves to Grade ≤1, then the decision to reinitiate study drug/study regimen will be	**For Grade 2 (mild to moderate new symptoms):**<br>– Monitor symptoms daily and consider hospitalization.<br>– Promptly start systemic steroids (e.g., prednisone 1 to 2 mg/kg/day PO or IV equivalent).<br>– Reimage as clinically indicated.<br>– If no improvement within 3 to 5 days, additional workup should be considered and prompt treatment with IV methylprednisolone 2 to 4 mg/kg/day started

	based upon treating physician's clinical judgment and after completion of steroid taper.	– If still no improvement within 3 to 5 days despite IV methylprednisolone at 2 to 4 mg/kg/day, promptly start immunosuppressive therapy such as TNF inhibitors (e.g., infliximab at 5 mg/kg every 2 weeks). Caution: It is important to rule out sepsis and refer to infliximab label for general guidance before using infliximab.<br>– Once the patient is improving, gradually taper steroids over ≥28 days and consider prophylactic antibiotics, antifungals, or anti-PJP treatment (refer to current NCCN guidelines for treatment of cancer-related infections [Category 2B recommendation])[a]<br>– Consider pulmonary and infectious disease consult.<br>– Consider, as necessary, discussing with study physician.
**Grade 3 or 4**<br>(Grade 3: severe symptoms; limiting self-care ADL; oxygen indicated)<br><br>(Grade 4: life-threatening respiratory compromise; urgent intervention indicated [e.g., tracheostomy or intubation])	Permanently discontinue study drug/study regimen.	**For Grade 3 or 4 (severe or new symptoms, new/worsening hypoxia, life-threatening):**<br>– Promptly initiate empiric IV methylprednisolone 1 to 4 mg/kg/day or equivalent.<br>– Obtain Pulmonary and Infectious disease consult; consider, as necessary, discussing with study physician.<br>– Hospitalize the patient.<br>– Supportive care (e.g., oxygen).<br>– If no improvement within 3 to 5 days, additional workup should be considered and prompt treatment with additional immunosuppressive therapy such as TNF inhibitors (e.g., infliximab at 5 mg/kg every 2 weeks' dose) started. Caution: rule out sepsis and refer to infliximab label for general guidance before using infliximab.<br>– Once the patient is improving, gradually taper steroids over ≥28 days and consider prophylactic antibiotics, antifungals, and, in particular, anti-PJP treatment (refer to current NCCN guidelines for treatment of cancer-related infections [Category 2B recommendation]).[a]
**Any Grade**	**General Guidance**	**For Any Grade:**<br>– Monitor for symptoms that may be related to diarrhea/enterocolitis (abdominal pain, cramping, or changes

		in bowel habits such as increased frequency over baseline or blood in stool) or related to bowel perforation (such as sepsis, peritoneal signs, and ileus).<br>– Patients should be thoroughly evaluated to rule out any alternative etiology (e.g., disease progression, other medications, or infections), including testing for clostridium difficile toxin, etc.<br>– Steroids should be considered in the absence of clear alternative etiology, even for low-grade events, in order to prevent potential progression to higher grade event.<br>– Use analgesics carefully; they can mask symptoms of perforation and peritonitis.
**Grade 1**<br>(Diarrhea: stool frequency of <4 over baseline per day)<br>(Colitis: asymptomatic; clinical or diagnostic observations only)	No dose modifications.	**For Grade 1:**<br>– Monitor closely for worsening symptoms.<br>– Consider symptomatic treatment, including hydration, electrolyte replacement, dietary changes (e.g., American Dietetic Association colitis diet), and loperamide. Use probiotics as per treating physician's clinical judgment.
**Grade 2**<br>(Diarrhea: stool frequency of 4 to 6 over baseline per day) (Colitis: abdominal pain; mucus or blood in stool)	Hold study drug/study regimen until resolution to Grade ≤1<br>• If toxicity worsens, then treat as Grade 3 or Grade 4.<br>• If toxicity improves to Grade ≤1, then study drug/study regimen can be resumed after completion of steroid taper.	**For Grade 2:**<br>– Consider symptomatic treatment, including hydration, electrolyte replacement, dietary changes (e.g., American Dietetic Association colitis diet), and loperamide and/or budesonide.<br>– Promptly start prednisone 1 to 2 mg/kg/day PO or IV equivalent.<br>– If event is not responsive within 3 to 5 days or worsens despite prednisone at 1 to 2 mg/kg/day PO or IV equivalent, GI consult should be obtained for consideration of further

		workup, such as imaging and/or colonoscopy, to confirm colitis and rule out perforation, and prompt treatment with IV methylprednisolone 2 to 4 mg/kg/day started.<br>– If still no improvement within 3 to 5 days despite 2 to 4 mg/kg IV methylprednisolone, promptly start immunosuppressives such as infliximab at 5 mg/kg once every 2 weeks[a]. **Caution:** it is important to rule out bowel perforation and refer to infliximab label for general guidance before using infliximab.<br>– Consider, as necessary, discussing with study physician if no resolution to Grade ≤1 in 3 to 4 days.<br>– Once the patient is improving, gradually taper steroids over ≥28 days and consider prophylactic antibiotics, antifungals, and anti-PJP treatment (refer to current NCCN guidelines for treatment of cancer-related infections [Category 2B recommendation]).[a]
**Grade 3 or 4**<br>(Grade 3 diarrhea: stool frequency of ≥7 over baseline per day;<br>Grade 4 diarrhea: life threatening consequences)<br>(Grade 3 colitis: severe abdominal pain, change in bowel habits, medi-cal intervention indi-cated, peritoneal signs;<br>Grade 4 colitis:	**Grade 3**<br>Permanently discontinue study drug/study regimen for Grade 3 if toxicity does not improve to Grade ≤1 within 14 days; study drug/study regimen can be resumed after completion of steroid taper.<br><br>**Grade 4**<br>Permanently discontinue study drug/study regimen.	**For Grade 3 or 4:**<br>– Promptly initiate empiric IV methylprednisolone 2 to 4 mg/kg/day or equivalent.<br>– Monitor stool frequency and volume and maintain hydration.<br>– Urgent GI consult and imaging and/or colonoscopy as appropriate.<br>– If still no improvement within 3 to 5 days of IV methylprednisolone 2 to 4 mg/kg/day or equivalent, promptly start further immunosuppressives (e.g., infliximab at 5 mg/kg once every 2 weeks). **Caution**: Ensure GI consult to rule out bowel perforation and refer to infliximab label for general guidance before using infliximab.<br>– Once the patient is improving, gradually taper steroids over ≥28 days and consider prophylactic antibiotics, antifungals, and anti-PJP treatment (refer to current NCCN guidelines for treatment of cancer-related infections [Category 2B recommendation]).[a]

life-threatening consequences, urgent intervention indicated)		
**Any Grade**	**General Guidance**	**For Any Grade:**<br>– Monitor and evaluate liver function test: AST, ALT, ALP, and TB.<br>– Evaluate for alternative etiologies (e.g., viral hepatitis, disease progression, concomitant medications).
**Grade 1**<br>(AST or ALT >ULN and ≤3.0×ULN and/or TB > ULN and ≤1.5×ULN)	• No dose modifications.<br>• If it worsens, then treat as Grade 2 event.	**For Grade 1:**<br>– Continue LFT monitoring per protocol.
**Grade 2**<br>(AST or ALT >3.0×ULN and ≤5.0×ULN and/or TB >1.5×ULN and ≤3.0×ULN)	• Hold study drug/study regimen dose until Grade 2 resolution to Grade ≤1.<br>• If toxicity worsens, then treat as Grade 3 or Grade 4.<br>• If toxicity improves to Grade ≤1 or baseline, resume study drug/study regimen after completion of steroid taper.	**For Grade 2:**<br>– Regular and frequent checking of LFTs (e.g., every 1 to 2 days) until elevations of these are improving or resolved.<br>– If no resolution to Grade ≤1 in 1 to 2 days, consider, as necessary, discussing with study physician.<br>– If event is persistent (>3 to 5 days) or worsens, promptly start prednisone 1 to 2 mg/kg/day PO or IV equivalent.<br>– If still no improvement within 3 to 5 days despite 1 to 2 mg/kg/day of prednisone PO or IV equivalent, consider additional work up and start prompt treatment with IV methylprednisolone 2 to 4 mg/kg/day.<br>– If still no improvement within 3 to 5 days despite 2 to 4 mg/kg/day of IV methylprednisolone, promptly start immunosuppressives (i.e., mycophenolate mofetil).[a] Discuss

		with study physician if mycophenolate mofetil is not available. **Infliximab should NOT be used.**<br>– Once the patient is improving, gradually taper steroids over ≥28 days and consider prophylactic antibiotics, antifungals, and anti-PJP treatment (refer to current NCCN guidelines for treatment of cancer-related infections [Category 2B recommendation]).[a]
**Grade 3 or 4**<br>(Grade 3: AST or ALT >5.0×ULN and ≤20.0×ULN and/or TB >3.0×ULN and ≤10.0×ULN)<br><br>(Grade 4: AST or ALT >20×ULN and/or TB >10×ULN)	**For Grade 3:**<br>For elevations in transaminases ≤8 × ULN, or elevations in bilirubin ≤5 × ULN:<br>• Hold study drug/study regimen dose until resolution to Grade ≤1 or baseline<br>• Resume study drug/study regimen if elevations downgrade to Grade ≤1 or baseline within 14 days and after completion of steroid taper.<br>• Permanently discontinue study drug/study regimen if the elevations do not downgrade to Grade ≤1 or baseline within 14 days<br>For elevations in transaminases >8 × ULN or elevations in bilirubin >5 × ULN, discontinue study drug/study regimen.<br>Permanently discontinue study drug/study regimen for any case meeting Hy's law criteria (AST	**For Grade 3 or 4:**<br>– Promptly initiate empiric IV methylprednisolone at 1 to 4 mg/kg/day or equivalent.<br>– If still no improvement within 3 to 5 days despite 1 to 4 mg/kg/day methylprednisolone IV or equivalent, promptly start treatment with immunosuppressive therapy (i.e., mycophenolate mofetil). Discuss with study physician if mycophenolate is not available. **Infliximab should NOT be used.**<br>– Perform hepatology consult, abdominal workup, and imaging as appropriate.<br>– Once the patient is improving, gradually taper steroids over ≥28 days and consider prophylactic antibiotics, antifungals, and anti-PJP treatment (refer to current NCCN guidelines for treatment of cancer-related infections [Category 2B recommendation]).[a]

	and/or ALT >3 × ULN + bilirubin >2 × ULN without initial findings of cholestasis (i.e., elevated alkaline P04) and in the absence of any alternative cause.[b]<br><br>**For Grade 4:**<br>Permanently discontinue study drug/study regimen.	
**Any Grade**	**General Guidance**	**For Any Grade:**<br>– Monitor and evaluate liver function test: AST, ALT, ALP, and TB.<br>– Evaluate for alternative etiologies (e.g., viral hepatitis, disease progression, concomitant medications, worsening of liver cirrhosis [e.g., portal vein thrombosis]).<br>– For HBV+ patients: evaluate quantitative HBV viral load, quantitative HBsAg, or HBeAg<br>– For HCV+ patients: evaluate quantitative HCV viral load<br>– Consider consulting hepatologist/Infectious disease specialist regarding change/implementation in/of antiviral medications for any patient with an elevated HBV viral load >2000 IU/ml<br>– Consider consulting hepatologist/Infectious disease specialist regarding change/implementation in/of antiviral HCV medications if HCV viral load increased by ≥2-fold<br>– For HCV+ with HBcAB+: Evaluate for both HBV and HCV as above
**Grade 1**	• No dose modifications.	

(Isolated AST or ALT >ULN and ≤5.0×ULN, whether normal or elevated at baseline)	• If ALT/AST elevations represents significant worsening based on investigator assessment, then treat as Grade 2 event.<br>For all grades, see instructions at bottom of shaded area if transaminase rise is not isolated but (at any time) occurs in setting of either **increasing bilirubin or signs of DILI/liver decompensation**	
**Grade 2**<br>(Isolated AST or ALT >5.0×ULN and ≤8.0×ULN, if normal at baseline)<br>(Isolated AST or ALT >2.0×baseline and ≤12.5×ULN, if elevated >ULN at baseline)	• Hold study drug/study regimen dose until Grade 2 resolution to Grade ≤1 or baseline.<br>• If toxicity worsens, then treat as Grade 3 or Grade 4.<br>If toxicity improves to Grade ≤1 or baseline, resume study drug/study regimen after completion of steroid taper.	**For Grade 2:**<br>– Regular and frequent checking of LFTs (e.g., every 1 to 3 days) until elevations of these are improving or resolved.<br>– Recommend consult hepatologist; consider abdominal ultrasound, including Doppler assessment of liver perfusion.<br>– Consider, as necessary, discussing with study physician.<br>– If event is persistent (>3 to 5 days) or worsens, and investigator suspects toxicity to be immune-mediated AE, recommend to start prednisone 1 to 2 mg/kg/day PO or IV equivalent.<br>– If still no improvement within 3 to 5 days despite 1 to 2 mg/kg/day of prednisone PO or IV equivalent, consider additional workup and treatment with IV methylprednisolone 2 to 4 mg/kg/day.<br>– If still no improvement within 3 to 5 days despite 2 to 4 mg/kg/day of IV methylprednisolone, consider additional abdominal workup (including liver biopsy) and imaging (i.e., liver ultrasound), and consider starting immunosuppressives (i.e., mycophenolate mofetil).[a] Discuss with study physician

		if mycophenolate mofetil is not available. **Infliximab should NOT be used.**
**Grade 3**<br>(Isolated AST or ALT >8.0×ULN and ≤20.0×ULN, if normal at baseline)<br><br>(Isolated AST or ALT >12.5×ULN and ≤20.0×ULN, if elevated >ULN at baseline)	• Hold study drug/study regimen dose until resolution to Grade ≤1 or baseline<br>• Resume study drug/study regimen if elevations downgrade to Grade ≤1 or baseline within 14 days and after completion of steroid taper.<br>• Permanently discontinue study drug/study regimen if the elevations do not downgrade to Grade ≤1 or baseline within 14 days<br><br>Permanently discontinue study drug/study regimen for any case meeting Hy's law criteria, in the absence of any alternative cause.[b]	**For Grade 3:**<br>– Regular and frequent checking of LFTs (e.g., every 1-2 days) until elevations of these are improving or resolved.<br>– Consult hepatologist (unless investigator is hepatologist); obtain abdominal ultrasound, including Doppler assessment of liver perfusion; and consider liver biopsy.<br>– Consider, as necessary, discussing with study physician.<br>– If investigator suspects toxicity to be immune-mediated, promptly initiate empiric IV methylprednisolone at 1 to 4 mg/kg/day or equivalent.<br>– If no improvement within 3 to 5 days despite 1 to 4 mg/kg/day methylprednisolone IV or equivalent, obtain liver biopsy (if it has not been done already) and promptly start treatment with immunosuppressive therapy (mycophenolate mofetil). Discuss with study physician if mycophenolate is not available. **Infliximab should NOT be used.**<br>– Once the patient is improving, gradually taper steroids over ≥28 days and consider prophylactic antibiotics, antifungals, and anti-PCP treatment (refer to current NCCN guidelines for treatment of cancer-related infections [Category 2B recommendation]).[a]
**Grade 4**<br>(Isolated AST or ALT >20×ULN, whether normal or elevated at baseline)	Permanently discontinue study drug/study regimen.	**For Grade 4:**<br>**Same as above**<br>**(except would recommend obtaining liver biopsy early)**

If transaminase rise is not isolated but (at any time) occurs in setting of either increasing total/direct bilirubin (≥1.5×ULN, if normal at baseline; or 2×baseline, if >ULN at baseline) or signs of DILI/liver decompensation (e.g., fever, elevated INR):

- **Manage dosing for Grade 1 transaminase rise as instructed for Grade 2 transaminase rise**
- **Manage dosing for Grade 2 transaminase rise as instructed for Grade 3 transaminase rise**
- **Grade 3-4: Permanently discontinue study drug/study regimen**

**Any Grade**	**General Guidance**	**For Any Grade:**<br>– Consult with nephrologist.<br>– Monitor for signs and symptoms that may be related to changes in renal function (e.g., routine urinalysis, elevated serum BUN and creatinine, decreased creatinine clearance, electrolyte imbalance, decrease in urine output, or proteinuria).<br>– Patients should be thoroughly evaluated to rule out any alternative etiology (e.g., disease progression or infections).<br>– Steroids should be considered in the absence of clear alternative etiology even for low-grade events (Grade 2), in order to prevent potential progression to higher grade event.
**Grade 1**<br>(Serum creatinine > 1 to 1.5 × baseline; > ULN to 1.5 × ULN)	No dose modifications.	**For Grade 1:**<br>– Monitor serum creatinine weekly and any accompanying symptoms.<br>• If creatinine returns to baseline, resume its regular monitoring per study protocol.<br>• If creatinine worsens, depending on the severity, treat as Grade 2, 3, or 4.

		– Consider symptomatic treatment, including hydration, electrolyte replacement, and diuretics.
**Grade 2**<br>(serum creatinine >1.5 to 3.0 × baseline; >1.5 to 3.0 × ULN)	Hold study drug/study regimen until resolution to Grade ≤1 or baseline.<br>• If toxicity worsens, then treat as Grade 3 or 4.<br>• If toxicity improves to Grade ≤1 or baseline, then resume study drug/study regimen after completion of steroid taper.	**For Grade 2:**<br>– Consider symptomatic treatment, including hydration, electrolyte replacement, and diuretics.<br>– Carefully monitor serum creatinine every 2 to 3 days and as clinically warranted.<br>– Consult nephrologist and consider renal biopsy if clinically indicated.<br>– If event is persistent (>3 to 5 days) or worsens, promptly start prednisone 1 to 2 mg/kg/day PO or IV equivalent.<br>– If event is not responsive within 3 to 5 days or worsens despite prednisone at 1 to 2 mg/kg/day PO or IV equivalent, additional workup should be considered and prompt treatment with IV methylprednisolone at 2 to 4 mg/kg/day started.<br>– Once the patient is improving, gradually taper steroids over ≥28 days and consider prophylactic antibiotics, antifungals, and anti-PJP treatment (refer to current NCCN guidelines for treatment of cancer-related infections [Category 2B recommendation]).[a]<br>– When event returns to baseline, resume study drug/study regimen and routine serum creatinine monitoring per study protocol.
**Grade 3 or 4**<br>(Grade 3: serum creatinine >3.0 × baseline; >3.0 to 6.0 × ULN;	Permanently discontinue study drug/study regimen.	**For Grade 3 or 4:**<br>– Carefully monitor serum creatinine on daily basis.<br>– Consult nephrologist and consider renal biopsy if clinically indicated.<br>– Promptly start prednisone 1 to 2 mg/kg/day PO or IV equivalent.<br>– If event is not responsive within 3 to 5 days or worsens despite prednisone at 1 to 2 mg/kg/day PO or IV equivalent, additional workup should be considered and prompt

Grade 4: serum creatinine >6.0 × ULN)		treatment with IV methylprednisolone 2 to 4 mg/kg/day started.<br>– Once the patient is improving, gradually taper steroids over ≥28 days and consider prophylactic antibiotics, antifungals, and anti-PJP treatment (refer to current NCCN guidelines for treatment of cancer-related infections [Category 2B recommendation]).[a]
**Any Grade**<br>(refer to NCI CTCAE v 4.03 for definition of severity/grade depending on type of skin rash)	**General Guidance**	**For Any Grade:**<br>– Monitor for signs and symptoms of dermatitis (rash and pruritus).<br>– IF THERE IS ANY BULLOUS FORMATION, THE STUDY PHYSICIAN SHOULD BE CONTACTED **AND STUDY DRUG DISCONTINUED.**
**Grade 1**	No dose modifications.	**For Grade 1:**<br>– Consider symptomatic treatment, including oral antipruritics (e.g., diphenhydramine or hydroxyzine) and topical therapy (e.g., urea cream).
**Grade 2**	For persistent (>1 to 2 weeks) Grade 2 events, hold scheduled study drug/study regimen until resolution to Grade ≤1 or baseline.<br>• If toxicity worsens, then treat as Grade 3.<br>• If toxicity improves to Grade ≤1 or baseline, then resume drug/study	**For Grade 2:**<br>– Obtain dermatology consult.<br>– Consider symptomatic treatment, including oral antipruritics (e.g., diphenhydramine or hydroxyzine) and topical therapy (e.g., urea cream).<br>– Consider moderate-strength topical steroid.<br>– If no improvement of rash/skin lesions occurs within 3 to 5 days or is worsening despite symptomatic treatment and/or use of moderate strength topical steroid, consider, as necessary, discussing with study physician and promptly start systemic steroids such as prednisone 1 to 2 mg/kg/day PO or IV equivalent.

	regimen after completion of steroid taper.	– Consider skin biopsy if the event is persistent for >1 to 2 weeks or recurs.
**Grade 3 or 4**	**For Grade 3:**<br>Hold study drug/study regimen until resolution to Grade ≤1 or baseline.<br>If temporarily holding the study drug/study regimen does not provide improvement of the Grade 3 skin rash to Grade ≤1 or baseline within 30 days, then permanently discontinue study drug/study regimen.<br><br>**For Grade 4:**<br>Permanently discontinue study drug/study regimen.	**For Grade 3 or 4:**<br>– Consult dermatology.<br>– Promptly initiate empiric IV methylprednisolone 1 to 4 mg/kg/day or equivalent.<br>– Consider hospitalization.<br>– Monitor extent of rash [Rule of Nines].<br>– Consider skin biopsy (preferably more than 1) as clinically feasible.<br>– Once the patient is improving, gradually taper steroids over ≥28 days and consider prophylactic antibiotics, antifungals, and anti-PJP treatment (refer to current NCCN guidelines for treatment of cancer-related infections [Category 2B recommendation]).[a]<br>– Consider, as necessary, discussing with study physician.
**Any Grade**<br>(depending on the type of endocrinopathy, refer to NCI CTCAE v4.03 for defining the CTC grade/severity)	**General Guidance**	**For Any Grade:**<br>– Consider consulting an endocrinologist for endocrine events.<br>– Consider, as necessary, discussing with study physician.<br>– Monitor patients for signs and symptoms of endocrinopathies. Non-specific symptoms include headache, fatigue, behavior changes, changed mental status, vertigo, abdominal pain, unusual bowel habits, polydipsia, polyuria, hypotension, and weakness.

		– Patients should be thoroughly evaluated to rule out any alternative etiology (e.g., disease progression including brain metastases, or infections).<br>– Depending on the suspected endocrinopathy, monitor and evaluate thyroid function tests: TSH, free T3 and free T4 and other relevant endocrine and related labs (e.g., blood glucose and ketone levels, HgA1c).<br>– For modest asymptomatic elevations in serum amylase and lipase, corticosteroid treatment is not indicated as long as there are no other signs or symptoms of pancreatic inflammation.<br>– If a patient experiences an AE that is thought to be possibly of autoimmune nature (e.g., thyroiditis, pancreatitis, hypophysitis, or diabetes insipidus), the investigator should send a blood sample for appropriate autoimmune antibody testing.
**Grade 1**	No dose modifications.	**For Grade 1 (including those with asymptomatic TSH elevation):**<br>– Monitor patient with appropriate endocrine function tests.<br>– For suspected hypophysitis/hypopituitarism, consider consultation of an endocrinologist to guide assessment of early-morning ACTH, cortisol, TSH and free T4; also consider gonadotropins, sex hormones, and prolactin levels, as well as cosyntropin stimulation test (though it may not be useful in diagnosing early secondary adrenal insufficiency).<br>– If TSH < 0.5 × LLN, or TSH >2 × ULN, or consistently out of range in 2 subsequent measurements, include free T4 at subsequent cycles as clinically indicated and consider consultation of an endocrinologist.
**Grade 2**	For Grade 2 endocrinopathy other than hypothyroidism and Type 1 diabetes mellitus, hold study drug/study regimen dose until patient is clinically stable.	**For Grade 2 (including those with symptomatic endocrinopathy):**<br>– Consult endocrinologist to guide evaluation of endocrine function and, as indicated by suspected endocrinopathy and as clinically indicated, consider pituitary scan.<br>– For all patients with abnormal endocrine work up, except those with isolated hypothyroidism or Type 1 DM, and as guided by an endocrinologist, consider short-term

	• If toxicity worsens, then treat as Grade 3 or Grade 4.<br>Study drug/study regimen can be resumed once event stabilizes and after completion of steroid taper.<br>Patients with endocrinopathies who may require prolonged or continued steroid replacement (e.g., adrenal insufficiency) can be retreated with study drug/study regimen on the following conditions:<br>1. The event stabilizes and is controlled.<br>2. The patient is clinically stable as per investigator or treating physician's clinical judgement.<br>3. Doses of prednisone are ≤10 mg/day or equivalent.	corticosteroids (e.g., 1 to 2 mg/kg/day methylprednisolone or IV equivalent) and prompt initiation of treatment with relevant hormone replacement (e.g., hydrocortisone, sex hormones).<br>– Isolated hypothyroidism may be treated with replacement therapy, without study drug/study regimen interruption, and without corticosteroids.<br>– Isolated Type 1 diabetes mellitus (DM) may be treated with appropriate diabetic therapy, without study drug/study regimen interruption, and without corticosteroids.<br>– Once patients on steroids are improving, gradually taper immunosuppressive steroids (as appropriate and with guidance of endocrinologist) over ≥28 days and consider prophylactic antibiotics, antifungals, and anti-PJP treatment (refer to current NCCN guidelines for treatment of cancer-related infections [Category 2B recommendation]).[a]<br>– For patients with normal endocrine workup (laboratory assessment or MRI scans), repeat laboratory assessments/MRI as clinically indicated.
**Grade 3 or 4**	For Grade 3 or 4 endocrinopathy other than hypothyroidism and Type 1 diabetes mellitus, hold study drug/study regimen dose until endocrinopathy symptom(s) are controlled.	**For Grade 3 or 4:**<br>– Consult endocrinologist to guide evaluation of endocrine function and, as indicated by suspected endocrinopathy and as clinically indicated, consider pituitary scan. Hospitalization recommended.<br>– For all patients with abnormal endocrine work up, except those with isolated hypothyroidism or Type 1 DM, and as guided by an endocrinologist, promptly initiate empiric IV methylprednisolone 1 to 2 mg/kg/day or equivalent, as well as relevant hormone replacement (e.g., hydrocortisone, sex hormones).

	Study drug/study regimen can be resumed once event stabilizes and after completion of steroid taper.<br>Patients with endocrinopathies who may require prolonged or continued steroid replacement (e.g., adrenal insufficiency) can be retreated with study drug/study regimen on the following conditions:<br>1. The event stabilizes and is controlled.<br>2. The patient is clinically stable as per investigator or treating physician's clinical judgement.<br>3. Doses of prednisone are ≤10 mg/day or equivalent.	– For adrenal crisis, severe dehydration, hypotension, or shock, immediately initiate IV corticosteroids with mineralocorticoid activity.<br>– Isolated hypothyroidism may be treated with replacement therapy, without study drug/study regimen interruption, and without corticosteroids.<br>– Isolated Type 1 diabetes mellitus may be treated with appropriate diabetic therapy, without study drug/study regimen interruption, and without corticosteroids.<br>– Once patients on steroids are improving, gradually taper immunosuppressive steroids (as appropriate and with guidance of endocrinologist) over ≥28 days and consider prophylactic antibiotics, antifungals, and anti-PJP treatment (refer to current NCCN guidelines for treatment of cancer-related infections [Category 2B recommendation]).[a]
**Any Grade**<br>(depending on the type of neurotoxicity, refer to NCI CTCAE v4.03 for defining the CTC grade/severity)	**General Guidance**	**For Any Grade:**<br>– Patients should be evaluated to rule out any alternative etiology (e.g., disease progression, infections, metabolic syndromes, or medications).<br>– Monitor patient for general symptoms (headache, nausea, vertigo, behavior change, or weakness).<br>– Consider appropriate diagnostic testing (e.g., electromyogram and nerve conduction investigations).<br>– Perform symptomatic treatment with neurological consult as appropriate.<br>–
**Grade 1**	No dose modifications.	**For Grade 1:**

		– See "Any Grade" recommendations above.
**Grade 2**	For acute motor neuropathies or neurotoxicity, hold study drug/study regimen dose until resolution to Grade ≤1.<br>For sensory neuropathy/neuropathic pain, consider holding study drug/study regimen dose until resolution to Grade ≤1.<br>If toxicity worsens, then treat as Grade 3 or 4.<br>Study drug/study regimen can be resumed once event improves to Grade ≤1 and after completion of steroid taper.	**For Grade 2:**<br>– Consider, as necessary, discussing with the study physician.<br>– Obtain neurology consult.<br>– Sensory neuropathy/neuropathic pain may be managed by appropriate medications (e.g., gabapentin or duloxetine).<br>– Promptly start systemic steroids prednisone 1 to 2 mg/kg/day PO or IV equivalent.<br>– If no improvement within 3 to 5 days despite 1 to 2 mg/kg/day prednisone PO or IV equivalent, consider additional workup and promptly treat with additional immunosuppressive therapy (e.g., IV IG).
**Grade 3 or 4**	**For Grade 3:**<br>Hold study drug/study regimen dose until resolution to Grade ≤1.<br>Permanently discontinue study drug/study regimen if Grade 3 imAE does not resolve to Grade ≤1 within 30 days.<br>**For Grade 4:**	**For Grade 3 or 4:**<br>– Consider, as necessary, discussing with study physician.<br>– Obtain neurology consult.<br>– Consider hospitalization.<br>– Promptly initiate empiric IV methylprednisolone 1 to 2 mg/kg/day or equivalent.<br>– If no improvement within 3 to 5 days despite IV corticosteroids, consider additional workup and promptly treat with additional immunosuppressants (e.g., IV IG).<br>– Once stable, gradually taper steroids over ≥28 days.

	Permanently discontinue study drug/study regimen.	
**Any Grade**	**General Guidance**	**For Any Grade:**<br>– The prompt diagnosis of immune-mediated peripheral neuromotor syndromes is important, since certain patients may unpredictably experience acute decompensations that can result in substantial morbidity or in the worst case, death. Special care should be taken for certain sentinel symptoms that may predict a more severe outcome, such as prominent dysphagia, rapidly progressive weakness, and signs of respiratory insufficiency or autonomic instability.<br>– Patients should be evaluated to rule out any alternative etiology (e.g., disease progression, infections, metabolic syndromes or medications). It should be noted that the diagnosis of immune-mediated peripheral neuromotor syndromes can be particularly challenging in patients with underlying cancer, due to the multiple potential confounding effects of cancer (and its treatments) throughout the neuraxis. Given the importance of prompt and accurate diagnosis, it is essential to have a low threshold to obtain a neurological consult.<br>– Neurophysiologic diagnostic testing (e.g., electromyogram and nerve conduction investigations, and “repetitive stimulation” if myasthenia is suspected) are routinely indicated upon suspicion of such conditions and may be best facilitated by means of a neurology consultation.<br>– It is important to consider that the use of steroids as the primary treatment of Guillain-Barre is not typically considered effective. Patients requiring treatment should be started with IV IG and followed by plasmapheresis if not responsive to IV IG.
**Grade 1**	No dose modifications.	**For Grade 1:**<br>– Consider, as necessary, discussing with the study physician.

		– Care should be taken to monitor patients for sentinel symptoms of a potential decompensation as described above.<br>– Obtain a neurology consult.
**Grade 2**	Hold study drug/study regimen dose until resolution to Grade ≤1.<br>Permanently discontinue study drug/study regimen if it does not resolve to Grade ≤1 within 30 days or if there are signs of respiratory insufficiency or autonomic instability.	**For Grade 2:**<br>– Consider, as necessary, discussing with the study physician.<br>– Care should be taken to monitor patients for sentinel symptoms of a potential decompensation as described above.<br>– Obtain a neurology consult<br>– Sensory neuropathy/neuropathic pain may be managed by appropriate medications (e.g., gabapentin or duloxetine).<br>*MYASTHENIA GRAVIS:*<br>○ Steroids may be successfully used to treat myasthenia gravis. It is important to consider that steroid therapy (especially with high doses) may result in transient worsening of myasthenia and should typically be administered in a monitored setting under supervision of a consulting neurologist.<br>○ Patients unable to tolerate steroids may be candidates for treatment with plasmapheresis or IV IG. Such decisions are best made in consultation with a neurologist, taking into account the unique needs of each patient.<br>○ If myasthenia gravis-like neurotoxicity is present, consider starting AChE inhibitor therapy in addition to steroids. Such therapy, if successful, can also serve to reinforce the diagnosis.<br>*GUILLAIN-BARRE:*<br>○ It is important to consider here that the use of steroids as the primary treatment of Guillain-Barre is not typically considered effective.<br>○ Patients requiring treatment should be started with IV IG and followed by plasmapheresis if not responsive to IV IG.

**Grade 3 or 4**	**For Grade 3:**<br>Hold study drug/study regimen dose until resolution to Grade ≤1.<br>Permanently discontinue study drug/study regimen if Grade 3 imAE does not resolve to Grade ≤1 within 30 days or if there are signs of respiratory insufficiency or autonomic instability.<br><br>**For Grade 4:**<br>Permanently discontinue study drug/study regimen.	**For Grade 3 or 4 (severe or life-threatening events):**<br>– Consider, as necessary, discussing with study physician.<br>– Recommend hospitalization.<br>– Monitor symptoms and obtain neurological consult.<br>*MYASTHENIA GRAVIS:*<br>○ Steroids may be successfully used to treat myasthenia gravis. They should typically be administered in a monitored setting under supervision of a consulting neurologist.<br>○ Patients unable to tolerate steroids may be candidates for treatment with plasmapheresis or IV IG.<br>○ If myasthenia gravis-like neurotoxicity present, consider starting AChE inhibitor therapy in addition to steroids. Such therapy, if successful, can also serve to reinforce the diagnosis.<br>*GUILLAIN-BARRE:*<br>○ It is important to consider here that the use of steroids as the primary treatment of Guillain-Barre is not typically considered effective.<br>○ Patients requiring treatment should be started with IV IG and followed by plasmapheresis if not responsive to IV IG.
**Any Grade**	**General Guidance**<br>Discontinue drug permanently if biopsy-proven immune-mediated myocarditis.	**For Any Grade:**<br>– The prompt diagnosis of immune-mediated myocarditis is important, particularly in patients with baseline cardiopulmonary disease and reduced cardiac function.<br>– Consider, as necessary, discussing with the study physician.<br>– Monitor patients for signs and symptoms of myocarditis (new onset or worsening chest pain, arrhythmia, shortness of breath, peripheral edema). As some symptoms can overlap

		with lung toxicities, simultaneously evaluate for and rule out pulmonary toxicity as well as other causes (e.g., pulmonary embolism, congestive heart failure, malignant pericardial effusion). A Cardiology consultation should be obtained early, with prompt assessment of whether and when to complete a cardiac biopsy, including any other diagnostic procedures.<br>– Initial work-up should include clinical evaluation, BNP, cardiac enzymes, ECG, echocardiogram (ECHO), monitoring of oxygenation via pulse oximetry (resting and exertion), and additional laboratory work-up as indicated. Spiral CT or cardiac MRI can complement ECHO to assess wall motion abnormalities when needed.<br>– Patients should be thoroughly evaluated to rule out any alternative etiology (e.g., disease progression, other medications, or infections)
**Grade 1**<br>(asymptomatic with laboratory (e.g., BNP) or cardiac imaging abnormalities)	No dose modifications required unless clinical suspicion is high, in which case hold study drug/study regimen dose during diagnostic work-up for other etiologies. If study drug/study regimen is held, resume after complete resolution to Grade 0.	**For Grade 1 (no definitive findings):**<br>- Monitor and closely follow up in 2 to 4 days for clinical symptoms, BNP, cardiac enzymes, ECG, ECHO, pulse oximetry (resting and exertion), and laboratory work-up as clinically indicated.<br>- Consider using steroids if clinical suspicion is high.
**Grade 2, 3 or 4**<br>(Grade 2: Symptoms with mild to moderate activity or exertion)<br><br>(Grade 3: Severe with symptoms at	- If Grade 2 -- Hold study drug/study regimen dose until resolution to Grade 0. If toxicity rapidly improves to Grade 0, then the decision to reinitiate study drug/study regimen will be based upon treating physician's clinical	**For Grade 2-4:**<br>– Monitor symptoms daily, hospitalize.<br>– Promptly start IV methylprednisolone 2 to 4 mg/kg/day or equivalent after Cardiology consultation has determined whether and when to complete diagnostic procedures including a cardiac biopsy.<br>– Supportive care (e.g., oxygen).<br>– If no improvement within 3 to 5 days despite IV methylprednisolone at 2 to 4 mg/kg/day, promptly start immunosuppressive therapy such as TNF inhibitors

rest or with minimal activity or exertion; intervention indicated)<br><br>(Grade 4: Life-threatening consequences; urgent intervention indicated (e.g., continuous IV therapy or mechanical hemodynamic support))	judgment and after completion of steroid taper. If toxicity does not rapidly improve, permanently. discontinue study drug/study regimen.<br><br>If Grade 3-4, permanently discontinue study drug/study regimen.	(e.g., infliximab at 5 mg/kg every 2 weeks). Caution: It is important to rule out sepsis and refer to infliximab label for general guidance before using infliximab.<br>– Once the patient is improving, gradually taper steroids over ≥28 days and consider prophylactic antibiotics, antifungals, or anti-PJP treatment (refer to current NCCN guidelines for treatment of cancer-related infections [Category 2B recommendation]).[a]

Appendix 2. Study Flow Chart

**Trial Period:**	**Screening Phase**	**Treatment Cycles[a]**							**Post-Treatment**	
**Treatment Cycle/Title:**	**Study Screening[c]**								**Safety Follow-up Follow Up Visits**	**Survival Follow-Up**
		**1**	**2**	**3**	**4**	**5**	**6**	**Cycle 7 to discontinuation**		
**Scheduling Window (Days)[b]**	**-28 to -1**	**±3**	**± 3**	**± 3**	**± 3**	**± 3**	**± 3**	**± 3**	**~30 days post-discontinuation**	**Every 3 months (± 2 weeks)**
**Administrative Procedures**										
Informed Consent	X									
Inclusion/Exclusion Criteria	X									
Demographics and Medical History	X									
Prior and Concomitant Medication Review	X	X	X	X	X	X	X	X	X	
Trial Treatment Administration		X	X	X	X	X	X	X		
Post-study anticancer therapy status									X	X
**Clinical Procedure/Assessments**										

**Trial Period:**	**Screening Phase**	**Treatment Cycles[a]**							**Post-Treatment**	
**Treatment Cycle/Title:**	**Study Screening[c]**	**1**	**2**	**3**	**4**	**5**	**6**	**Cycle 7 to discontinuation**	**Safety Follow-up Follow Up Visits**	**Survival Follow-Up**
**Scheduling Window (Days)[b]**	**-28 to -1**	**±3**	**± 3**	**± 3**	**± 3**	**± 3**	**± 3**	**± 3**	**~30 days post-discontinuation**	**Every 3 months (± 2 weeks)**
Survival Status										X
Review Adverse Events	X								X	
Physical Examination	X	X	X	X	X	X	X	X	X	
Vital Signs and Weight	X	X	X	X	X	X	X	X	X	
ECOG Performance Status	X	X	X	X	X	X	X	X	X	
**Laboratory Procedures[d]**										
Pregnancy Test – Urine or Serum -HCG[d]	X									
PT/INR and aPTT	X	As clinically indicated								
CBC with Differential	X		X	X	X	X	X	X		
Comprehensive Serum Chemistry Panel	X		X	X	X	X	X	X	X	
T3, FT4 and TSH	X			X		X		Every 4 cycles		

**Trial Period:**	**Screening Phase**	**Treatment Cycles[a]**							**Post-Treatment**	
**Treatment Cycle/Title:**	**Study Screening[c]**	**1**	**2**	**3**	**4**	**5**	**6**	**Cycle 7 to discontinuation**	**Safety Follow-up Follow Up Visits**	**Survival Follow-Up**
**Scheduling Window (Days)[b]**	**-28 to -1**	**±3**	**± 3**	**± 3**	**± 3**	**± 3**	**± 3**	**± 3**	**~30 days post-discontinuation**	**Every 3 months (± 2 weeks)**
								(9, 13, etc)		
Hepatitis B & C (HBs Ag, Anti-HBc, HCV RNA)	X									
Urinalysis	X			X		X		Every 4 cycles (9, 13, etc)		
EKG	X	when clinically indicated								
Echo	X									
**Efficacy measurement**										
Tumor Imaging[e]	X	Every 6 weeks (±7 days) for first 24 weeks, then 12 weeks thereafter						Every 12 weeks (±7 days)	X[g]	
Chest X-ray				X		X		Every 4 cycles (9, 13, etc)		

**Trial Period:**	**Screening Phase**	**Treatment Cycles**[a]							**Post-Treatment**	
**Treatment Cycle/Title:**	**Study Screening**[c]								**Safety Follow-up Follow Up Visits**	**Survival Follow-Up**
		**1**	**2**	**3**	**4**	**5**	**6**	**Cycle 7 to discontinuation**		
**Scheduling Window (Days)**[b]	**-28 to -1**	**±3**	**± 3**	**± 3**	**± 3**	**± 3**	**± 3**	**± 3**	**~30 days post-discontinuation**	**Every 3 months (± 2 weeks)**
**Tissue/Blood biomarker collection**[g]										
Whole blood sample for gDNA[f]	X			X					(X)	
Blood cytokine	X		X						(X)	
Tumor tissue[h]	X [i]			(X)					(X)	

a. Unless otherwise specified, assessments/procedures are to be performed on Day 1 and prior to the first dose of treatment for each cycle.

b. Unless otherwise specified, the window for each visit is ± 3 days.

c. Laboratory tests for screening are to be performed within 14 days prior to the first dose of trial treatment. After Cycle 1, lab samples can be collected up to ±3 days prior to the scheduled time point.

d. For women of reproductive potential, a negative pregnancy test should be confirmed within 7 days prior to first dose of trial treatment to be eligible for the trial

e. Baseline tumor imaging will be performed within 28 days prior to the first dose of trial treatment Scans performed as part of routine clinical management are acceptable for use as the baseline scan if they are of diagnostic quality and performed within the allotted screening window (± 7 days).

After 24 weeks, imaging time point will occur every 12 weeks (± 7 days). Imaging timing should follow calendar days and should not be adjusted due to dose interruptions. The same imaging technique, acquisition, and processing parameters should be used in a subject throughout the trial.

f. Informed consent for the optional future biomedical research samples must be obtained before the DNA sample is collected.

g. In subjects who discontinue study therapy without confirmed disease progression, a radiologic evaluation should be performed within 12 weeks of treatment discontinuation.

Every effort should be made to continue monitoring their disease status by radiologic imaging every 12 weeks (± 7 days) until (1) the start of new anti-cancer treatment, (2) diseaseprogression, (3) death, or (4) the end of the study, whichever occurs first. For subjects who discontinue study due to progression, then a scan is not required.

h. Baseline fresh or archived tumor biopsy will be obtained prior to the initiation of study for all cases. On-treatment (cycle 3 and 9) and post-treatment tissue collection will be done for the available cases (X).
