# Supplementary material for: Durvalumab plus pazopanib combination in patients with advanced soft tissue sarcomas: a phase II trial
Source: Nat Commun. 2024 Jan 23;15:685. doi: 10.1038/s41467-024-44875-2 (PMC10806253; doi:10.1038/s41467-024-44875-2)
Supplement: Supplementary file 3 — Description of Additional Supplementary Files [file 41467_2024_44875_MOESM3_ESM.pdf]

Description of Additional Supplementary Files

Supplementary Data 1. Detected somatic mutation list

Supplementary Data 2. Read counts from RNA-seq
